# Belief in Gender Role Stereotypes Moderates the Use of Gender Typicality Cues when Making Sexual Orientation Judgements from Faces

**DOI:** 10.1007/s10508-024-03046-6

**Published:** 2024-12-14

**Authors:** Jessica K. De La Mare, Maisie G. Taylor, Anthony J. Lee

**Affiliations:** Division of Psychology, Faculty of Natural Sciences, University of Stirling, Stirling, FK9 4LA Scotland

**Keywords:** Sexual orientation, Social categorisation, Face perception, Gender stereotypes, Sexual prejudice

## Abstract

People use cues of facial gender typicality when making sexual orientation judgements, where gender typical faces (masculine men and feminine women) are more likely to be judged as heterosexual and gender atypical faces (feminine men and masculine women) are judged as non-heterosexual. Individual differences in the belief of associated stereotypes have been shown to influence how these stereotypes are used to make social judgments of others; therefore, across two studies, we tested whether the strength of beliefs in gender stereotypes impacted how facial gender typicality cues were used when making sexual orientation judgements. In both Study 1 and 2 (*n* = 283 and 219, respectively), participants made sexual orientation judgements of 80 faces (40 male, 40 female) that varied in gender typicality and completed a measure of belief in gender stereotypes. In Study 2, participants also completed a sexual prejudice measure. In line with predictions, both studies found that the strength in belief of gender stereotypes significantly moderated the use of facial gender typicality cues when making sexual orientation judgements. Participants with a greater belief in gender stereotypes were more likely to judge a face as heterosexual as gender typicality increased. In Study 2, the association between sexual prejudice and use of gender typicality cues was fully mediated by beliefs in gender role stereotypes. These results highlight the importance of considering individual differences of the perceiver and how they can interact with cues from a target, particularly when making sexual orientation judgements.

**Supplementary Information:**

The online version contains supplementary material available at 10.1007/s10508-024-03046-6.

## Introduction

People quickly and automatically categorize others into social groups, and sometimes these categories are perceptually obvious, (e.g., judgements of age, race, and sex are made with near-perfect accuracy; Macrae & Bodenhausen, 2000). However, sometimes these categories are ambiguous. One such perceptually ambiguous social category is sexual orientation, which can be defined by the level of romantic or sexual attraction an individual has for others of the same or opposite sex, or of the same or a different gender. The ability to categorize others by their sexual orientation can inform individuals on how they should proceed in a social interaction, for example, when approaching someone in a romantic context. Judgements of sexual orientation can be made rapidly from brief presentations (as little as 50 ms) of bodies (Ambady et al., 1999; Johnson et al., 2007), voices (Rieger et al., 2010), and faces (Rule & Ambady, 2008; Rule et al., 2009). This would suggest that sexual orientation judgements are made with little or no conscious thought, however, these effects require further replications.

Previous research has identified several cues that perceivers may use to help make judgements of sexual orientation; these include speech (e.g., pitch), behavioral choices (e.g., clothing), and structural morphology (e.g., facial information; for a review, see Rule, 2017). One key cue that individuals use when making sexual orientation judgements is gender typicality (i.e., how closely an individual resembles their own gender). Homosexual men are generally perceived as more feminine compared to heterosexual men, while homosexual women are perceived as more masculine than heterosexual women (Blashill & Powlishta, 2009; Kite & Deaux, 1987; Madon, 1997). The perceived gender typicality of faces has been found to influence sexual orientation judgements (when effect sizes are reported, this effect is small to medium; Bjornsdottir & Rule, 2020; Lick & Johnson, 2014, 2016; Stern et al., 2013). Furthermore, individuals tend to judge faces as homosexual when they are gender inverted on face shape and texture (Freeman et al., 2010). Some evidence suggests that the stereotype that gender atypicality is associated with non-heterosexuality may not be entirely unfounded. Homosexual men tend to be more feminine and homosexual women more masculine than their heterosexual counterparts, specifically, gender nonconformity in childhood behaviors (Bailey & Zucker, 1995; Li et al., 2017; Rieger et al., 2008; Xu et al., 2019) and childhood photographs (Watts et al., 2018) predicts non-heterosexuality in adulthood. Some research has even indicated that the gender typicality of certain facial features (e.g., the nose or forehead shape) is associated with homosexuality (Skorska et al., 2015), and can be used by others to infer sexual orientation (González-Álvarez, 2017; González-Álvarez & Sos‐Peña, 2022).

While sexual orientation judgements appear to be consistent across some cultures (Rule et al., 2011), there is also significant individual variation. For example, while there is an overall bias toward categorizing others as heterosexual (Lick & Johnson, 2016), non-heterosexual individuals appear to have less bias toward evaluating others as heterosexual (Ambady et al., 1999; Johnson & Ghavami, 2011; Rule et al., 2007, but see Bjornsdottir et al., 2022). In addition, differences in political ideology appear to be associated with sexual orientation judgements; Stern et al. (2013) found that conservatives were more likely than liberals to use cues relating to gender typicality. Also, liberals took longer than conservatives to make sexual orientation judgements, which suggests they may adjust initial judgements that are based on stereotypes. Indeed, when cognitively overloaded and therefore were unable to reassess their initial judgements, there were no differences in use of gender typicality cues between liberals and conservatives (Stern et al., 2013). However, further research is needed to validate these findings. Altogether, this would suggest that not only do individual’s preconceived stereotypes influence sexual orientation judgements, but that there are individual differences in the use of these stereotypes. Indeed, participants’ acceptance of the “gaydar” myth (i.e., the belief in popular culture that there exists an ability or intuition associated with the ability to make judgements of the sexual orientation of others) appears to influence how strongly individuals use stereotypes relating to gender to make judgements of sexual orientation (Cox et al., 2016).

Individual differences in the belief and acceptance of stereotypes appear to influence how often, and to what extent, these stereotypes are used to make judgements of others (Carter et al., 2006). This includes judgements based on gender stereotypes. For example, individuals with traditional gender views tend to evaluate those who conform to gender stereotypical family roles (e.g., primary caregiver mothers and breadwinning fathers) more favorably compared to those in stereo-atypical family roles (Gaunt, 2013). Specific to sexual orientation judgements, an effect of gender stereotypes associated with emotional expression has been found, where individuals expressing emotions that do not align with their gender stereotype (i.e., angry women, smiling men) were more likely to be perceived as homosexual (Bjornsdottir & Rule, 2020). As cues of gender typicality are used to make judgements of sexual orientation (Blashill & Powlishta, 2009; Kite & Deaux, 1987; Madon, 1997), individual differences in the strength of belief in gender stereotypes are likely to impact sexual orientation judgements (e.g., Stern et al., 2013). Therefore, we could predict that individuals with stronger beliefs in gender stereotypes are more likely to rely on automatic stereotypes, for example, those relating to sexual orientation and gender typicality, when making judgements of sexual orientation.

All individuals may be equally aware of cultural stereotypes, but those who are less prejudiced toward the stereotyped group may be more likely to inhibit the use of these stereotypes (Devine, 1989). Indeed, gender typicality appears to influence the evaluations given by individuals who report different levels of prejudice toward homosexuality. Highly prejudice individuals tended to report gender atypical homosexual individuals more unfavorably than homosexual individuals who were more typical of their gender (Lehavot & Lambert, 2007). Moreover, greater endorsement of traditional gender roles is associated with less positive attitudes toward homosexuality (Whitley, 2001; Wilkinson, 2006). Therefore, individuals with greater sexual prejudice may also be more likely to rely on cues relating to gender typicality when making judgements of sexual orientation.

Here, across two studies, we investigate the use of facial gender typicality cues when making sexual orientation judgements, and how the use of these cues may vary depending on participant’s beliefs in gender stereotypes. We predict the following:

### Hypothesis 1

Participants will use facial gender typicality cues when making judgements of sexual orientation, such that masculine male faces and feminine female faces are more likely to be judged as heterosexual, while feminine male faces and masculine female faces are more likely to be judged as non-heterosexual.

### Hypothesis 2

The above effect is moderated by participant’s endorsement of gender role stereotypes, such that those with a greater belief of gender role stereotypes will be more likely to use facial cues of gender typicality when making sexual orientation judgements.

### Hypothesis 3

In Study 2, we also include a measure of participant sexual prejudice. Therefore, we predict that participants with a greater sexual prejudice will be more likely to use cues of facial gender typicality when making sexual orientation judgements.

## Study 1

## Method

### Participants

Participants (*n* = 291) were online volunteers recruited via social media who received no incentive for their participation, or undergraduate students who participated for course credit. Participants with missing data on key variables were removed (*n* = 8). The final analysis consisted of 283 participants (195 female, 74 male, 10 non-binary, and 4 other/undisclosed; *M* = 21.97 years, SD = 6.39 years). Participants were predominantly heterosexual (*n* = 194) with the remainder identifying as homosexual (*n* = 20), bisexual (*n* = 58), or other/undisclosed (*n* = 11).

### Measures

#### Gender Role Stereotypes Scale

Beliefs in gender role stereotypes was measured using the Gender Role Stereotypes Scale (GRSS; Mills et al., 2012). Participants rated whether they believed 8 tasks should be done by a man or a woman when they are in a relationship on a 5-point scale (1 = should always be done by the man, 5 = should always be done by the woman). These tasks include asking who should mow the lawn, prepare meals, or propose marriage. Items with stereotypically male tasks were reversed-coded and items were summed, such that higher scores on the GRSS indicated greater beliefs in gender role stereotypes. Possible scores range from 8 to 40. Cronbach’s alpha indicated good reliability during development (*α* = 0.75–0.78; Mills et al., 2012).

#### Sexual Orientation Judgement Task

Participants were asked to judge the sexual orientation of 80 facial images (40 male and 40 female). Facial images were of White individuals selected from the Chicago Face Database (Ma et al., 2015). Using the accompanying norming data, faces that were the 20 highest scoring faces on either perceived masculinity or femininity were included in this study (for more detail on the rating process, see Ma et al., 2015). This resulted in the selection of 40 gender typical face (i.e., the 20 most masculine male faces and 20 most feminine female faces) and 40 gender atypical faces (i.e., the 20 most feminine male faces and 20 most masculine female faces). When presented to participants, faces were blocked by sex, with block order randomized and the order of faces within each block also randomized. Participants were instructed to categorize each face by selecting one of two on-screen buttons labeled as either “heterosexual” or “non-heterosexual. Participants were told that in this study, heterosexual is used to describe a person who is only sexually/romantically attracted to someone of the opposite gender, while non-heterosexual is used to describe any sexuality that is not heterosexual, including but not limited to gay, lesbian, bisexual, and pansexual. The facial image and categorical response options (heterosexual/non-heterosexual) were presented simultaneously and there were no time constraints on how long participants were given to view the image and select their response.

### Procedure

Participants completed the study via an online survey. After providing informed consent, participants completed the GRSS (Mills et al., 2012) and the sexual orientation judgement task presented in a random order.

### Statistical Analysis

All analyses were conducted using R statistical software (R Core Team, 2024). Data were analyzed using binomial mixed effects models conducted using the lme4 (Bates et al., 2014) and lmerTest (Kuznetsova et al., 2017) packages. The outcome variable was whether participants judged a face as heterosexual or non-heterosexual (coded as 0 and 1 respectively). The fixed effects were the z-standardized scores on the GRSS (Mills et al., 2012), the gender typicality of the face (gender atypical faces coded as − 0.5 and gender typical faces as 0.5), and the interaction between the two. The grouping variables for the random effects included participant ID and facial image ID following best practice (DeBruine & Barr, 2021). Random intercepts and slopes were specified maximally according to Barr et al. (2013) and Barr (2013). All data and analysis code are available on the Open Science Framework (OSF) at osf.io/njf3p/.

## Results

**Fig. 1:**
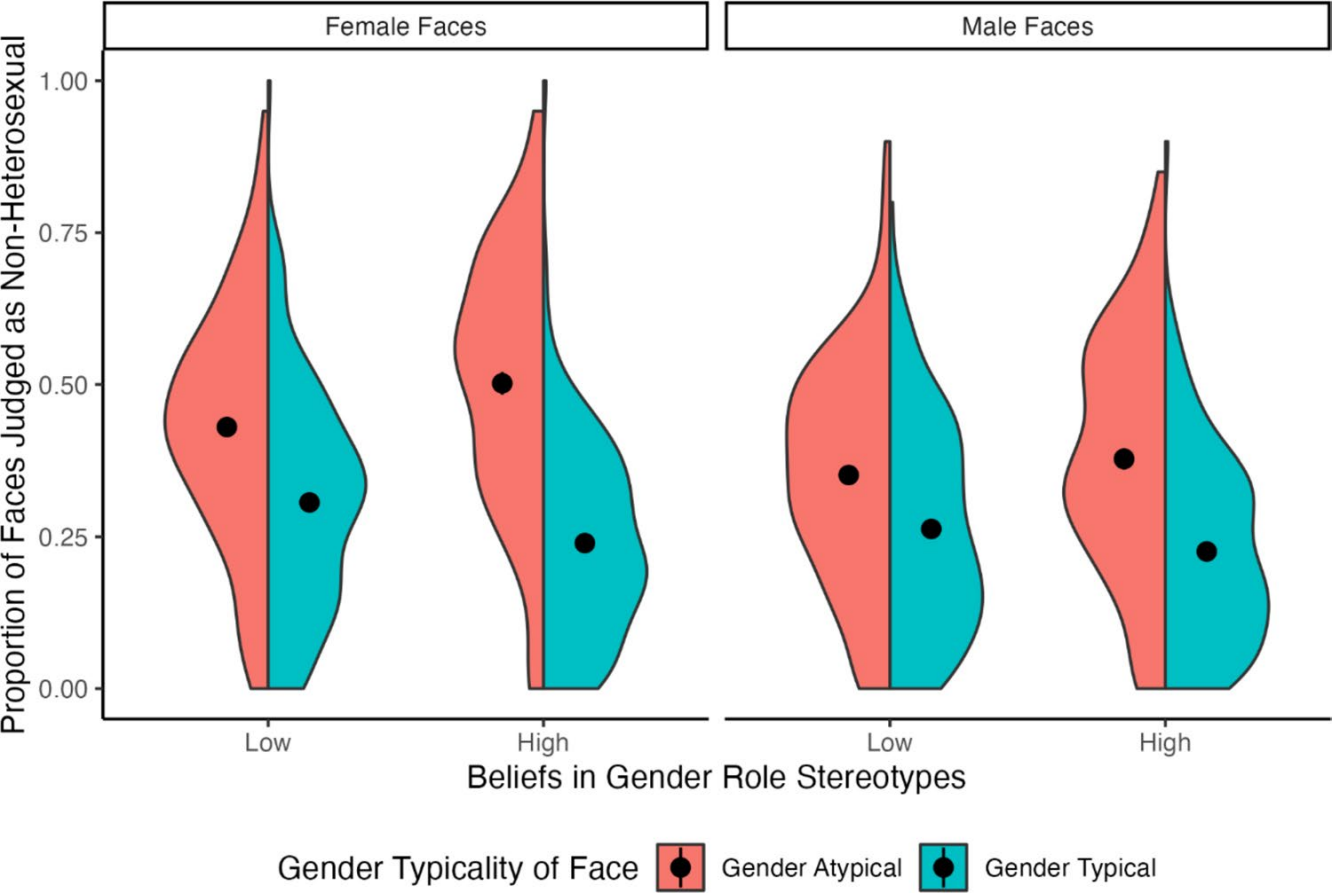
The association between participant belief in gender stereotypes, face gender typicality, and judgements of sexual orientation separated by face sex

The estimated fixed effects for all models are reported in Table 1 (for full model results, including estimated random effects, see the Supplementary Materials). We found no significant main effect of participant belief in gender stereotypes, which suggests that the participants’ belief in stereotypes did not influence the overall likelihood to judge a face as heterosexual or non-heterosexual. However, there was a significant main effect of facial gender typicality, indicating that as gender typicality increases so does the overall likelihood a face will be judged as heterosexual rather than non-heterosexual. In line with predictions, there was also a significant interaction effect between participant belief in gender stereotypes and facial gender typicality, such that participants with a greater belief in gender stereotypes were more likely to use facial gender typicality when making sexual orientation judgements (see Fig. 1). As a robustness check, we conducted separate models that included only male and only female faces, which did not change the pattern of results (full results for these models are reported in the Supplementary Materials).

**Table 1:** Fixed effects estimates predicting participants response (heterosexual or non-heterosexual) depending on participant gender roles stereotype score, and face gender typicality

	Estimate (std error)	*Z* value	*p* value
Intercept	− 0.84 (0.07)	− 12.12	**< 0.001**
Participant gender stereotype score	− 0.04 (0.05)	− 0.87	0.420
Face gender typicality	− 0.81 (0.11)	− 7.20	**< 0.001**
Participant gender stereotype score × Face gender typicality	− 0.31 (0.06)	5.11	**< 0.001**

Bold font indicates a statistically significant effect of *p* < .05

## Discussion

In line with previous literature and Hypothesis 1, cues of gender typicality were used to make sexual orientation judgements. Masculine men and feminine women were more likely to be judged as heterosexual, whereas feminine men and masculine women were more likely to be judged as non-heterosexual (Blashill & Powlishta, 2009; Kite & Deaux, 1987; Lick & Johnson, 2014, 2016; Stern et al., 2013). Further, a significant interaction was reported between participant gender role stereotype score and facial gender typicality score. This finding suggests that, as participant gender stereotypes score increases, the likelihood of gender typical faces being rated as heterosexual, and of gender atypical faces as non-heterosexual, increases, supporting Hypothesis 2.

While results from Study 1 support our hypotheses, there are numerous methodological decisions that limit the study’s conclusion. First, Study 1 only included White faces, which limits the generalisability of results. Second, faces were categorized as either gender typical or gender atypical, with only the highest scoring faces on perceived masculinity/femininity being included in the study. Given that masculinity/femininity is a continuum, including the full range of facial gender typicality would improve the ecological validity of results. Finally, we only included a measure of beliefs in gender role stereotypes. Given that beliefs in gender role stereotypes and sexual prejudice are correlated (Whitley, 2001; Wilkinson, 2006), it is unclear whether this effect could instead be explained by attitudes toward homosexuality more generally. Indeed, we could predict that the use of gender typicality cues may be moderated by participant sexual prejudice. Individuals with higher sexual prejudice may be more likely to fail to inhibit stereotypes associated with sexual orientation than individuals with lower sexual prejudice (i.e., that homosexual individuals are more likely to be gender atypical; Blashill & Powlishta, 2009; Kite & Deaux, 1987). As such, we decided to conduct Study 2, which serves as a replication and extension to Study 1.

Similar to Study 1, participants in Study 2 were shown faces and asked to make judgements of sexual orientation. However, the facial images in Study 2 were more varied, both in ethnicity (including White, Black, Latinx, and Asian) and covering the full spectrum of facial gender typicality. In addition, gender typicality was calculated using two methods: one using perceived ratings of masculinity/femininity (as used in Study 1), the other using an objective shape sexual dimorphism score calculated using geometric morphometric (the statistical analysis of shape) methods (see Holzleitner & DeBruine, 2021; Komori et al., 2011; Zelditch et al., 2012) following procedures used in previous literature (e.g., Dong et al., 2023; Leger et al., 2023). Finally, we also included a measure of sexual prejudice as well as beliefs in gender role stereotypes. The methods and predictions for Study 2 are pre-registered at osf.io/nqjcf.

## Study 2

## Method

### Participants

A total of 219 (98 women, 88 men, 18 non-binary, 15 other/undisclosed) participants were online volunteers recruited via social media, or undergraduate students who participated for course credit (*M* = 25.86 years, SD = 8.62 years). Participants reported their sexual orientation as heterosexual (*n* = 98), homosexual (*n* = 23), bisexual (*n* = 59), other (*n* = 27), or undisclosed (*n* = 12). A power analysis via simulation was conducted in *R* to determine the minimum sample size and stimuli set size. Data was simulated with a medium interaction effect (*r* = 0.30) and a random effect structure matching that of Study 1. To detect this effect with 80% power at the standard 0.05 alpha error probability, a minimum of 60 participants and 40 stimuli were required. As our analysis plan included investigating the effect of participant gender and face sex, a minimum of 120 participants (60 men, 60 women) and 80 faces (40 male, 40 female) were required.

### Measures

#### Gender Role Stereotypes Scale

As in Study 1, participants completed the GRSS (Mills et al., 2012) to measure belief in gender role stereotypes. Cronbach’s alpha indicated good reliability in the current sample (*α* = 0.81).

#### Sexual Prejudice Scale

To measure attitudes toward homosexuality, participants completed the Sexual Prejudice Scale (SPS; Chonody, 2013). The SPS is comprised of three sub-scales (stereotyping, affective-valuation, and social equality beliefs), for gay men and lesbian women separately. There are a total of 30 statements on which participants rated their agreement on a 6-point scale (1 = strongly disagree, 6 = strongly agree). These statements include items such as “Most lesbians prefer to dress like men.” and “It’s wrong for men to have sex with men.” Usually, global scores are calculated toward gay men and lesbian women separately by summing the three sub-scales. However, as the global scores for gay men and lesbian women were highly correlated (*r*(167) = 0.95, *p* < 0.001), we summed these to create a total score for the SPS, such that greater scores indicated greater sexual prejudice. Cronbach’s alpha indicated good reliability during development (gay men scale, *α* = 0.94; lesbian scale, *α* = 0.95; Chonody, 2013), and in the current sample (gay men scale, *α* = 0.95, lesbian scale, *α* = 0.93, total scale, *α* = 0.97).

#### Sexual Orientation Judgement Task

As in Study 1, participants viewed 80 facial images (40 male and 40 female) from the Chicago Face Database (Ma et al., 2015) and were asked to indicate whether they perceived the face to depict someone who was heterosexual or non-heterosexual. However, building upon Study 1, the faces were more varied. Faces were randomly selected to depict a wider range of facial masculinity and femininity (rather than only including the most masculine/feminine faces), and included individuals of multiple ethnicities, including Black, White, Asian, and Latinx (10 each per sex and ethnicity). As in Study 1, faces were presented in a random order blocked by sex.

Gender typicality of the faces used in the task were measured in two ways. First, perceived gender typicality was calculated using the masculinity and femininity ratings obtained from the Chicago Face Dataset norming data (Ma et al., 2015). There was a strong negative correlation between masculinity and femininity scores (*r*(78) = .− 94, *p* < 0.001). Femininity rating scores were reverse-coded and averaged with the masculinity ratings. Then, for female faces, this value was reverse-coded so that a higher score indicated greater facial gender typicality. Secondly, facial gender typicality was calculated using objective shape sexual dimorphism scores was calculated using geometric morphometric methods using the facefuns package in *R* (Holzleitner & DeBruine, 2021). This was done by first identifying 155 landmarks coordinates on all faces in the Chicago Face database (see Singh et al., 2023 for details). Shape sexual dimorphism was calculated by computing a linear vector between the average male and average female face shape. Then, the 80 faces used in this study are projected onto this vector, producing a sexual dimorphism score for each face. Typically, higher scores indicate greater facial masculinity; however, we reverse-coded the sexual dimorphism scores for female faces so that higher scores indicated greater objective gender typicality. For both facial gender typicality measures, scores were standardized by sex.

### Procedure

The procedure for Study 2 was almost identical to Study 1. However, Study 2 included the SPS (Chonody, 2013) presented randomly alongside the GRSS (Mills et al., 2012) and Sexual Orientation Judgement Task.

### Statistical Analysis

Similar to Study 1, all data were analyzed using binomial mixed effects modeling using the lme4 (Bates et al., 2014), and lmerTest (Kuznetsova et al., 2017) packages in the *R* statistical software (R Core Team, 2024). Separate models were conducted for each of the participant measures (gender role stereotype and sexual prejudice measures) due to multicollinearity issues. The outcome variable was whether participants selected heterosexual or non-heterosexual in the sexual orientation judgement task (coded as 0 and 1, respectively). The fixed effects were participant belief in gender stereotypes score (measured by the GRSS; Mills et al., 2012) or participant sexual prejudice score (measured by the SPS; Chonody, 2013), alongside both the perceived and objective shape facial gender typicality scores. All predictors were z-standardized at the appropriate level. The grouping variables for the random effects included participant ID and facial image ID with random slopes specified maximally (Barr, 2013; Barr et al., 2013). To retain maximum power for each analysis, missing data were removed pairwise. The gender role stereotype model included 175 participants, and the sexual prejudice model included 166 participants. All data and analysis code are available on the OSF at osf.io/njf3p/.

## Results

### Checks for Multicollinearity

To assess multicollinearity among predictors, correlations were conducted between the two participant measures and between the measures of gender typicality. There was a strong, positive correlation between participant GRSS and SPS scores (*r*(167) = 0.63, *p* < 0.001), indicating potential collinearity between these predictors. Here, we report separate models for the GRSS and SPS scores; however, as a robustness check, a model including both measures simultaneously was conducted. The pattern of results were the same as the separate models (except where noted); for full results, see Supplementary Materials. There was also a strong correlation reported between the objective sexual dimorphism measure of gender typicality and that based on perceived masculinity/femininity (*r*(78) = 0.69, *p* < 0.001). However, once gender typicality scores were standardized by sex, levels of multicollinearity were more acceptable (*r*(78) = 0.36, *p* < 0.001). Therefore, we include both gender typicality scores in the same model. However, as an additional robustness check, we have also conducted models with each gender typicality score separately, though this does not impact the pattern of results (for full results, please see the Supplementary Materials).

### Main Analysis (Pre-registered)

The estimated fixed effects for the both the gender role stereotype model and the sexual prejudice model are reported in Table 2 (for full model results, including estimated random effects, see the Supplementary Materials). Similar to Study 1, no significant main effect of participant gender role stereotype was found. There was also no significant main effect for the sexual prejudice score. This would suggest that neither of these participant measures influences the overall likelihood of judging a face as heterosexual or non-heterosexual. Also consistent with Study 1, significant negative main effects of gender typicality were found for both the perceived and objective scores. This finding indicates that as face gender typicality increases, participants were more likely to judge a face as heterosexual.

**Table 2:** Fixed effects estimates predicting participants response (heterosexual or non-heterosexual) depending on participant gender roles stereotype score or sexual prejudice score separately

	Gender Role Stereotypes Scale			Sexual Prejudice Scale		
	Estimate (std error)	*Z* value	*p* value	Estimate (std error)	*Z* value	*p* value
Intercept	− 1.18 (0.12)	− 9.68	**< 0.001**	− 1.22 (0.13)	− 9.43	**< 0.001**
Participant score	− 0.03 (0.11)	− 0.23	0.816	− 0.16 (0.12)	− 1.31	0.190
Objective face gender typicality score	− 0.13 (0.06)	− 2.11	**0.035**	− 0.13 (0.07)	− 2.01	**0.044**
Perceived face gender typicality score	− 0.19 (0.06)	− 2.97	**0.003**	− 0.20 (0.07)	− 2.93	**0.003**
Participant score × Objective face gender typicality score	0.02 (0.03)	0.62	0.533	0.03 (0.04)	0.78	0.428
Participant score × Perceived face gender typicality score	− 0.16 (0.04)	− 4.41	**< 0.001**	− 0.09 (0.04)	− 2.01	**0.045**

Bold font indicates a statistically significant effect of *p* < .05

**Fig. 2:**
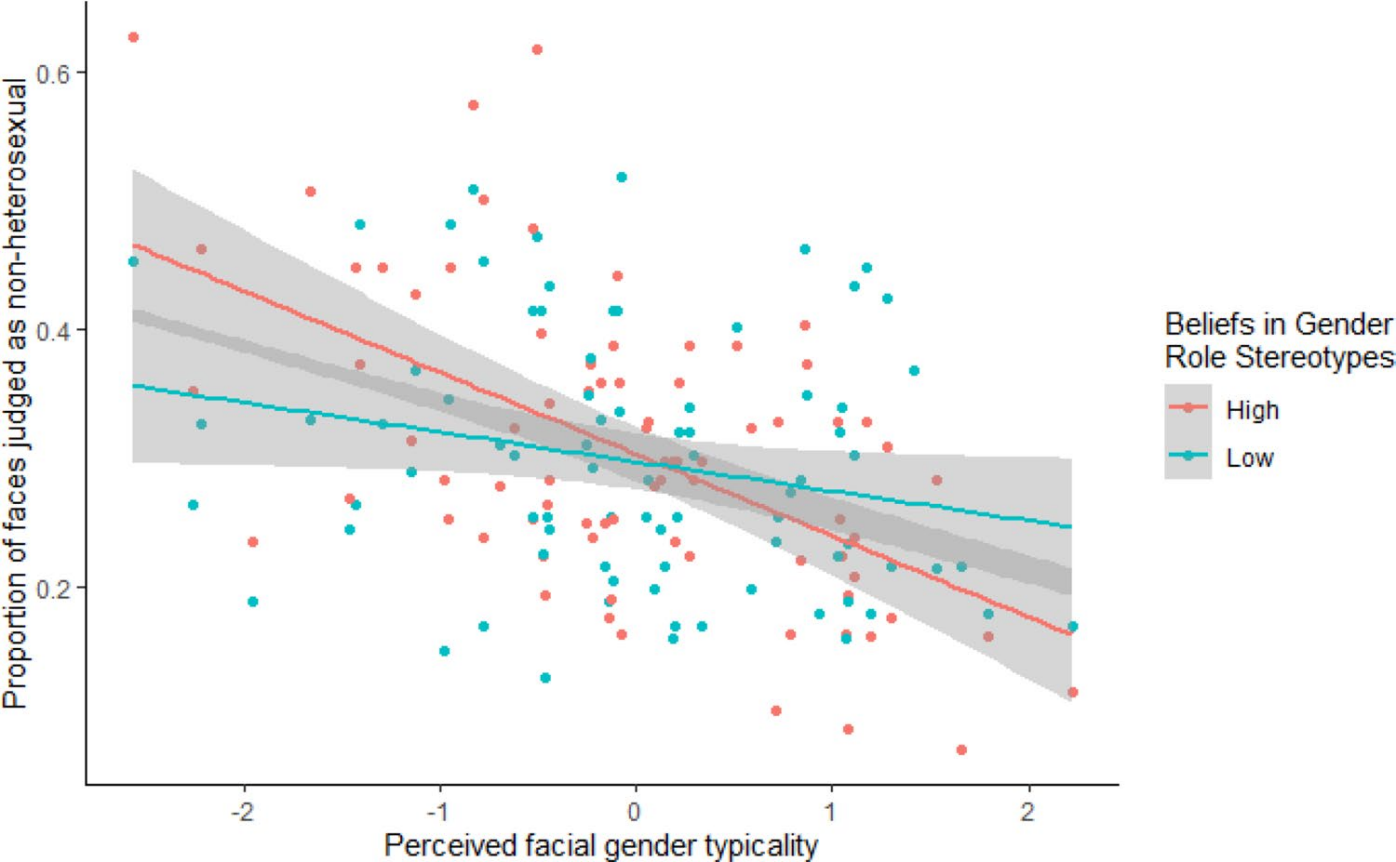
The association between mean participant response (0 = heterosexual, 1 = non-heterosexual) and perceived facial gender typicality separated by gender role stereotype score, high (red) and low (blue)

**Fig. 3:**
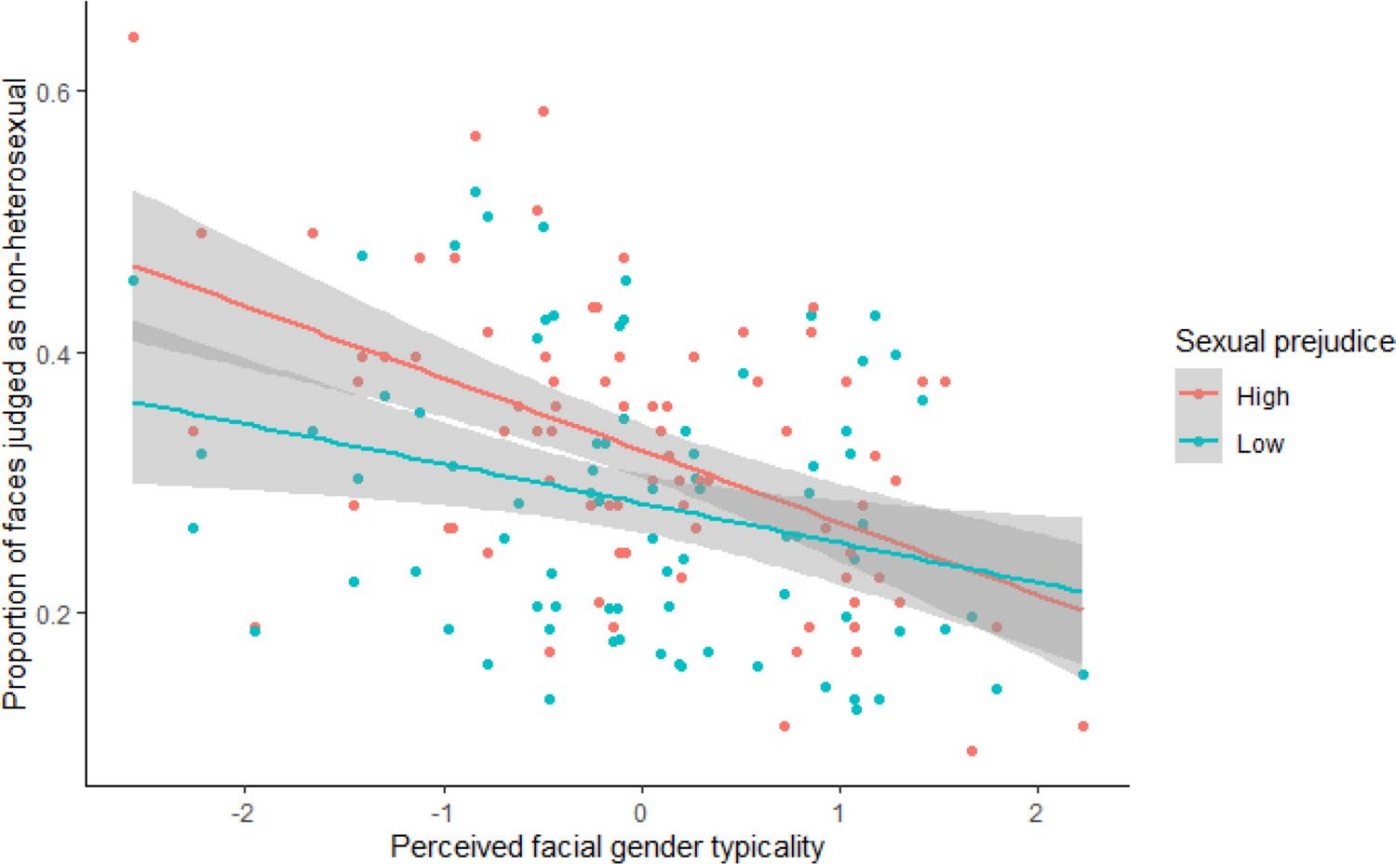
The association between mean participant response (0 = heterosexual, 1 = non-heterosexual) and perceived facial gender typicality separated by sexual prejudice score, high (red) and low (blue)

Replicating results from Study 1, we found a significant interaction between perceived gender typicality and gender role stereotype score (see Fig. 2). As predicted, participants who had greater beliefs in gender role stereotypes were more likely to judge faces as heterosexual as the face gender typicality increased. This effect was independent of sexual prejudice more generally, which also had a significant interaction effect (see Fig. 3). As participant sexual prejudice increased, so did the likelihood of judging a gender typical face as heterosexual. However, note that in the model that included both belief in gender stereotypes and sexual prejudice, this interaction was not significant. Surprisingly, there was no significant interaction effect of either beliefs in gender roles stereotypes or sexual prejudice with the objective shape gender typicality score.

To address convergence issues, the models described above were simplified. However, the unsimplified versions were used to calculate the marginal *R*^2^ values (for further details, see Supplementary Materials). The GRSS model indicated the marginal *R*^2^ = 0.018, and the SPS marginal *R*^2^ = 0.019.

### Additional Exploratory Analyses

**Fig. 4:**
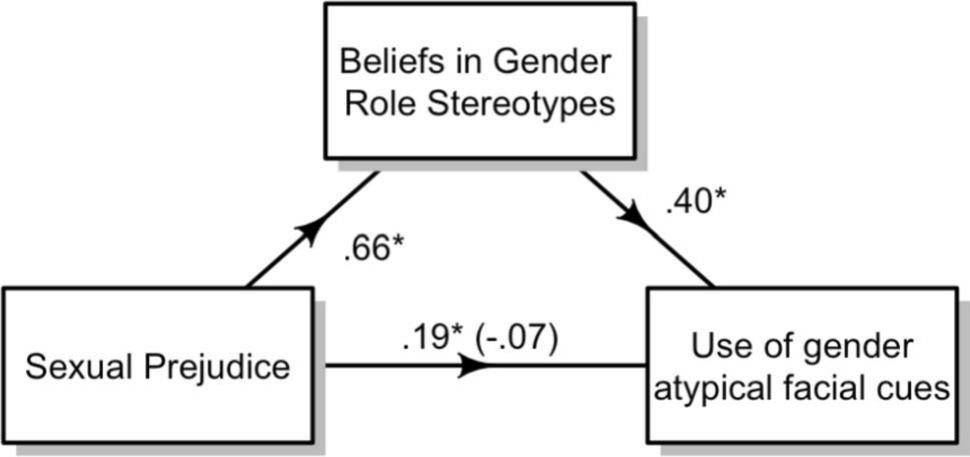
The mediating role of beliefs in gender role stereotypes in the relationship between sexual prejudice and use of gender atypical facial cues

At the request of a reviewer, we assess whether beliefs in gender role stereotypes mediates the association between sexual prejudice and the use of gender typicality cues when making sexual orientation judgements; this is potentially true given that the effect of sexual prejudice becomes non-significant when included in a model with beliefs in gender role stereotypes. In order to assess this, we first computed a score for each participant that represented their use of gender typicality cues; for this, we used the random slopes from a mixed effects model where gender typicality predicted sexual orientation judgement with participant as the grouping factor (i.e., participants who had a steeper slope between facial gender typicality and heterosexual judgements were deemed more likely to use gender typicality cues). A mediation via bootstrapping was conducted using the psych package in R (Revelle, 2024). There was a significant indirect effect of sexual prejudice on use of facial gender typicality cues through beliefs in gender role stereotypes (indirect effect = 0.26, 95% CI 0.13–0.42), while the direct effect was close to zero and non-significant (direct effect = − 0.07, *t*(216) = − 0.84, *p* = 0.400). This would indicate a full mediation (see Fig. 4).

Additional exploratory analyses were conducted to investigate whether participant gender or sexual orientation (heterosexual or non-heterosexual) influenced judgements of sexual orientation or interacted with face gender typicality. A brief summary of these results are reported here; for full model results, see the Supplementary Materials. Overall, there were no significant effects of participant gender on the use of perceived or objective gender typicality cues when making the sexual orientation judgements reported. However, a significant interaction was reported between participant gender and sexual prejudice score. As sexual prejudice scores increased, the likelihood of non-heterosexual being selected increased more for women than for men. For participant sexual orientation, a significant interaction was found between perceived face gender typicality and participant sexual orientation, such that heterosexual participants were more likely to use gender typicality cues when making judgements of sexual orientation compared to non-heterosexual participants. No other main or interaction effects of participant sexual orientation were significant.

## Discussion

Consistent with Study 1 and previous research, we replicated the significant effect of perceived face gender typicality on sexual orientation judgements (Bjornsdottir et al., 2022; Lick & Johnson, 2014, 2016; Stern et al., 2013). Interestingly, we found divergent effects between the gender typicality scores calculated via perceived masculinity/femininity and the objective shape score calculated via geometric morphometrics. In line with previous literature and Hypothesis 1, face shape gender typicality influenced sexual orientation judgements (Freeman et al., 2010). However, in contrast with our perceived gender typicality measure, the use of this objective cue was not influenced by participant belief in gender role stereotypes or sexual prejudice. A possible explanation for this finding is that the objective measure of gender typicality accounted only for face shape and did not consider other shape (e.g., nose length, forehead tilt, puckered mouth) or non-shape aspects that may influence gender typicality, for example, texture or complexion color. Indeed, previous research suggests that face shape sexual dimorphism may only account for a small amount of variance of subjective ratings of facial gender typicality (Hester et al., 2021; Komori et al., 2011). Therefore, participant individual differences in belief in gender role stereotypes and sexual prejudice may interact with cues of facial gender typicality beyond face shape.

Supporting Hypothesis 3, we found a significant effect of sexual prejudice on the use of gender typicality cues, with participants being more likely to judge gender typical faces as heterosexual and gender atypical faces as non-heterosexual, as their sexual prejudice increased. It may be that highly prejudiced individuals are more likely to rely on automatic judgements formed using stereotypes related to gender and sexual orientation (Cox et al., 2016; Devine, 1989; Stern et al., 2013). However, we note that this effect with sexual prejudice is non-significant once belief in gender stereotypes is included in the model. Indeed, additional exploratory analyses indicate that belief in gender stereotypes fully mediates this association with sexual prejudice. Since endorsement of traditional gender roles is associated with higher prejudice toward homosexuality (Whitley, 2001; Wilkinson, 2006), our findings begin to disentangle these two related attitudes. Regardless, our findings suggest that the use of gender typicality cues is specific to beliefs in gender stereotypes and not to attitudes toward sexual minorities more generally.

A strength of Study 2 is that a wider variety of faces were used compared to Study 1, both in terms of ethnicity and facial masculinity/femininity. Potentially to avoid the influence of racial stereotypes, much previous literature tends to only include White faces (e.g., Cox et al., 2016; Lick & Johnson, 2014, 2016; Stern et al., 2013), or do not report target face race (e.g., Freeman et al., 2010; Rule & Ambady, 2008; Rule et al., 2009). However, our results suggest that the influence of gender stereotypes on use of facial gender typicality on sexual orientation judgements is not specific to White faces, nor limited to extremely masculine/feminine faces.

## General Discussion

Across two studies, we investigated the effect of participant belief in gender stereotypes and target facial gender typicality when making sexual orientation judgements. Supporting Hypothesis 1, we found that gender typicality influenced overall likelihood of judging a face as heterosexual. We also found support for Hypothesis 2, where this effect was moderated by participant’s beliefs in gender stereotypes, such that those with greater belief in gender stereotypes were more likely to use gender typicality cues when making sexual orientation judgements. Although the effect sizes were small, these results highlight the importance of considering individual differences in stereotyping and their impact on sexual orientation judgements.

Results from both studies adds to the growing literature that cues of gender typicality are used when making judgements of sexual orientation. As facial gender typicality increased, the face was more likely to be judged as heterosexual compared to non-heterosexual (Bjornsdottir et al., 2022; Blashill & Powlishta, 2009; Kite & Deaux, 1987; Lick & Johnson, 2014, 2016; Stern et al., 2013). Given that the current studies differ in methodology compared to previous work (e.g., in the variety of ethnicities presented, how gender typicality is operationalised, the scale participants responded on, and the number of stimuli included), this would suggest that the use of gender typicality when making sexual orientation judgements is a robust effect.

Consistent with Hypothesis 2, both studies report that participants with greater beliefs in gender role stereotypes were more likely to use perceived gender typicality cues when making judgements of sexual orientation. Our results add to literature that individual differences in perceivers can influence sexual orientation judgements (Ambady et al., 1999; Cox et al., 2016; Johnson & Ghavami, 2011; Stern et al., 2013). Results from Study 2 also suggest that these effects are specific to beliefs in gender stereotypes, and not explained by sexual prejudice more generally (on the contrary, any apparent association between sexual prejudice and use of gender typicality cues was fully mediated by beliefs in gender stereotypes). A possible mechanism for this moderating effect is that participants are able to revise automatic appraisals based on stereotypes according to their own beliefs. Due to the speed at which sexual orientation judgements can be made from faces (e.g., Rule & Ambady, 2008; Rule et al., 2009), it is likely that judgements are made automatically according to cultural stereotypes; indeed, this appears to be somewhat supported in our studies by the significant main effect of gender typicality on sexual orientation judgements. Previous research demonstrates that individuals who have weaker belief in stereotypes may be less likely to use them when making judgements (Carter et al., 2006; Cox et al., 2016), particularly when allowed the time and mental capacity to inhibit initial judgements (Stern et al., 2013). Given that participants in our studies had unlimited time in which to view the faces and make their response, this allows ample opportunity to revise their initial judgements according to their own beliefs in gender roles. To further explore the role of individual differences in beliefs in gender stereotypes, future research could limit the time participants had, or cognitively load participants when making sexual orientation judgements.

In the current study, participants rated faces as either heterosexual or non-heterosexual; therefore, some nuance of sexual orientation judgements may have been lost. Methodological differences have been shown to influence sexual orientation judgements. For example, some previous literature reports gay and bisexual faces as being indistinguishable (Ding & Rule, 2012). However, when asked to categorize faces as either “bisexual” or “not bisexual”, observers were able to categorize bisexual faces separately from gay faces (Lick et al., 2015). Moreover, research demonstrates that sexual attraction (as a way of defining sexual orientation on a continuous scale) can be judged from faces (Bjornsdottir et al., 2022). Future research may expand upon the current study by considering how individual differences in participant stereotyping may influence judgements of sexual orientation on a continuous scale, or within non-heterosexual orientation categories. Although the current study was conducted with online participants, previous literature demonstrates good data quality of participants recruited through online platforms (e.g., Douglas et al., 2023), and very little difference in data quality between lab-based and online participants (e.g., Clifford & Jerit, 2014).

In conclusion, it has been well established that cues of gender typicality are used when making sexual orientation judgements (Blashill & Powlishta, 2009; Kite & Deaux, 1987). The current studies extend this research by considering participant individual differences. Belief in stereotypes (Carter et al., 2006), and prejudice toward a specific group (Devine, 1989), has been shown to influence the use of associated stereotypes when making social judgements. The current research provides support for the notion that individual differences in gender role stereotyping influences the use of facial gender typicality cues when making sexual orientation judgements. Namely, that as facial gender typicality increases, participants with greater belief in gender role stereotypes are more likely to give a heterosexual judgement. This finding highlights the importance of considering individual differences when researching how sexual orientation judgements are formed.

## Supplementary Information

Below is the link to the electronic supplementary material.

Supplementary file1 (PDF 195 KB)

Supplementary file2 (PDF 344 KB)

## Data Availability

Data are available on the Open Science Framework at osf.io/njf3p/.

## Code Availability

Analysis code is available on the Open Science Framework at osf.io/njf3p/.

## References

[CR1] Ambady, N., Hallahan, M., & Conner, B. (1999). Accuracy of judgments of sexual orientation from thin slices of behavior. *Journal of Personality and Social Psychology,**77*(3), 538–547. 10.1037/0022-3514.77.3.538 10510507 10.1037//0022-3514.77.3.538

[CR2] Bailey, J. M., & Zucker, K. J. (1995). Childhood sex-typed behavior and sexual orientation: A conceptual analysis and quantitative review. *Developmental Psychology,**31*(1), 43–55. 10.1037/0012-1649.31.1.43

[CR3] Barr, D. J. (2013). Random effects structure for testing interactions in linear mixed-effects models. *Frontiers in Psychology*, *4*. 10.3389/fpsyg.2013.00328 10.3389/fpsyg.2013.00328 PMC3672519 23761778

[CR4] Barr, D. J., Levy, R., Scheepers, C., & Tily, H. J. (2013). Random effects structure for confirmatory hypothesis testing: Keep it maximal. *Journal of Memory and Language,**68*(3), 255–278. 10.1016/j.jml.2012.11.001 10.1016/j.jml.2012.11.001 PMC3881361 24403724

[CR5] Bates, D., Mächler, M., Bolker, B., & Walker, S. (2015). Fitting linear mixed-effects models using lme4. *Journal of Statistical Software,**67*(1), 1–48. 10.18637/jss.v067.i01

[CR6] Bjornsdottir, R. T., Cheso, D., & Rule, N. O. (2022). Beyond categories: Perceiving sexual attraction from faces. *British Journal of Psychology,**113*(1), 226–247. 10.1111/bjop.12523 34296765 10.1111/bjop.12523

[CR7] Bjornsdottir, R. T., & Rule, N. O. (2020). Emotion and gender typicality cue sexual orientation differently in women and men. *Archives of Sexual Behavior,**49*(7), 2547–2560. 10.1007/s10508-020-01700-3 32394110 10.1007/s10508-020-01700-3 PMC7497461

[CR8] Blashill, A. J., & Powlishta, K. K. (2009). Gay stereotypes: The use of sexual orientation as a cue for gender-related attributes. *Sex Roles,**61*, 783–793. 10.1007/s11199-009-9684-7

[CR9] Carter, J. D., Hall, J. A., Carney, D. R., & Rosip, J. C. (2006). Individual differences in the acceptance of stereotyping. *Journal of Research in Personality,**40*(6), 1103–1118. 10.1016/j.jrp.2005.11.005

[CR10] Chonody, J. M. (2013). Measuring sexual prejudice against gay men and lesbian women: Development of the sexual prejudice scale (SPS). *Journal of Homosexuality,**60*(6), 895–926. 10.1080/00918369.2013.774863 23688314 10.1080/00918369.2013.774863

[CR11] Clifford, S., & Jerit, J. (2014). Is there a cost to convenience? An experimental comparison of data quality in laboratory and online studies. *Journal of Experimental Political Science,**1*(2), 120–131. 10.1017/xps.2014.5

[CR13] Cox, W. T., Devine, P. G., Bischmann, A. A., & Hyde, J. S. (2016). Inferences about sexual orientation: Roles of stereotypes, faces, and the gaydar myth. *Journal of Sex Research,**53*(2), 157–171. 10.1080/00224499.2015.1015714 26219212 10.1080/00224499.2015.1015714 PMC4731319

[CR14] DeBruine, L. M., & Barr, D. J. (2021). Understanding mixed-effects models through data simulation. *Advances in Methods and Practices in Psychological Science,**4*(1). 10.1177/2515245920965119

[CR15] Devine, P. G. (1989). Stereotypes and prejudice: Their automatic and controlled components. *Journal of Personality and Social Psychology,**56*(1), 5–18. 10.1037/0022-3514.56.1.5

[CR16] Ding, J. Y., & Rule, N. O. (2012). Gay, straight, or somewhere in between: Accuracy and bias in the perception of bisexual faces. *Journal of Nonverbal Behavior,**36*, 165–176. 10.1007/s10919-011-0129-y

[CR17] Dong, J., Leger, K., Shiramizu, V. K., Marcinkowska, U. M., Lee, A. J., & Jones, B. C. (2023). The importance of face-shape masculinity for perceptions of male dominance depends on study design. *Scientific Reports,**13*(1), 12620. 10.1038/s41598-023-39912-x 37537340 10.1038/s41598-023-39912-x PMC10400540

[CR18] Douglas, B. D., Ewell, P. J., & Brauer, M. (2023). Data quality in online human-subjects research: Comparisons between MTurk, Prolific, CloudResearch, Qualtrics, and SONA. *PLoS ONE,**18*(3), e0279720. 10.1371/journal.pone.0279720 36917576 10.1371/journal.pone.0279720 PMC10013894

[CR19] Freeman, J. B., Johnson, K. L., Ambady, N., & Rule, N. O. (2010). Sexual orientation perception involves gendered facial cues. *Personality and Social Psychology Bulletin,**36*(10), 1318–1331. 10.1177/0146167210378755 20682754 10.1177/0146167210378755

[CR20] Gaunt, R. (2013). Breadwinning moms, caregiving dads: Double standard in social judgments of gender norm violators. *Journal of Family Issues,**34*(1), 3–24. 10.1177/0192513X12438686

[CR21] González-Álvarez, J. (2017). Perception of sexual orientation from facial structure: A study with artificial face models. *Archives of Sexual Behavior,**46*(5), 1251–1260. 10.1007/s10508-016-0929-6 28155008 10.1007/s10508-016-0929-6

[CR22] González-Álvarez, J., & Sos-Peña, R. (2022). Facial structure and perception of sexual orientation: Research with face models based on photographs of real people. *International Journal of Psychology,**57*(2), 279–288. 10.1002/ijop.12811 34562272 10.1002/ijop.12811

[CR23] Hester, N., Jones, B. C., & Hehman, E. (2021). Perceived femininity and masculinity contribute independently to facial impressions. *Journal of Experimental Psychology: General,**150*(6), 1147–1154. 10.1037/xge0000989 33119352 10.1037/xge0000989

[CR24] Holzleitner, I. J., & DeBruine, L. M. (2021). *Calculate sexual dimorphism of faces*. GitHub. https://iholzleitner.github.io/facefuns/articles/calcSD.html.

[CR25] Johnson, K. L., & Ghavami, N. (2011). At the crossroads of conspicuous and concealable: What race categories communicate about sexual orientation. *PLoS ONE,**6*(3), e18025. 10.1371/journal.pone.0018025 21483863 10.1371/journal.pone.0018025 PMC3069043

[CR26] Johnson, K. L., Gill, S., Reichman, V., & Tassinary, L. G. (2007). Swagger, sway, and sexuality: Judging sexual orientation from body motion and morphology. *Journal of Personality and Social Psychology,**93*(3), 321–334. 10.1037/0022-3514.93.3.321.supp 17723051 10.1037/0022-3514.93.3.321

[CR27] Kite, M. E., & Deaux, K. (1987). Gender belief systems: Homosexuality and the implicit inversion theory. *Psychology of Women Quarterly,**11*(1), 83–96. 10.1111/j.1471-6402.1987.tb00776

[CR28] Komori, M., Kawamura, S., & Ishihara, S. (2011). Multiple mechanisms in the perception of face gender: Effect of sex-irrelevant features. *Journal of Experimental Psychology: Human Perception and Performance,**37*(3), 626–633. 10.1037/a0020369 20822297 10.1037/a0020369

[CR29] Kuznetsova, A., Brockhoff, P. B., & Christensen, R. H. B. (2017). lmerTest package: Tests in linear mixed effects models. *Journal of Statistical Software,**82*(13), 1–26. 10.18637/jss.v082.i13

[CR30] Leger, K., Dong, J., DeBruine, L. M., Jones, B. C., & Shiramizu, V. K. (2023). Assessing the roles of shape prototypicality and sexual dimorphism in ratings of the trustworthiness of faces. *Scientific Reports,**13*(1), 15662. 10.1038/s41598-023-42990-6 37731069 10.1038/s41598-023-42990-6 PMC10511419

[CR31] Lehavot, K., & Lambert, A. J. (2007). Toward a greater understanding of antigay prejudice: On the role of sexual orientation and gender role violation. *Basic and Applied Social Psychology,**29*(3), 279–292. 10.1080/01973530701503390

[CR32] Li, G., Kung, K. T., & Hines, M. (2017). Childhood gender-typed behavior and adolescent sexual orientation: A longitudinal population-based study. *Developmental Psychology,**53*(4), 764–777. 10.1037/dev0000281 28221049 10.1037/dev0000281

[CR33] Lick, D. J., & Johnson, K. L. (2014). Perceptual underpinnings of antigay prejudice: Negative evaluations of sexual minority women arise on the basis of gendered facial features. *Personality and Social Psychology Bulletin,**40*(9), 1178–1192. 10.1177/0146167214538288 24914011 10.1177/0146167214538288

[CR34] Lick, D. J., & Johnson, K. L. (2016). Straight until proven gay: A systematic bias toward straight categorizations in sexual orientation judgments. *Journal of Personality and Social Psychology,**110*(6), 801–817. 10.1037/pspa0000052 27281352 10.1037/pspa0000052

[CR35] Lick, D. J., Johnson, K. L., & Rule, N. O. (2015). Disfluent processing of nonverbal cues helps to explain anti-bisexual prejudice. *Journal of Nonverbal Behavior,**39*, 275–288. 10.1007/s10919-015-0211-y

[CR36] Ma, D. S., Correll, J., & Wittenbrink, B. (2015). The Chicago face database: A free stimulus set of faces and norming data. *Behavior Research Methods,**47*, 1122–1135. 10.3758/s13428-014-0532-5 25582810 10.3758/s13428-014-0532-5

[CR37] Macrae, C. N., & Bodenhausen, G. V. (2000). Social cognition: Thinking categorically about others. *Annual Review of Psychology,**51*(1), 93–120. 10.1146/annurev.psych.51.1.93 10751966 10.1146/annurev.psych.51.1.93

[CR38] Madon, S. (1997). What do people believe about gay males? A study of stereotype content and strength. *Sex Roles,**37*(9), 663–685. 10.1007/BF02936334

[CR39] Mills, M. J., Culbertson, S. S., Huffman, A. H., & Connell, A. R. (2012). Assessing gender biases: Development and initial validation of the gender role stereotypes scale. *Gender in Management: An International Journal,**27*(8), 520–540. 10.1108/17542411211279715

[CR12] R Core Team. (2024). *R: A language and environment for statistical computing*. R Foundation for Statistical Computing, Vienna, Austria. http://www.R-project.org/.

[CR40] Revelle, W. (2024). psych: Procedures for psychological, psychometric, and personality research. Northwestern University, Evanston, Illinois. *R package version 2.4.6,* https://CRAN.R-project.org/package=psych.(9).

[CR41] Rieger, G., Linsenmeier, J. A., Gygax, L., & Bailey, J. M. (2008). Sexual orientation and childhood gender nonconformity: Evidence from home videos. *Developmental Psychology,**44*(1), 46–58. 10.1037/0012-1649.44.1.46 18194004 10.1037/0012-1649.44.1.46

[CR42] Rieger, G., Linsenmeier, J. A., Gygax, L., Garcia, S., & Bailey, J. M. (2010). Dissecting “gaydar”: Accuracy and the role of masculinity–femininity. *Archives of Sexual Behavior,**39*, 124–140. 10.1007/s10508-008-9405-2 18810629 10.1007/s10508-008-9405-2

[CR43] Rule, N. O. (2017). Perceptions of sexual orientation from minimal cues. *Archives of Sexual Behavior,**46*(1), 129–139. 10.1007/s10508-016-0779-2 27527876 10.1007/s10508-016-0779-2

[CR44] Rule, N. O., & Ambady, N. (2008). Brief exposures: Male sexual orientation is accurately perceived at 50 ms. *Journal of Experimental Social Psychology,**44*(4), 1100–1105. 10.1016/j.jesp.2007.12.001

[CR45] Rule, N. O., Ambady, N., Adams, R. B., & Macrae, C. N. (2007). Us and them: Memory advantages in perceptually ambiguous groups. *Psychonomic Bulletin and Review,**14*(4), 687–692. 10.3758/BF03196822 17972734 10.3758/bf03196822

[CR46] Rule, N. O., Ambady, N., & Hallett, K. C. (2009). Female sexual orientation is perceived accurately, rapidly, and automatically from the face and its features. *Journal of Experimental Social Psychology,**45*(6), 1245–1251. 10.1016/j.jesp.2009.07.010

[CR47] Rule, N. O., Ishii, K., Ambady, N., Rosen, K. S., & Hallett, K. C. (2011). Found in translation: Cross-cultural consensus in the accurate categorization of male sexual orientation. *Personality and Social Psychology Bulletin,**37*(11), 1499–1507. 10.1177/0146167211415630 21807952 10.1177/0146167211415630

[CR48] Singh, B., Gambrell, A., & Correll, J. (2023). Face templates for the Chicago face database. *Behavior Research Methods,**55*(2), 639–645. 10.31234/osf.io/f56a4 35396615 10.3758/s13428-022-01830-7

[CR49] Skorska, M. N., Geniole, S. N., Vrysen, B. M., McCormick, C. M., & Bogaert, A. F. (2015). Facial structure predicts sexual orientation in both men and women. *Archives of Sexual Behavior,**44*, 1377–1394. 10.1007/s10508-014-0454-4 25550146 10.1007/s10508-014-0454-4

[CR50] Stern, C., West, T. V., Jost, J. T., & Rule, N. O. (2013). The politics of gaydar: Ideological differences in the use of gendered cues in categorizing sexual orientation. *Journal of Personality and Social Psychology,**104*(3), 520–541. 10.1037/a0031187 23276275 10.1037/a0031187

[CR51] Watts, T. M., Holmes, L., Raines, J., Orbell, S., & Rieger, G. (2018). Gender nonconformity of identical twins with discordant sexual orientations: Evidence from childhood photographs. *Developmental Psychology,**54*(4), 788–801. 10.1037/dev0000461 29154643 10.1037/dev0000461

[CR52] Whitley, B. E. (2001). Gender-role variables and attitudes toward homosexuality. *Sex Roles,**45*, 691–721. 10.1023/A:1015640318045

[CR53] Wilkinson, W. W. (2006). Exploring heterosexual women’s anti-lesbian attitudes. *Journal of Homosexuality,**51*(2), 139–155. 10.1300/J082v51n02_08 16901871 10.1300/J082v51n02_08

[CR54] Xu, Y., Norton, S., & Rahman, Q. (2019). Early life conditions and adolescent sexual orientation: A prospective birth cohort study. *Developmental Psychology,**55*(6), 1226–1243. 10.1037/dev0000704 30762413 10.1037/dev0000704

[CR55] Zelditch, M., Swiderski, D., & Sheets, H. D. (2012). *Geometric morphometrics for biologists: A primer*. Elsevier Academic Press.

