# Supplementary material for: Belief in Gender Role Stereotypes Moderates the Use of Gender Typicality Cues when Making Sexual Orientation Judgements from Faces
Source: Arch Sex Behav. 2024 Dec 14;54(3):1233–44. doi: 10.1007/s10508-024-03046-6 (PMC11926015; doi:10.1007/s10508-024-03046-6)
Supplement: Supplementary file 1 — Supplementary file1 (PDF 195 KB) [file 10508_2024_3046_MOESM1_ESM.pdf]

Gender Role Stereotypes and Sexual Orientation Judgements - Study 1

Load Packages

```
library(tidyverse)
library(lme4)
library(lmerTest)
library(gghalves)
```

Load Data

```
complete.data <- read.csv("Fulldata_Study1.csv")
```

Prepare Dataset for Analysis

Higher scores on the GRSS indicate greater beliefs in Gender Role Stereotypes.

Participant Dataset

Calculate GRSS and remove participants with missing data on key variables.

```
participant.data <- complete.data %>%
  mutate(GRSS_male = 24 - (GRSS_1 + GRSS_3 + GRSS_4 + GRSS_8) ,
          GRSS_female = (GRSS_2 + GRSS_5 + GRSS_6 + GRSS_7),
         GRSS_total = GRSS_male + GRSS_female,
          z.GRSS_male = c(scale(GRSS_male)),
          z.GRSS_female = c(scale(GRSS_female)),
         z.GRSS_total = c(scale(GRSS_total))) %>%
  select(ResponseId,sex,age,so,GRSS_male,GRSS_female,GRSS_total,z.GRSS_male,
         z.GRSS_female,z.GRSS_total,FM1:MW20) %>%
  filter(!is.na(GRSS_total))
```

Rating Dataset

Faces are either in the "Masculine" or "Feminine" group, which were the top scoring faces on perceived Masculinity and Femininity for each sex according to the norming data.

Face Gender Inversion: Faces coded as .5 fall in either the Feminine Male or Masculine Female groups (i.e., faces are stereo-atypical (inverted) for their sex). Faces coded -.5 fall into either the Feminine Female or Masculine Male groups (i.e., faces are stereotypical for their sex).

Choice: Participants who rated face as 'Heterosexual' coded as 0. Participants who rated face as 'Non-heterosexual' coded as 1.

```
analysis.data <- participant.data %>%
  gather(key = "face.id",value = "choice",FM1:MW20) %>%
  filter(!is.na(choice)) %>%
  mutate(face.type = ifelse(substr(face.id,1,1) == "F","Feminine","Masculine"),
         face.sex = ifelse(substr(face.id,2,2) == "M","Male","Female"),
         choice = recode(choice,`1` = 0,`2` = 1),
         face.gender.typicality = ifelse((face.type == "Masculine" & face.sex == "Female") |
                                         (face.type == "Feminine" & face.sex == "Male"),-.5,.5),
         e.face.type = ifelse(face.type == "Masculine",.5,-5),
         e.face.sex = ifelse(face.sex == "Male",.5,-.5))
```

Sample Descriptive Statistics

Participant Gender: 1 = Female 2 = Male 3 = Non-Binary 4 = Other 5 = Prefer not to say

```
table(participant.data$sex)
```

```
##
##   1   2   3   4   5
## 195  74  10   2   2
```

```
summarise(participant.data,
          mean.age = mean(age,na.rm = TRUE),
          sd.age = sd(age,na.rm = TRUE))
```

```
##   mean.age   sd.age
## 1 21.97091 6.388792
```

Participant Sexual Orientation 1 = Heterosexual 2 = Homosexual 3 = Bisexual/Pansexual 4 = Other 5 = Prefer not to say

```
table(participant.data$so)
```

```
##
##   1   2   3   4   5
## 194  20  58   7   4
```

Main Analysis

Full Sample

```r
model <- glmer(choice ~ z.GRSS_total*face.gender.typicality +
                 (1 + face.gender.typicality || ResponseId) +
                 (1 + z.GRSS_total || face.id),data = analysis.data,family = "binomial")
summary(model)
```

```
## Generalized linear mixed model fit by maximum likelihood (Laplace
##   Approximation) [glmerMod]
##  Family: binomial  ( logit )
## Formula:
## choice ~ z.GRSS_total * face.gender.typicality + (1 + face.gender.typicality ||
##     ResponseId) + (1 + z.GRSS_total || face.id)
##    Data: analysis.data
##
##      AIC      BIC   logLik deviance df.resid
##  26006.1  26070.2 -12995.0  25990.1    22460
##
## Scaled residuals:
##     Min      1Q  Median      3Q     Max
## -3.0050 -0.6858 -0.4316  0.8950  4.8460
##
## Random effects:
##  Groups       Name                   Variance Std.Dev.
##  ResponseId   (Intercept)            0.60051  0.7749
##  ResponseId.1 face.gender.typicality 0.54312  0.7370
##  face.id      (Intercept)            0.19207  0.4383
##  face.id.1    z.GRSS_total           0.01423  0.1193
## Number of obs: 22468, groups:  ResponseId, 282; face.id, 80
##
## Fixed effects:
##                                     Estimate Std. Error z value Pr(>|z|)
## (Intercept)                         -0.84240    0.06943 -12.134  < 2e-16 ***
## z.GRSS_total                        -0.04103    0.05089  -0.806     0.42
## face.gender.typicality              -0.80717    0.11218  -7.195 6.23e-13 ***
## z.GRSS_total:face.gender.typicality -0.31162    0.06093  -5.114 3.15e-07 ***
## ---
## Signif. codes:  0 '***' 0.001 '**' 0.01 '*' 0.05 '.' 0.1 ' ' 1
##
## Correlation of Fixed Effects:
##             (Intr) z.GRSS_t fc.gn.
## z.GRSS_totl 0.006
## fc.gndr.typ 0.011  0.005
## z.GRSS_t:.. 0.007  0.028    0.008
```

```r
plot.data <- analysis.data %>%
  group_by(ResponseId,face.type,face.sex,face.gender.typicality,GRSS_total,z.GRSS_total) %>%
  summarise(mean.choice = mean(choice)) %>%
  mutate(cat.GRSS = ifelse(z.GRSS_total < 0, "Low","High"),
         cat.GRSS = factor(cat.GRSS,levels = c("Low","High")),
         face.gender.typicality = ifelse(face.gender.typicality == -.5,"Gender Atypical","Gender Typical
         face.sex = ifelse(face.sex == "Female","Female Faces","Male Faces")) %>%
  ungroup()
```

```
## `summarise()` has grouped output by 'ResponseId', 'face.type', 'face.sex',
```

```
## 'face.gender.typicality', 'GRSS_total'. You can override using the `.groups`
## argument.
```

```
ggplot(plot.data,aes(x = cat.GRSS,y =mean.choice,
                     fill = face.gender.typicality)) +
  geom_half_violin(data = filter(plot.data,face.gender.typicality == "Gender Atypical"),side = "l") +
    geom_half_violin(data = filter(plot.data,face.gender.typicality == "Gender Typical"),side = "r") +
  stat_summary(data = filter(plot.data,face.gender.typicality == "Gender Atypical"),position = position_
    stat_summary(data = filter(plot.data,face.gender.typicality == "Gender Typical"),position = positior
  theme_classic() +
  xlab("Beliefs in Gender Role Stereotypes") +
  ylab("Proportion of Faces Judged as Non-Heterosexual") +
  labs(fill = "Gender Typicality of Face") +
  theme(legend.position = "bottom") +
  facet_wrap(~ face.sex)
```

```
## No summary function supplied, defaulting to `mean_se()`
## No summary function supplied, defaulting to `mean_se()`
## No summary function supplied, defaulting to `mean_se()`
## No summary function supplied, defaulting to `mean_se()`
```

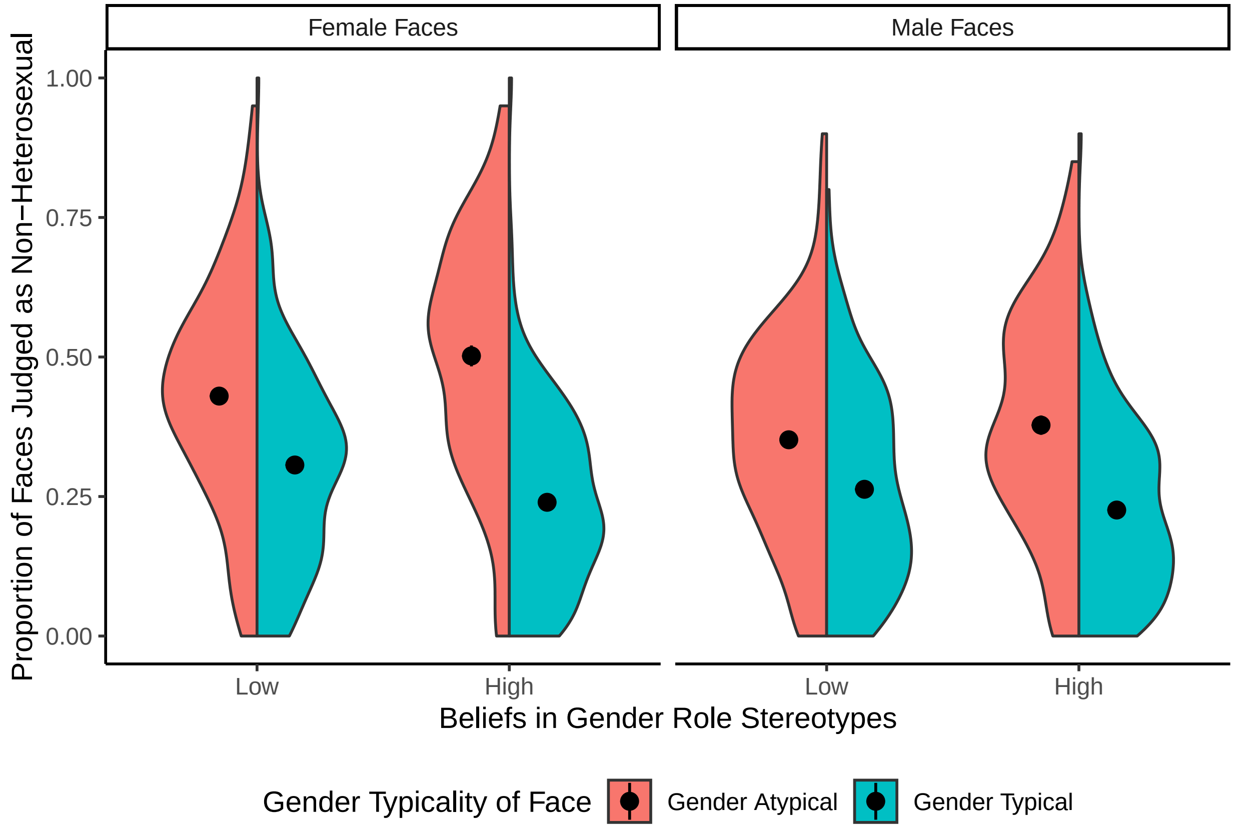


```
ggsave("Figure 1.png")
```

```
## Saving 6.5 x 4.5 in image
## No summary function supplied, defaulting to `mean_se()`
```

```
## No summary function supplied, defaulting to `mean_se()`
## No summary function supplied, defaulting to `mean_se()`
## No summary function supplied, defaulting to `mean_se()`
```

Male Faces

```
model <- glmer(choice ~ z.GRSS_total*face.gender.typicality +
                 (1 + face.gender.typicality || ResponseId) +
                 (1 + z.GRSS_total || face.id),data = filter(analysis.data,face.sex == "Male"),family =
summary(model)
```

```
## Generalized linear mixed model fit by maximum likelihood (Laplace
##   Approximation) [glmerMod]
##  Family: binomial  ( logit )
## Formula:
## choice ~ z.GRSS_total * face.gender.typicality + (1 + face.gender.typicality ||
##     ResponseId) + (1 + z.GRSS_total || face.id)
##    Data: filter(analysis.data, face.sex == "Male")
##
##      AIC      BIC   logLik deviance df.resid
##  12690.2  12748.8  -6337.1  12674.2    11208
##
## Scaled residuals:
##     Min      1Q  Median      3Q     Max
## -2.2313 -0.6507 -0.4121  0.8941  4.7019
##
## Random effects:
##  Groups       Name                   Variance Std.Dev.
##  ResponseId   (Intercept)            0.67293  0.8203
##  ResponseId.1 face.gender.typicality 0.62348  0.7896
##  face.id      (Intercept)            0.17172  0.4144
##  face.id.1    z.GRSS_total           0.01933  0.1390
## Number of obs: 11216, groups:  ResponseId, 281; face.id, 40
##
## Fixed effects:
##                                     Estimate Std. Error z value Pr(>|z|)
## (Intercept)                         -1.02692    0.08551 -12.009  < 2e-16 ***
## z.GRSS_total                        -0.05589    0.05873  -0.952    0.341
## face.gender.typicality              -0.69142    0.14695  -4.705 2.54e-06 ***
## z.GRSS_total:face.gender.typicality -0.24644    0.07973  -3.091    0.002 **
## ---
## Signif. codes:  0 '***' 0.001 '**' 0.01 '*' 0.05 '.' 0.1 ' ' 1
##
## Correlation of Fixed Effects:
##             (Intr) z.GRSS_t fc.gn.
## z.GRSS_totl 0.005
## fc.gndr.typ 0.016  0.007
## z.GRSS_t:.. 0.010  0.040    0.009
```

Female Faces

```
model <- glmer(choice ~ z.GRSS_total*face.gender.typicality +
                 (1 + face.gender.typicality || ResponseId) +
                 (1 + z.GRSS_total || face.id),data = filter(analysis.data,face.sex == "Female"),family
summary(model)
```

```
## Generalized linear mixed model fit by maximum likelihood (Laplace
##   Approximation) [glmerMod]
##  Family: binomial  ( logit )
## Formula:
## choice ~ z.GRSS_total * face.gender.typicality + (1 + face.gender.typicality ||
##     ResponseId) + (1 + z.GRSS_total || face.id)
##    Data: filter(analysis.data, face.sex == "Female")
##
##      AIC      BIC   logLik deviance df.resid
##  13418.9  13477.5  -6701.4  13402.9    11244
##
## Scaled residuals:
##     Min      1Q  Median      3Q     Max
## -2.8710 -0.7000 -0.4248  0.8514  3.6333
##
## Random effects:
##  Groups       Name                   Variance Std.Dev.
##  ResponseId   (Intercept)            0.55102  0.74231
##  ResponseId.1 face.gender.typicality 0.93539  0.96716
##  face.id      (Intercept)            0.15614  0.39514
##  face.id.1    z.GRSS_total           0.00713  0.08444
## Number of obs: 11252, groups:  ResponseId, 282; face.id, 40
##
## Fixed effects:
##                                     Estimate Std. Error z value Pr(>|z|)
## (Intercept)                         -0.67028    0.07992  -8.387  < 2e-16 ***
## z.GRSS_total                        -0.02855    0.05159  -0.553     0.58
## face.gender.typicality              -0.96166    0.14479  -6.642 3.10e-11 ***
## z.GRSS_total:face.gender.typicality -0.40420    0.07829  -5.163 2.43e-07 ***
## ---
## Signif. codes:  0 '***' 0.001 '**' 0.01 '*' 0.05 '.' 0.1 ' ' 1
##
## Correlation of Fixed Effects:
##             (Intr) z.GRSS_t fc.gn.
## z.GRSS_totl 0.009
## fc.gndr.typ 0.013  0.008
## z.GRSS_t:.. 0.011  0.037    0.013
```
