# Supplementary material for: Belief in Gender Role Stereotypes Moderates the Use of Gender Typicality Cues when Making Sexual Orientation Judgements from Faces
Source: Arch Sex Behav. 2024 Dec 14;54(3):1233–44. doi: 10.1007/s10508-024-03046-6 (PMC11926015; doi:10.1007/s10508-024-03046-6)
Supplement: Supplementary file 2 — Supplementary file2 (PDF 344 KB) [file 10508_2024_3046_MOESM2_ESM.pdf]

Gender Role Stereotypes and Sexual Orientation Judgements - Study 2

Load packages

```r
library(tidyverse)
```

```
## -- Attaching core tidyverse packages ------------------------ tidyverse 2.0.0 --
## v dplyr     1.1.4     v readr     2.1.5
## v forcats   1.0.0     v stringr   1.5.1
## v ggplot2   3.5.0     v tibble    3.2.1
## v lubridate 1.9.3     v tidyr     1.3.1
## v purrr     1.0.2
## -- Conflicts ------------------------------------------ tidyverse_conflicts() --
## x dplyr::filter() masks stats::filter()
## x dplyr::lag()    masks stats::lag()
## i Use the conflicted package (<http://conflicted.r-lib.org/>) to force all conflicts to become errors
```

```r
library(psych)
```

```
##
## Attaching package: 'psych'
##
## The following objects are masked from 'package:ggplot2':
##
##     %+%, alpha
```

```r
library(lme4)
```

```
## Loading required package: Matrix
##
## Attaching package: 'Matrix'
##
## The following objects are masked from 'package:tidyr':
##
##     expand, pack, unpack
```

```r
library(lmerTest)
```

```
##
## Attaching package: 'lmerTest'
##
```

```
## The following object is masked from 'package:lme4':
##
##     lmer
##
## The following object is masked from 'package:stats':
##
##     step
```

Data Preparation

Read data

```
data <- read.csv("Fulldata_Study2.csv")

face.data <- read.csv("face.data.csv") %>%
  rename(image = Target)
```

Participant data

Calculate the GRSS and SPS scores. Higher scores on the GRSS indicate greater belief in gender role stereotypes, higher scores on the SPS indicate greater sexual prejudice.

```
participant.data <- data %>%
  mutate(g.SPS16R = recode(g.SPS16, "1"=6,"2"=5,"3"=4,"4"=3,"5"=2,"6"=1),
         g.SPS19R = recode(g.SPS19, "1"=6,"2"=5,"3"=4,"4"=3,"5"=2,"6"=1),
         g.SPS20R = recode(g.SPS20, "1"=6,"2"=5,"3"=4,"4"=3,"5"=2,"6"=1),
         g.SPS21R = recode(g.SPS21, "1"=6,"2"=5,"3"=4,"4"=3,"5"=2,"6"=1),
         g.SPS23R = recode(g.SPS23, "1"=6,"2"=5,"3"=4,"4"=3,"5"=2,"6"=1),
         l.SPS17R = recode(l.SPS17, "1"=6,"2"=5,"3"=4,"4"=3,"5"=2,"6"=1),
         l.SPS18R = recode(l.SPS18, "1"=6,"2"=5,"3"=4,"4"=3,"5"=2,"6"=1),
         l.SPS19R = recode(l.SPS19, "1"=6,"2"=5,"3"=4,"4"=3,"5"=2,"6"=1),
         l.SPS20R = recode(l.SPS20, "1"=6,"2"=5,"3"=4,"4"=3,"5"=2,"6"=1),
         l.SPS23R = recode(l.SPS23, "1"=6,"2"=5,"3"=4,"4"=3,"5"=2,"6"=1),
         gm.stereotype = g.SPS4+g.SPS6+g.SPS8+g.SPS11+g.SPS13,
         gm.affectval = g.SPS2+g.SPS3+g.SPS5+g.SPS7+g.SPS9+g.SPS16R,
         gm.equality = g.SPS19R+g.SPS20R+g.SPS21R+g.SPS23R,
         gm.global = gm.stereotype+gm.affectval+gm.equality,
         lw.stereotype = l.SPS1+l.SPS3+l.SPS5+l.SPS7+l.SPS11,
         lw.affectval = l.SPS4+l.SPS9+l.SPS10+l.SPS15+l.SPS17R+l.SPS19R,
         lw.equality = l.SPS8+l.SPS18R+l.SPS20R+l.SPS23R,
         lw.global = lw.stereotype+lw.affectval+lw.equality,
         GRSS.male = 24 - (GRSS1 + GRSS3 + GRSS4 + GRSS8),
         GRSS.female = GRSS2 + GRSS5 + GRSS6 + GRSS7,
         GRSS = GRSS.male+GRSS.female,
         z.GRSS = c(scale(GRSS)),
         z.lw.global = c(scale(lw.global)),
         z.gm.global = c(scale(gm.global)),
         z.sps = c(scale(lw.global+gm.global)),
         e.gender = case_when(gender == 1 ~ 0.5, gender == 2 ~ -0.5, TRUE ~ NA_real_), # men = .5, wome
```

```
        e.so = case_when(so == 1 ~ 0.5, so %in% c(2, 3, 4) ~ -0.5, TRUE ~ NA_real_)) %>%
  select(gorilla.id,age,gender,sex,so,gm.stereotype,gm.affectval,gm.equality,z.gm.global,gm.global,lw.s
```

Demographic Statistics

```
    summarise(participant.data,
              mean.age = mean(age,na.rm = TRUE),
              sd.age = sd(age,na.rm = TRUE))
```

```
##   mean.age   sd.age
## 1 25.86127 8.616058
```

```
gender.table <- data.frame(
  Variable = c("Gender - Men", "Gender - Women", "Gender - Non-binary", "Gender - Other", "Gender - Und
Count = c(table(data$gender)["1"], table(data$gender)["2"], table(data$gender)["3"], table(data$gender)

print(gender.table)
```

```
##               Variable Count
## 1         Gender - Men    88
## 2       Gender - Women    98
## 3  Gender - Non-binary    18
## 4       Gender - Other     4
## 5 Gender - Undisclosed     3
```

```
so.table <- data.frame(
  Variable = c("Sexual Orientation - Heterosexual", "Sexual Orientation - Homosexual", "Sexual Orientat
Count = c(table(data$so)["1"], table(data$so)["2"], table(data$so)["3"], table(data$so)["4"], table(dat

print(so.table)
```

```
##                            Variable Count
## 1 Sexual Orientation - Heterosexual    98
## 2   Sexual Orientation - Homosexual    23
## 3     Sexual Orientation - Bisexual    59
## 4        Sexual Orientation - Other    27
## 5  Sexual Orientation - Undisclosed     5
```

Check for correlation

```
cor.test(~z.GRSS + z.sps,participant.data)
```

```
##
##  Pearson's product-moment correlation
##
## data:  z.GRSS and z.sps
## t = 10.562, df = 167, p-value < 2.2e-16
```

```
## alternative hypothesis: true correlation is not equal to 0
## 95 percent confidence interval:
##  0.5327825 0.7154534
## sample estimates:
##       cor
## 0.6328436
```

```
cor.test(~ z.gm.global + z.lw.global,participant.data)
```

```
##
##  Pearson's product-moment correlation
##
## data:  z.gm.global and z.lw.global
## t = 39.414, df = 167, p-value < 2.2e-16
## alternative hypothesis: true correlation is not equal to 0
## 95 percent confidence interval:
##  0.9331204 0.9630427
## sample estimates:
##       cor
## 0.9502277
```

Calculate Cronbach's alpha for GRSS

```
GRSS.data <- data %>%
  select(gorilla.id, GRSS1:GRSS8) %>%
  na.omit() %>%
  mutate(GRSS1R = recode(GRSS1, "1"=5,"2"=4,"3"=3,"4"=2,"5"=1),
         GRSS3R = recode(GRSS3, "1"=5,"2"=4,"3"=3,"4"=2,"5"=1),
         GRSS4R = recode(GRSS4, "1"=5,"2"=4,"3"=3,"4"=2,"5"=1),
         GRSS8R = recode(GRSS8, "1"=5,"2"=4,"3"=3,"4"=2,"5"=1)) %>%
  select(-gorilla.id,-GRSS1,-GRSS3,-GRSS4,-GRSS8)

GRSS.alpha <- alpha(GRSS.data)
print(GRSS.alpha)
```

```
##
## Reliability analysis
## Call: alpha(x = GRSS.data)
##
##   raw_alpha std.alpha G6(smc) average_r S/N   ase mean   sd median_r
##       0.81      0.81    0.83      0.35 4.3 0.018  3.3 0.34     0.31
##
##     95% confidence boundaries
##          lower alpha upper
## Feldt     0.77  0.81  0.85
## Duhachek  0.78  0.81  0.85
##
##  Reliability if an item is dropped:
##        raw_alpha std.alpha G6(smc) average_r S/N alpha se var.r med.r
## GRSS2       0.82      0.82    0.84      0.39 4.4    0.019 0.039  0.36
## GRSS5       0.81      0.81    0.82      0.38 4.3    0.019 0.038  0.36
```

```
## GRSS6       0.79      0.78    0.79      0.34 3.5    0.020 0.038  0.28
## GRSS7       0.83      0.82    0.84      0.40 4.7    0.017 0.031  0.36
## GRSS1R      0.76      0.76    0.78      0.31 3.2    0.023 0.031  0.31
## GRSS3R      0.80      0.79    0.81      0.34 3.7    0.020 0.041  0.31
## GRSS4R      0.75      0.75    0.77      0.30 3.0    0.026 0.030  0.26
## GRSS8R      0.76      0.76    0.78      0.32 3.2    0.024 0.030  0.31
##
##  Item statistics
##          n raw.r std.r r.cor r.drop mean   sd
## GRSS2  179  0.41  0.50  0.37   0.32  3.1 0.27
## GRSS5  179  0.46  0.53  0.43   0.36  3.1 0.33
## GRSS6  179  0.65  0.70  0.66   0.55  3.1 0.39
## GRSS7  179  0.44  0.43  0.30   0.28  3.2 0.49
## GRSS1R 179  0.80  0.78  0.78   0.71  3.3 0.55
## GRSS3R 179  0.73  0.67  0.59   0.56  3.7 0.73
## GRSS4R 179  0.87  0.85  0.86   0.80  3.4 0.60
## GRSS8R 179  0.81  0.78  0.78   0.71  3.4 0.60
##
## Non missing response frequency for each item
##           1    2    3    4    5 miss
## GRSS2  0.00 0.00 0.96 0.03 0.01    0
## GRSS5  0.00 0.01 0.92 0.06 0.01    0
## GRSS6  0.00 0.00 0.91 0.07 0.02    0
## GRSS7  0.01 0.01 0.75 0.23 0.01    0
## GRSS1R 0.00 0.01 0.70 0.26 0.03    0
## GRSS3R 0.00 0.00 0.50 0.35 0.15    0
## GRSS4R 0.00 0.00 0.61 0.34 0.06    0
## GRSS8R 0.00 0.00 0.62 0.32 0.06    0
```

Calculate Cronbach's alpha for SPS

```r
SPS.data <- data %>%
  select(gorilla.id, g.SPS4:l.SPS23) %>%
  na.omit() %>%
  mutate(g.SPS16R = recode(g.SPS16, "1"=6,"2"=5,"3"=4,"4"=3,"5"=2,"6"=1),
         g.SPS19R = recode(g.SPS19, "1"=6,"2"=5,"3"=4,"4"=3,"5"=2,"6"=1),
         g.SPS20R = recode(g.SPS20, "1"=6,"2"=5,"3"=4,"4"=3,"5"=2,"6"=1),
         g.SPS21R = recode(g.SPS21, "1"=6,"2"=5,"3"=4,"4"=3,"5"=2,"6"=1),
         g.SPS23R = recode(g.SPS23, "1"=6,"2"=5,"3"=4,"4"=3,"5"=2,"6"=1),
         l.SPS17R = recode(l.SPS17, "1"=6,"2"=5,"3"=4,"4"=3,"5"=2,"6"=1),
         l.SPS18R = recode(l.SPS18, "1"=6,"2"=5,"3"=4,"4"=3,"5"=2,"6"=1),
         l.SPS19R = recode(l.SPS19, "1"=6,"2"=5,"3"=4,"4"=3,"5"=2,"6"=1),
         l.SPS20R = recode(l.SPS20, "1"=6,"2"=5,"3"=4,"4"=3,"5"=2,"6"=1),
         l.SPS23R = recode(l.SPS23, "1"=6,"2"=5,"3"=4,"4"=3,"5"=2,"6"=1)) %>%
  select(-gorilla.id,-g.SPS16,-g.SPS19,-g.SPS20,-g.SPS21,-g.SPS23,-l.SPS17,-l.SPS18,-l.SPS19,-l.SPS20,-[illegible]

SPS.alpha <- alpha(SPS.data)
print(SPS.alpha)
```

```
##
## Reliability analysis
## Call: alpha(x = SPS.data)
```

```
##
##   raw_alpha std.alpha G6(smc) average_r S/N    ase mean   sd median_r
##       0.97      0.97    0.98      0.54  35 0.0033  1.9 0.88     0.52
##
##     95% confidence boundaries
##          lower alpha upper
## Feldt     0.96  0.97  0.98
## Duhachek  0.96  0.97  0.98
##
##  Reliability if an item is dropped:
##          raw_alpha std.alpha G6(smc) average_r S/N alpha se var.r med.r
## g.SPS4        0.97      0.97    0.98      0.55  35   0.0033 0.026  0.54
## g.SPS6        0.97      0.97    0.98      0.55  35   0.0033 0.027  0.54
## g.SPS8        0.97      0.97    0.98      0.55  35   0.0033 0.027  0.54
## g.SPS11       0.97      0.97    0.98      0.54  34   0.0035 0.027  0.51
## g.SPS13       0.97      0.97    0.98      0.54  35   0.0034 0.027  0.53
## g.SPS2        0.97      0.97    0.98      0.53  33   0.0036 0.025  0.51
## g.SPS3        0.97      0.97    0.98      0.53  33   0.0036 0.025  0.51
## g.SPS5        0.97      0.97    0.98      0.53  33   0.0036 0.026  0.51
## g.SPS7        0.97      0.97    0.98      0.53  33   0.0036 0.025  0.51
## g.SPS9        0.97      0.97    0.98      0.54  33   0.0035 0.027  0.51
## l.SPS1        0.97      0.97    0.98      0.54  34   0.0034 0.027  0.54
## l.SPS3        0.97      0.97    0.98      0.55  35   0.0033 0.026  0.54
## l.SPS5        0.97      0.97    0.98      0.55  36   0.0033 0.025  0.54
## l.SPS7        0.97      0.97    0.98      0.54  35   0.0034 0.027  0.54
## l.SPS11       0.97      0.97    0.98      0.54  34   0.0034 0.028  0.51
## l.SPS4        0.97      0.97    0.98      0.53  33   0.0035 0.026  0.51
## l.SPS9        0.97      0.97    0.98      0.53  33   0.0036 0.025  0.51
## l.SPS10       0.97      0.97    0.98      0.54  34   0.0035 0.026  0.51
## l.SPS15       0.97      0.97    0.98      0.53  33   0.0035 0.026  0.51
## l.SPS8        0.97      0.97    0.98      0.53  33   0.0036 0.026  0.51
## g.SPS16R      0.97      0.97    0.98      0.54  34   0.0035 0.026  0.51
## g.SPS19R      0.97      0.97    0.98      0.54  34   0.0035 0.027  0.51
## g.SPS20R      0.97      0.97    0.98      0.54  33   0.0035 0.027  0.51
## g.SPS21R      0.97      0.97    0.98      0.54  33   0.0035 0.026  0.51
## g.SPS23R      0.97      0.97    0.98      0.53  33   0.0035 0.027  0.51
## l.SPS17R      0.97      0.97    0.98      0.55  36   0.0031 0.024  0.54
## l.SPS18R      0.97      0.97    0.98      0.54  34   0.0034 0.027  0.51
## l.SPS19R      0.97      0.97    0.98      0.53  33   0.0036 0.026  0.51
## l.SPS20R      0.97      0.97    0.98      0.54  34   0.0034 0.026  0.52
## l.SPS23R      0.97      0.97    0.98      0.54  34   0.0035 0.027  0.52
##
##  Item statistics
##            n raw.r std.r r.cor r.drop mean   sd
## g.SPS4   169  0.56  0.56  0.54   0.53  2.5 1.38
## g.SPS6   169  0.60  0.60  0.59   0.57  2.7 1.31
## g.SPS8   169  0.61  0.60  0.59   0.57  2.5 1.41
## g.SPS11  169  0.76  0.77  0.76   0.74  1.5 0.85
## g.SPS13  169  0.65  0.65  0.64   0.62  1.9 1.09
## g.SPS2   169  0.89  0.89  0.90   0.88  1.6 1.26
## g.SPS3   169  0.87  0.88  0.88   0.86  1.5 1.14
## g.SPS5   169  0.84  0.84  0.83   0.82  1.8 1.42
## g.SPS7   169  0.90  0.90  0.90   0.89  1.5 1.11
## g.SPS9   169  0.81  0.80  0.80   0.79  1.6 1.24
```

```
## l.SPS1   169  0.66  0.66  0.65   0.63  2.3 1.12
## l.SPS3   169  0.55  0.55  0.52   0.52  2.8 1.42
## l.SPS5   169  0.49  0.49  0.47   0.46  2.3 1.01
## l.SPS7   169  0.64  0.64  0.62   0.61  2.9 1.36
## l.SPS11  169  0.73  0.74  0.73   0.71  1.8 0.98
## l.SPS4   169  0.85  0.86  0.86   0.84  1.6 1.05
## l.SPS9   169  0.89  0.89  0.90   0.88  1.5 1.16
## l.SPS10  169  0.78  0.78  0.78   0.76  1.7 1.30
## l.SPS15  169  0.84  0.85  0.85   0.83  1.4 0.95
## l.SPS8   169  0.90  0.90  0.90   0.89  1.7 1.17
## g.SPS16R 169  0.78  0.78  0.77   0.76  1.6 1.22
## g.SPS19R 169  0.78  0.78  0.78   0.76  1.7 1.15
## g.SPS20R 169  0.80  0.81  0.80   0.79  1.6 1.10
## g.SPS21R 169  0.80  0.80  0.80   0.78  1.8 1.18
## g.SPS23R 169  0.83  0.83  0.83   0.81  1.7 1.11
## l.SPS17R 169  0.48  0.46  0.44   0.43  2.0 1.73
## l.SPS18R 169  0.75  0.76  0.75   0.73  1.3 0.73
## l.SPS19R 169  0.86  0.85  0.85   0.84  1.8 1.31
## l.SPS20R 169  0.68  0.67  0.67   0.65  1.8 1.17
## l.SPS23R 169  0.74  0.74  0.74   0.72  1.8 1.24
##
## Non missing response frequency for each item
##             1    2    3    4    5    6 miss
## g.SPS4   0.28 0.34 0.12 0.18 0.05 0.04    0
## g.SPS6   0.20 0.31 0.17 0.24 0.06 0.02    0
## g.SPS8   0.29 0.33 0.15 0.12 0.07 0.04    0
## g.SPS11  0.67 0.26 0.04 0.01 0.01 0.01    0
## g.SPS13  0.43 0.40 0.09 0.04 0.02 0.02    0
## g.SPS2   0.76 0.09 0.04 0.04 0.04 0.03    0
## g.SPS3   0.78 0.11 0.03 0.03 0.02 0.03    0
## g.SPS5   0.70 0.09 0.05 0.07 0.06 0.03    0
## g.SPS7   0.77 0.12 0.03 0.04 0.03 0.02    0
## g.SPS9   0.70 0.14 0.05 0.07 0.02 0.03    0
## l.SPS1   0.24 0.41 0.15 0.17 0.02 0.01    0
## l.SPS3   0.20 0.33 0.16 0.19 0.08 0.05    0
## l.SPS5   0.20 0.48 0.22 0.07 0.02 0.01    0
## l.SPS7   0.15 0.33 0.15 0.25 0.08 0.04    0
## l.SPS11  0.47 0.36 0.09 0.07 0.01 0.01    0
## l.SPS4   0.65 0.21 0.06 0.05 0.02 0.01    0
## l.SPS9   0.79 0.09 0.04 0.04 0.02 0.03    0
## l.SPS10  0.66 0.17 0.03 0.08 0.04 0.02    0
## l.SPS15  0.80 0.08 0.08 0.01 0.01 0.02    0
## l.SPS8   0.65 0.18 0.07 0.05 0.03 0.02    0
## g.SPS16R 0.75 0.13 0.03 0.04 0.02 0.04    0
## g.SPS19R 0.63 0.18 0.09 0.05 0.02 0.02    0
## g.SPS20R 0.66 0.20 0.06 0.04 0.03 0.01    0
## g.SPS21R 0.57 0.24 0.09 0.04 0.04 0.02    0
## g.SPS23R 0.61 0.22 0.08 0.05 0.02 0.02    0
## l.SPS17R 0.67 0.11 0.04 0.02 0.05 0.11    0
## l.SPS18R 0.80 0.15 0.03 0.01 0.01 0.01    0
## l.SPS19R 0.62 0.17 0.06 0.07 0.07 0.01    0
## l.SPS20R 0.57 0.22 0.11 0.06 0.02 0.02    0
## l.SPS23R 0.58 0.20 0.11 0.05 0.03 0.02    0
```

Calculate Cronbach's alpha for SPS seperately for gay men and lesbian women

```
gmSPS.data <- SPS.data %>%
  select(starts_with("g"))

gmSPS.alpha <- alpha(gmSPS.data)
print(gmSPS.alpha)
```

```
##
## Reliability analysis
## Call: alpha(x = gmSPS.data)
##
##   raw_alpha std.alpha G6(smc) average_r S/N    ase mean   sd median_r
##       0.95      0.95    0.97      0.57  20 0.0057  1.8 0.92     0.57
##
##     95% confidence boundaries
##          lower alpha upper
## Feldt     0.94  0.95  0.96
## Duhachek  0.94  0.95  0.96
##
##  Reliability if an item is dropped:
##          raw_alpha std.alpha G6(smc) average_r S/N alpha se var.r med.r
## g.SPS4        0.95      0.95    0.96      0.60  21   0.0055 0.021  0.60
## g.SPS6        0.95      0.95    0.97      0.59  20   0.0056 0.022  0.60
## g.SPS8        0.95      0.95    0.96      0.59  20   0.0055 0.023  0.60
## g.SPS11       0.95      0.95    0.96      0.57  19   0.0061 0.026  0.57
## g.SPS13       0.95      0.95    0.96      0.58  20   0.0059 0.025  0.60
## g.SPS2        0.94      0.95    0.96      0.55  17   0.0066 0.021  0.56
## g.SPS3        0.94      0.95    0.96      0.56  18   0.0065 0.021  0.56
## g.SPS5        0.94      0.95    0.96      0.56  18   0.0064 0.024  0.56
## g.SPS7        0.94      0.95    0.96      0.55  17   0.0065 0.021  0.56
## g.SPS9        0.94      0.95    0.96      0.57  18   0.0063 0.024  0.56
## g.SPS16R      0.94      0.95    0.96      0.57  18   0.0062 0.023  0.56
## g.SPS19R      0.95      0.95    0.96      0.57  19   0.0062 0.023  0.56
## g.SPS20R      0.94      0.95    0.96      0.57  18   0.0062 0.024  0.56
## g.SPS21R      0.94      0.95    0.96      0.57  18   0.0062 0.023  0.56
## g.SPS23R      0.94      0.95    0.96      0.56  18   0.0063 0.023  0.56
##
##  Item statistics
##            n raw.r std.r r.cor r.drop mean   sd
## g.SPS4   169  0.59  0.58  0.54   0.52  2.5 1.38
## g.SPS6   169  0.61  0.60  0.56   0.54  2.7 1.31
## g.SPS8   169  0.63  0.62  0.58   0.56  2.5 1.41
## g.SPS11  169  0.77  0.78  0.76   0.74  1.5 0.85
## g.SPS13  169  0.67  0.67  0.64   0.62  1.9 1.09
## g.SPS2   169  0.90  0.90  0.91   0.88  1.6 1.26
## g.SPS3   169  0.88  0.89  0.89   0.86  1.5 1.14
## g.SPS5   169  0.84  0.83  0.83   0.80  1.8 1.42
## g.SPS7   169  0.90  0.90  0.91   0.88  1.5 1.11
## g.SPS9   169  0.82  0.81  0.80   0.78  1.6 1.24
## g.SPS16R 169  0.80  0.80  0.79   0.76  1.6 1.22
## g.SPS19R 169  0.77  0.78  0.77   0.74  1.7 1.15
## g.SPS20R 169  0.81  0.81  0.80   0.78  1.6 1.10
```

```
## g.SPS21R 169  0.80  0.81  0.80   0.77  1.8 1.18
## g.SPS23R 169  0.82  0.83  0.82   0.79  1.7 1.11
##
## Non missing response frequency for each item
##             1    2    3    4    5    6 miss
## g.SPS4   0.28 0.34 0.12 0.18 0.05 0.04    0
## g.SPS6   0.20 0.31 0.17 0.24 0.06 0.02    0
## g.SPS8   0.29 0.33 0.15 0.12 0.07 0.04    0
## g.SPS11  0.67 0.26 0.04 0.01 0.01 0.01    0
## g.SPS13  0.43 0.40 0.09 0.04 0.02 0.02    0
## g.SPS2   0.76 0.09 0.04 0.04 0.04 0.03    0
## g.SPS3   0.78 0.11 0.03 0.03 0.02 0.03    0
## g.SPS5   0.70 0.09 0.05 0.07 0.06 0.03    0
## g.SPS7   0.77 0.12 0.03 0.04 0.03 0.02    0
## g.SPS9   0.70 0.14 0.05 0.07 0.02 0.03    0
## g.SPS16R 0.75 0.13 0.03 0.04 0.02 0.04    0
## g.SPS19R 0.63 0.18 0.09 0.05 0.02 0.02    0
## g.SPS20R 0.66 0.20 0.06 0.04 0.03 0.01    0
## g.SPS21R 0.57 0.24 0.09 0.04 0.04 0.02    0
## g.SPS23R 0.61 0.22 0.08 0.05 0.02 0.02    0
```

```r
lwSPS.data <- SPS.data %>%
  select(starts_with("l"))

lwSPS.alpha <- alpha(lwSPS.data)
print(lwSPS.alpha)
```

```
##
## Reliability analysis
## Call: alpha(x = lwSPS.data)
##
##   raw_alpha std.alpha G6(smc) average_r S/N    ase mean   sd median_r
##       0.93      0.94    0.95       0.5  15 0.0079  1.9 0.85     0.48
##
##     95% confidence boundaries
##          lower alpha upper
## Feldt     0.91  0.93  0.94
## Duhachek  0.91  0.93  0.94
##
##  Reliability if an item is dropped:
##          raw_alpha std.alpha G6(smc) average_r S/N alpha se var.r med.r
## l.SPS1        0.93      0.94    0.95      0.51  14   0.0082 0.028  0.49
## l.SPS3        0.93      0.94    0.95      0.52  15   0.0078 0.028  0.50
## l.SPS5        0.93      0.94    0.95      0.53  16   0.0079 0.025  0.50
## l.SPS7        0.93      0.94    0.95      0.51  15   0.0082 0.029  0.49
## l.SPS11       0.92      0.93    0.95      0.50  14   0.0085 0.029  0.46
## l.SPS4        0.92      0.93    0.95      0.49  13   0.0089 0.026  0.45
## l.SPS9        0.92      0.93    0.95      0.48  13   0.0090 0.024  0.46
## l.SPS10       0.92      0.93    0.95      0.50  14   0.0087 0.026  0.47
## l.SPS15       0.92      0.93    0.95      0.49  13   0.0087 0.025  0.47
## l.SPS8        0.92      0.93    0.95      0.48  13   0.0091 0.025  0.45
## l.SPS17R      0.94      0.94    0.96      0.53  16   0.0071 0.024  0.50
## l.SPS18R      0.93      0.93    0.95      0.50  14   0.0084 0.028  0.47
## l.SPS19R      0.92      0.93    0.95      0.49  13   0.0091 0.027  0.44
```

```
## l.SPS20R      0.93      0.94    0.95      0.51  14    0.0083 0.027  0.49
## l.SPS23R      0.92      0.93    0.95      0.50  14    0.0085 0.028  0.47
##
##  Item statistics
##             n raw.r std.r r.cor r.drop mean   sd
## l.SPS1   169  0.67  0.67  0.65   0.61  2.3 1.12
## l.SPS3   169  0.59  0.57  0.52   0.51  2.8 1.42
## l.SPS5   169  0.52  0.53  0.48   0.46  2.3 1.01
## l.SPS7   169  0.67  0.67  0.64   0.61  2.9 1.36
## l.SPS11  169  0.74  0.76  0.74   0.71  1.8 0.98
## l.SPS4   169  0.85  0.85  0.85   0.82  1.6 1.05
## l.SPS9   169  0.87  0.88  0.88   0.84  1.5 1.16
## l.SPS10  169  0.78  0.78  0.77   0.73  1.7 1.30
## l.SPS15  169  0.83  0.84  0.85   0.81  1.4 0.95
## l.SPS8   169  0.89  0.90  0.90   0.87  1.7 1.17
## l.SPS17R 169  0.54  0.51  0.46   0.44  2.0 1.73
## l.SPS18R 169  0.74  0.76  0.74   0.71  1.3 0.73
## l.SPS19R 169  0.86  0.85  0.84   0.83  1.8 1.31
## l.SPS20R 169  0.68  0.68  0.66   0.63  1.8 1.17
## l.SPS23R 169  0.73  0.73  0.71   0.68  1.8 1.24
##
## Non missing response frequency for each item
##             1    2    3    4    5    6 miss
## l.SPS1   0.24 0.41 0.15 0.17 0.02 0.01    0
## l.SPS3   0.20 0.33 0.16 0.19 0.08 0.05    0
## l.SPS5   0.20 0.48 0.22 0.07 0.02 0.01    0
## l.SPS7   0.15 0.33 0.15 0.25 0.08 0.04    0
## l.SPS11  0.47 0.36 0.09 0.07 0.01 0.01    0
## l.SPS4   0.65 0.21 0.06 0.05 0.02 0.01    0
## l.SPS9   0.79 0.09 0.04 0.04 0.02 0.03    0
## l.SPS10  0.66 0.17 0.03 0.08 0.04 0.02    0
## l.SPS15  0.80 0.08 0.08 0.01 0.01 0.02    0
## l.SPS8   0.65 0.18 0.07 0.05 0.03 0.02    0
## l.SPS17R 0.67 0.11 0.04 0.02 0.05 0.11    0
## l.SPS18R 0.80 0.15 0.03 0.01 0.01 0.01    0
## l.SPS19R 0.62 0.17 0.06 0.07 0.07 0.01    0
## l.SPS20R 0.57 0.22 0.11 0.06 0.02 0.02    0
## l.SPS23R 0.58 0.20 0.11 0.05 0.03 0.02    0
```

Create datasets for response data and image data, and join to create trial data

```r
response.data <- data %>%
  select(gorilla.id,response.1:response.9) %>%
  pivot_longer(cols = starts_with("response."), names_to = "trial.no", values_to = "response") %>%
  separate(trial.no,into = c("leftovers","trial.no"),sep = 9) %>%
  select(-leftovers)

image.data <- data %>%
  select(gorilla.id,face.1:face.9) %>%
  pivot_longer(cols = starts_with("face."), names_to = "trial.image", values_to = "image") %>%
  filter(!is.na(image)) %>%
  separate(trial.image,into = c("leftovers","trial.no"),sep = 5) %>%
```

```
  select(-leftovers)

trial.data <- response.data %>%
  full_join(image.data, by = c("gorilla.id","trial.no"))
```

Prepare face dataset

Perceived gender typicality scores are calculated using masculinity/femininity ratings from the Chicago Face Dataset (Ma et al., 2015). Shape sexual dimorphism scores (calculated using geometric morphometric methods) are used to calculate objective gender typicality scores.

```
prep.face.data <- face.data %>%
  mutate(r.feminine = (Feminine * -1)+8,
         perceived.score = (r.feminine+Masculine)/2) %>%
  group_by(Gender) %>%
  mutate(gc.sexual.dimorphism = sexual.dimorphism - mean(sexual.dimorphism),
         gc.perceived.score = perceived.score - mean(perceived.score)) %>%
  mutate(perceived.typicality = ifelse(Gender == "F",(perceived.score * -1)+8,perceived.score),
         objective.typicality = ifelse(Gender == "F",gc.sexual.dimorphism * -1,gc.sexual.dimorphism)) %
  mutate(z.perceived.score = c(scale(perceived.typicality)),
         z.objective.score = c(scale(objective.typicality))) %>%
  ungroup()

final.data <- trial.data %>%
  full_join(prep.face.data, by = "image")
```

Check correlations

```
cor(select(prep.face.data,Masculine,Feminine,perceived.score,gc.perceived.score,sexual.dimorphism,gc.se
```

```
##                        Masculine    Feminine perceived.score gc.perceived.score
## Masculine              1.0000000 -0.93672385       0.9826961          0.3605134
## Feminine              -0.9367238  1.00000000      -0.9853565         -0.3007593
## perceived.score        0.9826961 -0.98535650       1.0000000          0.3347286
## gc.perceived.score     0.3605134 -0.30075930       0.3347286          1.0000000
## sexual.dimorphism      0.7270881 -0.64021065       0.6928819          0.2527601
## gc.sexual.dimorphism   0.1788261 -0.04474885       0.1107772          0.3309463
##                       sexual.dimorphism gc.sexual.dimorphism
## Masculine                     0.7270881           0.17882614
## Feminine                     -0.6402106          -0.04474885
## perceived.score               0.6928819           0.11077719
## gc.perceived.score            0.2527601           0.33094628
## sexual.dimorphism             1.0000000           0.76374972
## gc.sexual.dimorphism          0.7637497           1.00000000
```

```
cor.test(~Masculine + Feminine,prep.face.data)
```

```
##
##  Pearson's product-moment correlation
```

```
##
## data:  Masculine and Feminine
## t = -23.632, df = 78, p-value < 2.2e-16
## alternative hypothesis: true correlation is not equal to 0
## 95 percent confidence interval:
##  -0.959054 -0.902820
## sample estimates:
##        cor
## -0.9367238
```

```
cor.test(~ perceived.score + sexual.dimorphism,prep.face.data)
```

```
##
##  Pearson's product-moment correlation
##
## data:  perceived.score and sexual.dimorphism
## t = 8.4867, df = 78, p-value = 1.07e-12
## alternative hypothesis: true correlation is not equal to 0
## 95 percent confidence interval:
##  0.5581341 0.7920229
## sample estimates:
##       cor
## 0.6928819
```

```
cor.test(~ z.perceived.score + z.objective.score,prep.face.data)
```

```
##
##  Pearson's product-moment correlation
##
## data:  z.perceived.score and z.objective.score
## t = 3.4454, df = 78, p-value = 0.0009209
## alternative hypothesis: true correlation is not equal to 0
## 95 percent confidence interval:
##  0.1561960 0.5400332
## sample estimates:
##       cor
## 0.3634401
```

```
ggplot(prep.face.data,aes(x = z.objective.score,y = z.perceived.score,group = Gender,colour = Gender)) +
  geom_point() +
  geom_smooth(method = "lm")
```

```
## `geom_smooth()` using formula = 'y ~ x'
```

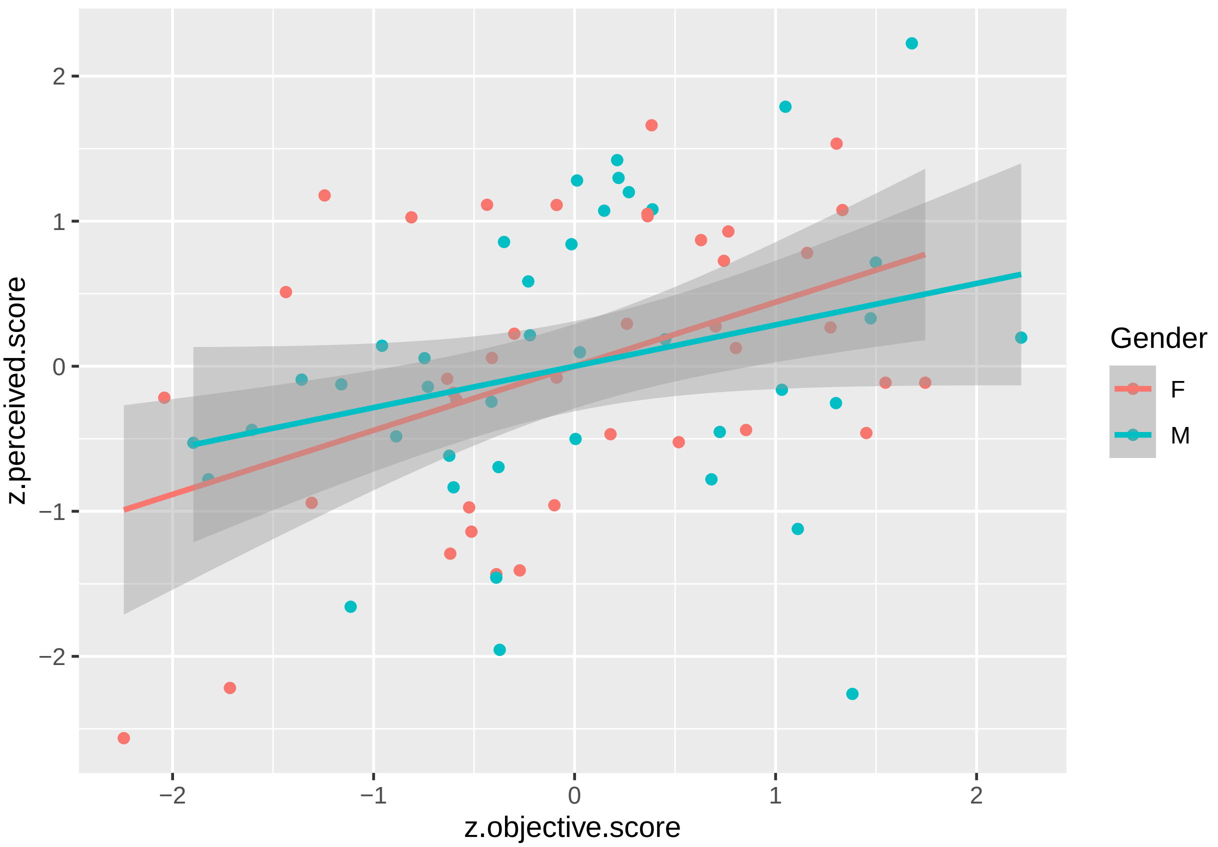

Analysis data ### Add participant data to trial data

```
analysis.data <- final.data %>%
  full_join(participant.data, by = "gorilla.id") %>%
  mutate(gender.match = ifelse(Gender == "M" & gender == "1", "same",
                        ifelse(Gender == "F" & gender == "1", "opposite",
                               ifelse(Gender == "F" & gender == "2", "same",
                                      ifelse(Gender == "M" & gender == "2", "opposite",NA_character_
```

#Primary analyses ## GRSS model

```
GRSS.model <- glmer(response ~ z.GRSS*z.objective.score + z.GRSS*z.perceived.score +
                      (1 + z.GRSS || image) +
                      (1 + z.objective.score + z.perceived.score || gorilla.id), data = analysis.data, f

summary(GRSS.model)
```

```
## Generalized linear mixed model fit by maximum likelihood (Laplace
##   Approximation) [glmerMod]
##  Family: binomial  ( logit )
## Formula: response ~ z.GRSS * z.objective.score + z.GRSS * z.perceived.score +
##     (1 + z.GRSS || image) + (1 + z.objective.score + z.perceived.score ||
##     gorilla.id)
##    Data: analysis.data
##
##      AIC      BIC   logLik deviance df.resid
```

```
##  14451.7  14534.6  -7214.8  14429.7    13867
##
## Scaled residuals:
##     Min      1Q  Median      3Q     Max
## -3.4444 -0.6247 -0.3581  0.7475  5.6632
##
## Random effects:
##  Groups       Name              Variance Std.Dev.
##  gorilla.id   z.perceived.score 0.04154  0.2038
##  gorilla.id.1 z.objective.score 0.01316  0.1147
##  gorilla.id.2 (Intercept)       1.98429  1.4086
##  image        z.GRSS            0.02790  0.1670
##  image.1      (Intercept)       0.22226  0.4714
## Number of obs: 13878, groups:  gorilla.id, 175; image, 80
##
## Fixed effects:
##                           Estimate Std. Error z value Pr(>|z|)
## (Intercept)               -1.18339    0.12220  -9.684  < 2e-16 ***
## z.GRSS                    -0.02611    0.11192  -0.233  0.81557
## z.objective.score         -0.13166    0.06244  -2.109  0.03497 *
## z.perceived.score         -0.18989    0.06396  -2.969  0.00299 **
## z.GRSS:z.objective.score   0.02026    0.03251   0.623  0.53311
## z.GRSS:z.perceived.score -0.15712    0.03563  -4.410 1.03e-05 ***
## ---
## Signif. codes:  0 '***' 0.001 '**' 0.01 '*' 0.05 '.' 0.1 ' ' 1
##
## Correlation of Fixed Effects:
##             (Intr) z.GRSS z.bjc. z.prc. z.GRSS:z.b.
## z.GRSS      -0.015
## z.bjctv.scr  0.004  0.000
## z.prcvd.scr  0.006  0.004 -0.344
## z.GRSS:z.b. -0.001  0.006 -0.007  0.005
## z.GRSS:z.p.  0.009  0.020  0.005 -0.002 -0.293
```

```
GRSS.model2 <- glmer(response ~ z.GRSS*z.objective.score + z.GRSS*z.perceived.score +
                     (1 + z.GRSS | image) +
                     (1 + z.objective.score + z.perceived.score | gorilla.id), data = analysis.data, fa
```

```
## Warning in checkConv(attr(opt, "derivs"), opt$par, ctrl = control$checkConv, :
## Model failed to converge with max|grad| = 0.0184883 (tol = 0.002, component 1)
```

```
summary(GRSS.model2)
```

```
## Generalized linear mixed model fit by maximum likelihood (Laplace
##   Approximation) [glmerMod]
##  Family: binomial  ( logit )
## Formula: response ~ z.GRSS * z.objective.score + z.GRSS * z.perceived.score +
##     (1 + z.GRSS | image) + (1 + z.objective.score + z.perceived.score |
##     gorilla.id)
##    Data: analysis.data
##
##      AIC      BIC   logLik deviance df.resid
##    14454    14567    -7212    14424    13863
```

```
##
## Scaled residuals:
##     Min      1Q  Median      3Q     Max
## -3.6589 -0.6248 -0.3564  0.7498  5.3599
##
## Random effects:
##  Groups     Name              Variance Std.Dev. Corr
##  gorilla.id (Intercept)       2.000404 1.41436
##             z.objective.score 0.008131 0.09017  -0.15
##             z.perceived.score 0.037047 0.19248   0.31  0.68
##  image      (Intercept)       0.222537 0.47174
##             z.GRSS            0.027335 0.16533  -0.27
## Number of obs: 13878, groups:  gorilla.id, 175; image, 80
##
## Fixed effects:
##                          Estimate Std. Error z value Pr(>|z|)
## (Intercept)              -1.18719    0.12270  -9.676  < 2e-16 ***
## z.GRSS                   -0.02415    0.11243  -0.215  0.82990
## z.objective.score        -0.12695    0.06338  -2.003  0.04518 *
## z.perceived.score        -0.20614    0.06483  -3.180  0.00147 **
## z.GRSS:z.objective.score  0.02212    0.03177   0.696  0.48626
## z.GRSS:z.perceived.score -0.15744    0.03503  -4.494 6.99e-06 ***
## ---
## Signif. codes:  0 '***' 0.001 '**' 0.01 '*' 0.05 '.' 0.1 ' ' 1
##
## Correlation of Fixed Effects:
##             (Intr) z.GRSS z.bjc. z.prc. z.GRSS:z.b.
## z.GRSS      -0.033
## z.bjctv.scr -0.008  0.000
## z.prcvd.scr  0.075  0.006 -0.328
## z.GRSS:z.b.  0.000 -0.026 -0.153  0.055
## z.GRSS:z.p.  0.008  0.152  0.051 -0.131 -0.228
## optimizer (Nelder_Mead) convergence code: 0 (OK)
## Model failed to converge with max|grad| = 0.0184883 (tol = 0.002, component 1)
```

Calculate R-squared

```r
library(MuMIn)

r.squaredGLMM(GRSS.model2)
```

```
## Warning: the null model is only correct if all the variables it uses are identical
## to those used in fitting the original model.
```

```
##                    R2m       R2c
## theoretical 0.01755484 0.4220003
## delta       0.01405531 0.3378752
```

Plot significant interaction of perceived gender typicality score and participant GRSS score

```
grssplot.data <- analysis.data %>%
  mutate(cat.GRSS = ifelse(z.GRSS <= 0,"Low","High")) %>%
  group_by(image,Gender,z.perceived.score,cat.GRSS) %>%
  summarise(prop.response = mean(response)) %>%
  filter(!is.na(cat.GRSS))
```

```
## `summarise()` has grouped output by 'image', 'Gender', 'z.perceived.score'. You
## can override using the `.groups` argument.
```

```
ggplot(grssplot.data,aes(x = z.perceived.score,y = prop.response,group = cat.GRSS,colour = cat.GRSS)) +
  geom_point() +
  geom_smooth(method = "lm") +
  theme_classic() +
  xlab("Perceived facial gender typicality") +
  ylab("Proportion of faces judged as non-heterosexual") +
  labs(colour = "Beliefs in Gender\nRole Stereotypes")
```

```
## `geom_smooth()` using formula = 'y ~ x'

## Warning: Removed 2 rows containing non-finite outside the scale range
## (`stat_smooth()`).

## Warning: Removed 2 rows containing missing values or values outside the scale range
## (`geom_point()`).
```

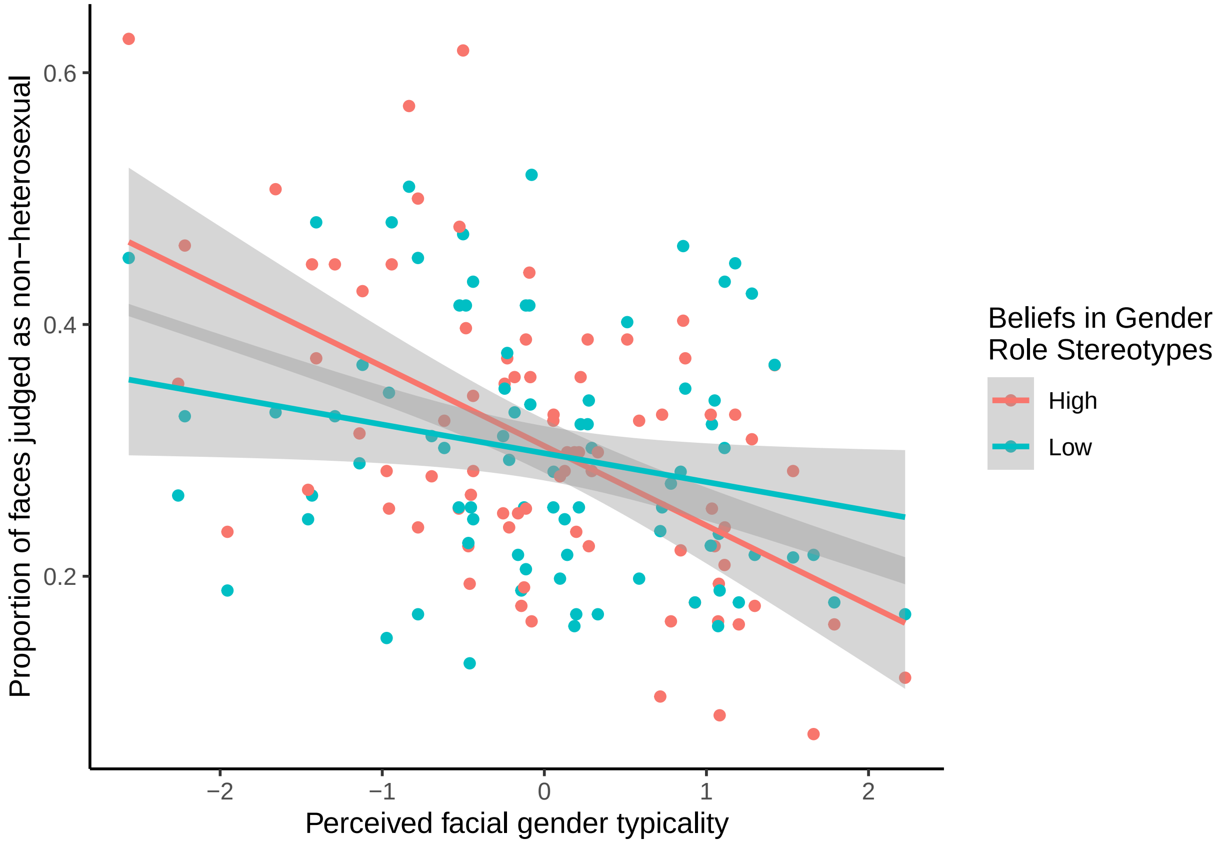

SPS model

```
SPS.model <- glmer(response ~ z.sps*z.objective.score + z.sps*z.perceived.score +
                      (1 + z.sps || image) +
                      (1 + z.objective.score + z.perceived.score || gorilla.id), data = analysis.data, 

summary(SPS.model)
```

```
## Generalized linear mixed model fit by maximum likelihood (Laplace
##   Approximation) [glmerMod]
##  Family: binomial  ( logit )
## Formula: response ~ z.sps * z.objective.score + z.sps * z.perceived.score +
##     (1 + z.sps || image) + (1 + z.objective.score + z.perceived.score ||
##     gorilla.id)
##    Data: analysis.data
##
##      AIC      BIC   logLik deviance df.resid
##  13639.3  13721.7  -6808.7  13617.3    13215
##
## Scaled residuals:
##     Min      1Q  Median      3Q     Max
## -3.3475 -0.6188 -0.3493  0.7222  5.6768
##
```

```
## Random effects:
##  Groups       Name              Variance Std.Dev.
##  gorilla.id   z.perceived.score 0.06132  0.2476
##  gorilla.id.1 z.objective.score 0.01423  0.1193
##  gorilla.id.2 (Intercept)       2.14649  1.4651
##  image        z.sps             0.04332  0.2081
##  image.1      (Intercept)       0.24823  0.4982
## Number of obs: 13226, groups:  gorilla.id, 166; image, 80
##
## Fixed effects:
##                         Estimate Std. Error z value Pr(>|z|)
## (Intercept)             -1.22734    0.13012  -9.432   <2e-16 ***
## z.sps                   -0.16068    0.12268  -1.310   0.1903
## z.objective.score       -0.13245    0.06583  -2.012   0.0442 *
## z.perceived.score       -0.20000    0.06828  -2.929   0.0034 **
## z.sps:z.objective.score  0.02977    0.03839   0.775   0.4381
## z.sps:z.perceived.score -0.08687    0.04329  -2.007   0.0448 *
## ---
## Signif. codes:  0 '***' 0.001 '**' 0.01 '*' 0.05 '.' 0.1 ' ' 1
##
## Correlation of Fixed Effects:
##             (Intr) z.sps  z.bjc. z.prc. z.sps:z.b.
## z.sps        0.010
## z.bjctv.scr  0.004  0.000
## z.prcvd.scr  0.007  0.004 -0.339
## z.sps:z.bj. -0.001  0.005  0.008 -0.003
## z.sps:z.pr.  0.007  0.025 -0.003  0.015 -0.295
```

Calculate R-squared

Run another un-simplified model

```
SPS.model2 <- glmer(response ~ z.sps*z.objective.score + z.sps*z.perceived.score +
                      (1 + z.sps | image) +
                      (1 + z.objective.score + z.perceived.score | gorilla.id), data = analysis.data, fa
```

```
## Warning in checkConv(attr(opt, "derivs"), opt$par, ctrl = control$checkConv, :
## Model failed to converge with max|grad| = 0.00826819 (tol = 0.002, component 1)
```

```
summary(SPS.model2)
```

```
## Generalized linear mixed model fit by maximum likelihood (Laplace
##   Approximation) [glmerMod]
##  Family: binomial  ( logit )
## Formula: response ~ z.sps * z.objective.score + z.sps * z.perceived.score +
##     (1 + z.sps | image) + (1 + z.objective.score + z.perceived.score |
##     gorilla.id)
##    Data: analysis.data
##
##      AIC      BIC   logLik deviance df.resid
##  13636.5  13748.8  -6803.2  13606.5    13211
##
```

```
## Scaled residuals:
##     Min      1Q  Median      3Q     Max
## -3.0766 -0.6198 -0.3461  0.7325  5.6239
##
## Random effects:
##  Groups     Name               Variance Std.Dev. Corr
##  gorilla.id (Intercept)        2.153835 1.46760
##             z.objective.score  0.009709 0.09853  -0.22
##             z.perceived.score  0.056935 0.23861   0.29  0.52
##  image      (Intercept)        0.246382 0.49637
##             z.sps              0.041779 0.20440  -0.48
## Number of obs: 13226, groups:  gorilla.id, 166; image, 80
##
## Fixed effects:
##                           Estimate Std. Error z value Pr(>|z|)
## (Intercept)               -1.23194    0.13037  -9.449  < 2e-16 ***
## z.sps                     -0.15401    0.12319  -1.250  0.21126
## z.objective.score         -0.12480    0.06675  -1.870  0.06153 .
## z.perceived.score         -0.21958    0.06908  -3.179  0.00148 **
## z.sps:z.objective.score    0.03714    0.03839   0.967  0.33331
## z.sps:z.perceived.score   -0.09860    0.04340  -2.272  0.02307 *
## ---
## Signif. codes:  0 '***' 0.001 '**' 0.01 '*' 0.05 '.' 0.1 ' ' 1
##
## Correlation of Fixed Effects:
##              (Intr) z.sps  z.bjc. z.prc. z.sps:z.b.
## z.sps        -0.026
## z.bjctv.scr -0.017 -0.001
## z.prcvd.scr  0.084  0.012 -0.324
## z.sps:z.bj.  0.001 -0.040 -0.231  0.080
## z.sps:z.pr.  0.016  0.164  0.074 -0.190 -0.242
## optimizer (Nelder_Mead) convergence code: 0 (OK)
## Model failed to converge with max|grad| = 0.00826819 (tol = 0.002, component 1)
```

Calculate R-squared values based on the un-simplified model above.

```
r.squaredGLMM(SPS.model2)
```

```
## Warning: the null model is only correct if all the variables it uses are identical
## to those used in fitting the original model.
```

```
##                    R2m       R2c
## theoretical 0.01930055 0.4441590
## delta       0.01549340 0.3565459
```

Plot significant interaction of perceived gender typicality score and participant SPS score

```
spsplot.data <- analysis.data %>%
  mutate(cat.sps = ifelse(z.sps <= 0,"Low","High")) %>%
  group_by(image,Gender,z.perceived.score,cat.sps) %>%
```

```r
  summarise(prop.response = mean(response)) %>%
  filter(!is.na(cat.sps))
```

```
## `summarise()` has grouped output by 'image', 'Gender', 'z.perceived.score'. You
## can override using the `.groups` argument.
```

```r
ggplot(spsplot.data,aes(x = z.perceived.score,y = prop.response,group = cat.sps,colour = cat.sps)) +
  geom_point() +
  geom_smooth(method = "lm") +
  theme_classic() +
  xlab("Perceived facial gender typicality") +
  ylab("Proportion of faces judged as non-heterosexual") +
  labs(colour = "Sexual prejudice")
```

```
## `geom_smooth()` using formula = 'y ~ x'
```

```
## Warning: Removed 1 row containing non-finite outside the scale range
## (`stat_smooth()`).
```

```
## Warning: Removed 1 row containing missing values or values outside the scale range
## (`geom_point()`).
```

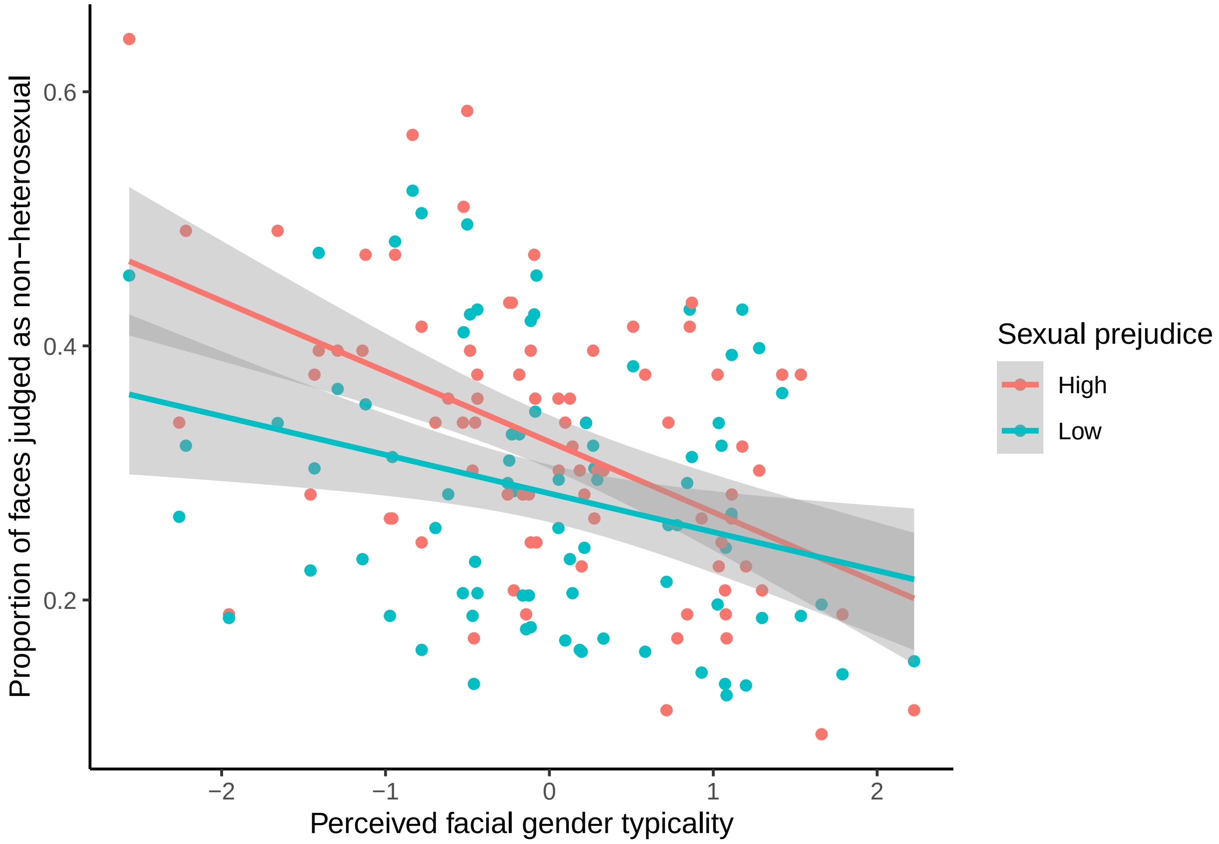

Exploratory analysis

Model with both GRSS and SPS score as predictors

```
all.pred.model <- glmer(response ~ z.GRSS*z.objective.score + z.GRSS*z.perceived.score + z.sps*z.object
                        (1 + z.GRSS + z.sps || image) +
                        (1 + z.objective.score + z.perceived.score || gorilla.id), data = analysis.data,
```

```
## Warning in checkConv(attr(opt, "derivs"), opt$par, ctrl = control$checkConv, :
## Model failed to converge with max|grad| = 0.00898361 (tol = 0.002, component 1)
```

```
summary(all.pred.model)
```

```
## Generalized linear mixed model fit by maximum likelihood (Laplace
##   Approximation) [glmerMod]
##  Family: binomial  ( logit )
## Formula: response ~ z.GRSS * z.objective.score + z.GRSS * z.perceived.score +
##     z.sps * z.objective.score + z.sps * z.perceived.score + (1 +
##     z.GRSS + z.sps || image) + (1 + z.objective.score + z.perceived.score ||
##     gorilla.id)
##    Data: analysis.data
##
##      AIC      BIC   logLik deviance df.resid
##  13622.7  13735.0  -6796.3  13592.7    13211
##
## Scaled residuals:
##     Min      1Q  Median      3Q     Max
## -3.3837 -0.6167 -0.3470  0.7157  6.1835
##
## Random effects:
##  Groups       Name              Variance Std.Dev.
##  gorilla.id   z.perceived.score 0.04405  0.2099
##  gorilla.id.1 z.objective.score 0.01444  0.1202
##  gorilla.id.2 (Intercept)       2.12951  1.4593
##  image        z.sps             0.03225  0.1796
##  image.1      z.GRSS            0.01543  0.1242
##  image.2      (Intercept)       0.24920  0.4992
## Number of obs: 13226, groups:  gorilla.id, 166; image, 80
##
## Fixed effects:
##                           Estimate Std. Error z value Pr(>|z|)
## (Intercept)              -1.231002   0.129864  -9.479  < 2e-16 ***
## z.GRSS                    0.125427   0.149896   0.837  0.40272
## z.objective.score        -0.132960   0.065978  -2.015  0.04388 *
## z.perceived.score        -0.191640   0.067572  -2.836  0.00457 **
## z.sps                    -0.238817   0.155953  -1.531  0.12568
## z.GRSS:z.objective.score -0.008296   0.037416  -0.222  0.82452
## z.GRSS:z.perceived.score -0.179163   0.042123  -4.253 2.11e-05 ***
## z.objective.score:z.sps   0.035128   0.043804   0.802  0.42259
## z.perceived.score:z.sps   0.039332   0.048469   0.811  0.41709
## ---
```

```
## Signif. codes:  0 '***' 0.001 '**' 0.01 '*' 0.05 '.' 0.1 ' ' 1
##
## Correlation of Fixed Effects:
##             (Intr) z.GRSS z.bjc. z.prc. z.sps  z.GRSS:z.b. z.GRSS:z.p. z.b.:.
## z.GRSS      -0.030
## z.bjctv.scr  0.004  0.000
## z.prcvd.scr  0.006  0.003 -0.344
## z.sps        0.027 -0.623  0.000  0.000
## z.GRSS:z.b.  0.000  0.009 -0.021  0.010 -0.006
## z.GRSS:z.p.  0.008  0.020  0.009 -0.019 -0.013 -0.263
## z.bjctv.s:. -0.001 -0.006  0.020 -0.009  0.007 -0.513       0.131
## z.prcvd.s:. -0.001 -0.012 -0.008  0.024  0.020  0.133      -0.535      -0.285
## optimizer (Nelder_Mead) convergence code: 0 (OK)
## Model failed to converge with max|grad| = 0.00898361 (tol = 0.002, component 1)
```

GRSS - Seperate models for objective and perceived scores of gender typicalty

```
model1 <- glmer(response ~ z.GRSS*z.objective.score +
                             (1 + z.GRSS | image) +
                             (1 + z.objective.score | gorilla.id), data = analysis.data, family = "binon
summary(model1)
```

```
## Generalized linear mixed model fit by maximum likelihood (Laplace
##   Approximation) [glmerMod]
##  Family: binomial  ( logit )
## Formula: response ~ z.GRSS * z.objective.score + (1 + z.GRSS | image) +
##     (1 + z.objective.score | gorilla.id)
##    Data: analysis.data
##
##      AIC      BIC   logLik deviance df.resid
##  14497.8  14573.1  -7238.9  14477.8    13868
##
## Scaled residuals:
##     Min      1Q  Median      3Q     Max
## -3.5322 -0.6287 -0.3636  0.7571  5.4186
##
## Random effects:
##  Groups     Name              Variance Std.Dev. Corr
##  gorilla.id (Intercept)       1.96195  1.4007
##             z.objective.score 0.01995  0.1412   0.09
##  image      (Intercept)       0.24690  0.4969
##             z.GRSS            0.04812  0.2194   0.03
## Number of obs: 13878, groups:  gorilla.id, 175; image, 80
##
## Fixed effects:
##                          Estimate Std. Error z value Pr(>|z|)
## (Intercept)              -1.17626    0.12293  -9.568  < 2e-16 ***
## z.GRSS                   -0.02300    0.11246  -0.205  0.83794
## z.objective.score        -0.20011    0.06244  -3.205  0.00135 **
## z.GRSS:z.objective.score -0.03574    0.03532  -1.012  0.31160
## ---
## Signif. codes:  0 '***' 0.001 '**' 0.01 '*' 0.05 '.' 0.1 ' ' 1
```

```
##
## Correlation of Fixed Effects:
##              (Intr) z.GRSS z.bjc.
## z.GRSS      -0.012
## z.bjctv.scr  0.023  0.002
## z.GRSS:z.b.  0.003  0.038  0.024
```

```
model2 <- glmer(response ~ z.GRSS*z.perceived.score +
                             (1 + z.GRSS | image) +
                             (1 + z.perceived.score | gorilla.id), data = analysis.data, family = "binom
summary(model2)
```

```
## Generalized linear mixed model fit by maximum likelihood (Laplace
##   Approximation) [glmerMod]
##  Family: binomial  ( logit )
## Formula: response ~ z.GRSS * z.perceived.score + (1 + z.GRSS | image) +
##     (1 + z.perceived.score | gorilla.id)
##    Data: analysis.data
##
##      AIC      BIC   logLik deviance df.resid
##  14452.8  14528.2  -7216.4  14432.8    13868
##
## Scaled residuals:
##     Min      1Q  Median      3Q     Max
## -3.4467 -0.6261 -0.3589  0.7544  5.4150
##
## Random effects:
##  Groups     Name              Variance Std.Dev. Corr
##  gorilla.id (Intercept)       1.99655  1.4130
##             z.perceived.score 0.04531  0.2129   0.26
##  image      (Intercept)       0.23553  0.4853
##             z.GRSS            0.02763  0.1662   -0.29
## Number of obs: 13878, groups:  gorilla.id, 175; image, 80
##
## Fixed effects:
##                          Estimate Std. Error z value Pr(>|z|)
## (Intercept)              -1.18559    0.12324  -9.620  < 2e-16 ***
## z.GRSS                   -0.02363    0.11235  -0.210    0.833
## z.perceived.score        -0.25109    0.06254  -4.015 5.95e-05 ***
## z.GRSS:z.perceived.score -0.14885    0.03422  -4.349 1.37e-05 ***
## ---
## Signif. codes:  0 '***' 0.001 '**' 0.01 '*' 0.05 '.' 0.1 ' ' 1
##
## Correlation of Fixed Effects:
##             (Intr) z.GRSS z.prc.
## z.GRSS      -0.035
## z.prcvd.scr  0.074  0.007
## z.GRSS:z.p.  0.008  0.147 -0.133
```

SPS - Seperate models for objective and perceived scores of gender typicalty

```r
model3 <- glmer(response ~ z.sps*z.objective.score +
                            (1 + z.sps | image) +
                            (1 + z.objective.score | gorilla.id), data = analysis.data, family = "binom
summary(model3)
```

```
## Generalized linear mixed model fit by maximum likelihood (Laplace
##   Approximation) [glmerMod]
##  Family: binomial  ( logit )
## Formula: response ~ z.sps * z.objective.score + (1 + z.sps | image) +
##     (1 + z.objective.score | gorilla.id)
##    Data: analysis.data
##
##      AIC      BIC   logLik deviance df.resid
##  13679.4  13754.3  -6829.7  13659.4    13216
##
## Scaled residuals:
##     Min      1Q  Median      3Q     Max
## -3.1102 -0.6238 -0.3541  0.7458  5.6292
##
## Random effects:
##  Groups     Name              Variance Std.Dev. Corr
##  gorilla.id (Intercept)       2.09515  1.4475
##             z.objective.score 0.02386  0.1545   0.07
##  image      (Intercept)       0.27108  0.5207
##             z.sps             0.04744  0.2178   -0.30
## Number of obs: 13226, groups:  gorilla.id, 166; image, 80
##
## Fixed effects:
##                          Estimate Std. Error z value Pr(>|z|)
## (Intercept)             -1.214839   0.130022  -9.343  < 2e-16 ***
## z.sps                   -0.150456   0.121615  -1.237  0.21603
## z.objective.score       -0.202684   0.065646  -3.088  0.00202 **
## z.sps:z.objective.score -0.003906   0.038812  -0.101  0.91984
## ---
## Signif. codes:  0 '***' 0.001 '**' 0.01 '*' 0.05 '.' 0.1 ' ' 1
##
## Correlation of Fixed Effects:
##              (Intr) z.sps  z.bjc.
## z.sps        -0.017
## z.bjctv.scr   0.022  0.004
## z.sps:z.bj.   0.007  0.040 -0.118
```

```r
model4 <- glmer(response ~ z.sps*z.perceived.score +
                            (1 + z.sps | image) +
                            (1 + z.perceived.score | gorilla.id), data = analysis.data, family = "binom
summary(model4)
```

```
## Generalized linear mixed model fit by maximum likelihood (Laplace
##   Approximation) [glmerMod]
##  Family: binomial  ( logit )
## Formula: response ~ z.sps * z.perceived.score + (1 + z.sps | image) +
##     (1 + z.perceived.score | gorilla.id)
```

```
##    Data: analysis.data
##
##      AIC      BIC   logLik deviance df.resid
##  13634.9  13709.8  -6807.4  13614.9    13216
##
## Scaled residuals:
##     Min      1Q  Median      3Q     Max
## -3.0498 -0.6205 -0.3469  0.7325  5.6025
##
## Random effects:
##  Groups     Name              Variance Std.Dev. Corr
##  gorilla.id (Intercept)       2.14831  1.4657
##             z.perceived.score 0.06529  0.2555   0.24
##  image      (Intercept)       0.25929  0.5092
##             z.sps             0.04230  0.2057   -0.50
## Number of obs: 13226, groups:  gorilla.id, 166; image, 80
##
## Fixed effects:
##                          Estimate Std. Error z value Pr(>|z|)
## (Intercept)              -1.22961    0.13083  -9.399  < 2e-16 ***
## z.sps                    -0.15313    0.12308  -1.244   0.2135
## z.perceived.score        -0.26309    0.06653  -3.954 7.68e-05 ***
## z.sps:z.perceived.score -0.08414    0.04219  -1.994   0.0462 *
## ---
## Signif. codes:  0 '***' 0.001 '**' 0.01 '*' 0.05 '.' 0.1 ' ' 1
##
## Correlation of Fixed Effects:
##             (Intr) z.sps  z.prc.
## z.sps       -0.028
## z.prcvd.scr  0.080  0.012
## z.sps:z.pr.  0.016  0.155 -0.190
```

Gender

Men and women rating all faces

```
gender.model1 <- glmer(response ~ z.GRSS*z.perceived.score*e.gender +
                           (1 + z.GRSS + e.gender || image) +
                           (1 + z.perceived.score || gorilla.id), data = analysis.data, family = "binomi

summary(gender.model1)
```

```
## Generalized linear mixed model fit by maximum likelihood (Laplace
##   Approximation) [glmerMod]
##  Family: binomial  ( logit )
## Formula: response ~ z.GRSS * z.perceived.score * e.gender + (1 + z.GRSS +
##     e.gender || image) + (1 + z.perceived.score || gorilla.id)
##    Data: analysis.data
##
##      AIC      BIC   logLik deviance df.resid
##  12977.1  13073.5  -6475.5  12951.1    12345
##
```

```
## Scaled residuals:
##     Min      1Q  Median      3Q     Max
## -3.4143 -0.6262 -0.3665  0.7660  5.3021
##
## Random effects:
##  Groups       Name               Variance Std.Dev.
##  gorilla.id   z.perceived.score 0.04771  0.2184
##  gorilla.id.1 (Intercept)       1.65195  1.2853
##  image        e.gender          0.01212  0.1101
##  image.1      z.GRSS            0.02708  0.1646
##  image.2      (Intercept)       0.24770  0.4977
## Number of obs: 12358, groups:  gorilla.id, 156; image, 80
##
## Fixed effects:
##                                  Estimate Std. Error z value Pr(>|z|)
## (Intercept)                      -1.20886    0.12227  -9.887  < 2e-16 ***
## z.GRSS                            0.07381    0.10714   0.689    0.491
## z.perceived.score                -0.25531    0.06404  -3.987 6.70e-05 ***
## e.gender                         -0.32249    0.21718  -1.485    0.138
## z.GRSS:z.perceived.score         -0.13848    0.03553  -3.897 9.72e-05 ***
## z.GRSS:e.gender                  -0.05895    0.21105  -0.279    0.780
## z.perceived.score:e.gender        0.04392    0.06145   0.715    0.475
## z.GRSS:z.perceived.score:e.gender  0.06362    0.06014   1.058    0.290
## ---
## Signif. codes:  0 '***' 0.001 '**' 0.01 '*' 0.05 '.' 0.1 ' ' 1
##
## Correlation of Fixed Effects:
##             (Intr) z.GRSS z.prc. e.gndr z.GRSS:z.. z.GRSS:.g z.p.:.
## z.GRSS      -0.084
## z.prcvd.scr  0.009  0.002
## e.gender     0.121 -0.108  0.002
## z.GRSS:z.p.  0.005  0.022 -0.041 -0.002
## z.GRSS:.gnd -0.096 -0.040 -0.001 -0.096  0.005
## z.prcvd.s:.  0.002 -0.003  0.076  0.015 -0.116      0.003
## z.GRSS:..:. -0.003  0.006 -0.066  0.003 -0.009      0.024    -0.103
```

```
gender.model2 <- glmer(response ~ z.GRSS*z.objective.score*e.gender +
                          (1 + z.GRSS + e.gender | image) +
                          (1 + z.objective.score | gorilla.id), data = analysis.data, family = "binomi
```

```
## boundary (singular) fit: see help('isSingular')
```

```
summary(gender.model2)
```

```
## Generalized linear mixed model fit by maximum likelihood (Laplace
##   Approximation) [glmerMod]
##  Family: binomial  ( logit )
## Formula: response ~ z.GRSS * z.objective.score * e.gender + (1 + z.GRSS +
##     e.gender | image) + (1 + z.objective.score | gorilla.id)
##    Data: analysis.data
##
##      AIC      BIC   logLik deviance df.resid
##  13015.0  13141.1  -6490.5  12981.0    12341
```

```
##
## Scaled residuals:
##     Min      1Q  Median      3Q     Max
## -3.6482 -0.6289 -0.3691  0.7717  4.9676
##
## Random effects:
##  Groups     Name              Variance Std.Dev. Corr
##  gorilla.id (Intercept)       1.62831  1.2761
##             z.objective.score 0.02095  0.1448   0.07
##  image      (Intercept)       0.25756  0.5075
##             z.GRSS            0.04198  0.2049   -0.01
##             e.gender          0.01767  0.1329   -0.78  0.64
## Number of obs: 12358, groups:  gorilla.id, 156; image, 80
##
## Fixed effects:
##                                    Estimate Std. Error z value Pr(>|z|)
## (Intercept)                        -1.20148    0.12213  -9.838  < 2e-16 ***
## z.GRSS                              0.07653    0.10731   0.713  0.47573
## z.objective.score                  -0.20679    0.06458  -3.202  0.00136 **
## e.gender                           -0.30009    0.21596  -1.390  0.16465
## z.GRSS:z.objective.score           -0.03431    0.03517  -0.976  0.32919
## z.GRSS:e.gender                    -0.06977    0.20949  -0.333  0.73910
## z.objective.score:e.gender          0.10279    0.05550   1.852  0.06404 .
## z.GRSS:z.objective.score:e.gender  0.03288    0.05263   0.625  0.53211
## ---
## Signif. codes:  0 '***' 0.001 '**' 0.01 '*' 0.05 '.' 0.1 ' ' 1
##
## Correlation of Fixed Effects:
##             (Intr) z.GRSS z.bjc. e.gndr z.GRSS:z.. z.GRSS:.g z.b.:.
## z.GRSS      -0.085
## z.bjctv.scr  0.022  0.000
## e.gender     0.095 -0.098  0.002
## z.GRSS:z.b. -0.002  0.034 -0.044 -0.004
## z.GRSS:.gnd -0.096 -0.040 -0.003 -0.097 -0.001
## z.bjctv.s:.  0.006 -0.005 -0.101  0.042  0.016     -0.005
## z.GRSS:..:. -0.005 -0.002 -0.050 -0.004 -0.006      0.044    -0.110
## optimizer (Nelder_Mead) convergence code: 0 (OK)
## boundary (singular) fit: see help('isSingular')
```

```r
gender.model3 <- glmer(response ~ z.sps*z.perceived.score*e.gender +
                          (1 + z.sps + e.gender || image) +
                          (1 + z.perceived.score || gorilla.id), data = analysis.data, family = "binomi
```

```
## Warning in checkConv(attr(opt, "derivs"), opt$par, ctrl = control$checkConv, :
## Model failed to converge with max|grad| = 0.00420439 (tol = 0.002, component 1)
```

```r
summary(gender.model3)
```

```
## Generalized linear mixed model fit by maximum likelihood (Laplace
##   Approximation) [glmerMod]
##  Family: binomial  ( logit )
## Formula: response ~ z.sps * z.perceived.score * e.gender + (1 + z.sps +
##     e.gender || image) + (1 + z.perceived.score || gorilla.id)
```

```
##    Data: analysis.data
##
##      AIC      BIC   logLik deviance df.resid
##  12281.6  12377.5  -6127.8  12255.6    11773
##
## Scaled residuals:
##     Min      1Q  Median      3Q     Max
## -3.5501 -0.6225 -0.3602  0.7537  5.3563
##
## Random effects:
##  Groups       Name              Variance Std.Dev.
##  gorilla.id   z.perceived.score 0.066844 0.25854
##  gorilla.id.1 (Intercept)       1.660692 1.28868
##  image        e.gender          0.004731 0.06878
##  image.1      z.sps             0.038356 0.19585
##  image.2      (Intercept)       0.270481 0.52008
## Number of obs: 11786, groups:  gorilla.id, 148; image, 80
##
## Fixed effects:
##                                  Estimate Std. Error z value Pr(>|z|)
## (Intercept)                      -1.14428    0.12907  -8.866  < 2e-16 ***
## z.sps                             0.11721    0.12178   0.962  0.33581
## z.perceived.score                -0.28011    0.06824  -4.105 4.05e-05 ***
## e.gender                         -0.35896    0.22979  -1.562  0.11826
## z.sps:z.perceived.score          -0.06780    0.04331  -1.565  0.11748
## z.sps:e.gender                   -0.72061    0.23999  -3.003  0.00268 **
## z.perceived.score:e.gender        0.03417    0.06861   0.498  0.61848
## z.sps:z.perceived.score:e.gender  0.09678    0.07438   1.301  0.19322
## ---
## Signif. codes:  0 '***' 0.001 '**' 0.01 '*' 0.05 '.' 0.1 ' ' 1
##
## Correlation of Fixed Effects:
##             (Intr) z.sps  z.prc. e.gndr z.s:.. z.sp:. z.p.:.
## z.sps        0.004
## z.prcvd.scr  0.010 -0.003
## e.gender     0.108 -0.264  0.003
## z.sps:z.pr. -0.003  0.011 -0.012  0.000
## z.sps:.gndr -0.235 -0.261  0.001  0.004  0.016
## z.prcvd.s:.  0.005 -0.001  0.074  0.015 -0.230 -0.006
## z.sps:z..:. -0.001  0.017 -0.136 -0.006 -0.106  0.012 -0.023
## optimizer (Nelder_Mead) convergence code: 0 (OK)
## Model failed to converge with max|grad| = 0.00420439 (tol = 0.002, component 1)
```

```
gender.model4 <- glmer(response ~ z.sps*z.objective.score*e.gender +
                           (1 + z.sps + e.gender || image) +
                           (1 + z.objective.score || gorilla.id), data = analysis.data, family = "binomi
```

```
## Warning in checkConv(attr(opt, "derivs"), opt$par, ctrl = control$checkConv, :
## Model failed to converge with max|grad| = 0.00895172 (tol = 0.002, component 1)
```

```
summary(gender.model4)
```

```
## Generalized linear mixed model fit by maximum likelihood (Laplace
```

```
##   Approximation) [glmerMod]
##  Family: binomial  ( logit )
## Formula: response ~ z.sps * z.objective.score * e.gender + (1 + z.sps +
##     e.gender || image) + (1 + z.objective.score || gorilla.id)
##    Data: analysis.data
##
##      AIC      BIC   logLik deviance df.resid
##  12317.2  12413.1  -6145.6  12291.2    11773
##
## Scaled residuals:
##     Min      1Q  Median      3Q     Max
## -3.4270 -0.6252 -0.3643  0.7635  5.3267
##
## Random effects:
##  Groups       Name              Variance  Std.Dev.
##  gorilla.id   z.objective.score 2.407e-02 0.1551498
##  gorilla.id.1 (Intercept)       1.630e+00 1.2767272
##  image        e.gender          6.954e-07 0.0008339
##  image.1      z.sps             4.094e-02 0.2023369
##  image.2      (Intercept)       2.861e-01 0.5348958
## Number of obs: 11786, groups:  gorilla.id, 148; image, 80
##
## Fixed effects:
##                                   Estimate Std. Error z value Pr(>|z|)
## (Intercept)                      -1.134037   0.128875  -8.800  < 2e-16 ***
## z.sps                             0.115413   0.120816   0.955  0.33943
## z.objective.score                -0.210014   0.066996  -3.135  0.00172 **
## e.gender                         -0.351899   0.227544  -1.547  0.12198
## z.sps:z.objective.score          -0.002645   0.038461  -0.069  0.94518
## z.sps:e.gender                   -0.722057   0.237804  -3.036  0.00239 **
## z.objective.score:e.gender        0.092495   0.056634   1.633  0.10243
## z.sps:z.objective.score:e.gender -0.002891   0.061678  -0.047  0.96262
## ---
## Signif. codes:  0 '***' 0.001 '**' 0.01 '*' 0.05 '.' 0.1 ' ' 1
##
## Correlation of Fixed Effects:
##             (Intr) z.sps  z.bjc. e.gndr z.s:.. z.sp:. z.b.:.
## z.sps        0.003
## z.bjctv.scr  0.006 -0.002
## e.gender     0.107 -0.264  0.001
## z.sps:z.bj. -0.002  0.009 -0.018  0.000
## z.sps:.gndr -0.233 -0.260  0.001  0.004  0.007
## z.bjctv.s:.  0.001 -0.001  0.067  0.012 -0.213 -0.005
## z.sps:z..:. -0.001  0.007 -0.112 -0.004 -0.073  0.010 -0.045
## optimizer (Nelder_Mead) convergence code: 0 (OK)
## Model failed to converge with max|grad| = 0.00895172 (tol = 0.002, component 1)
```

Men and women rating female faces

```
gender.model5 <- glmer(response ~ z.GRSS*z.perceived.score*e.gender +
                           (1 + z.GRSS + e.gender || image) +
                           (1 + z.perceived.score || gorilla.id), data = filter(analysis.data, Gender =
```

```
## Warning in checkConv(attr(opt, "derivs"), opt$par, ctrl = control$checkConv, :
## Model failed to converge with max|grad| = 0.00788029 (tol = 0.002, component 1)
```

```r
summary(gender.model5)
```

```
## Generalized linear mixed model fit by maximum likelihood (Laplace
##   Approximation) [glmerMod]
##  Family: binomial  ( logit )
## Formula: response ~ z.GRSS * z.perceived.score * e.gender + (1 + z.GRSS +
##     e.gender || image) + (1 + z.perceived.score || gorilla.id)
##    Data: filter(analysis.data, Gender == "F")
##
##      AIC      BIC   logLik deviance df.resid
##   6775.5   6862.9  -3374.7   6749.5     6159
##
## Scaled residuals:
##     Min      1Q  Median      3Q     Max
## -3.0254 -0.6356 -0.3706  0.7844  4.7857
##
## Random effects:
##  Groups       Name              Variance Std.Dev.
##  gorilla.id   z.perceived.score 0.11540  0.3397
##  gorilla.id.1 (Intercept)       1.39272  1.1801
##  image        e.gender          0.04317  0.2078
##  image.1      z.GRSS            0.02726  0.1651
##  image.2      (Intercept)       0.18532  0.4305
## Number of obs: 6172, groups:  gorilla.id, 155; image, 40
##
## Fixed effects:
##                                   Estimate Std. Error z value Pr(>|z|)
## (Intercept)                       -1.08489    0.12385  -8.760  < 2e-16 ***
## z.GRSS                             0.02463    0.10332   0.238 0.811570
## z.perceived.score                 -0.25929    0.08158  -3.178 0.001481 **
## e.gender                          -0.32343    0.20807  -1.554 0.120074
## z.GRSS:z.perceived.score          -0.18059    0.05080  -3.555 0.000378 ***
## z.GRSS:e.gender                    0.03812    0.19983   0.191 0.848701
## z.perceived.score:e.gender         0.08915    0.09296   0.959 0.337583
## z.GRSS:z.perceived.score:e.gender  0.12942    0.08634   1.499 0.133920
## ---
## Signif. codes:  0 '***' 0.001 '**' 0.01 '*' 0.05 '.' 0.1 ' ' 1
##
## Correlation of Fixed Effects:
##             (Intr) z.GRSS z.prc. e.gndr z.GRSS:z.. z.GRSS:.g z.p.:.
## z.GRSS      -0.073
## z.prcvd.scr  0.011  0.004
## e.gender     0.111 -0.109  0.002
## z.GRSS:z.p.  0.010  0.025 -0.036 -0.003
## z.GRSS:.gnd -0.093 -0.041 -0.002 -0.090  0.003
## z.prcvd.s:.  0.001 -0.004  0.074  0.015 -0.109      0.007
## z.GRSS:..:. -0.005  0.002 -0.074  0.007 -0.039      0.028    -0.078
## optimizer (Nelder_Mead) convergence code: 0 (OK)
## Model failed to converge with max|grad| = 0.00788029 (tol = 0.002, component 1)
```

```r
gender.model6 <- glmer(response ~ z.GRSS*z.objective.score*e.gender +
                           (1 + z.GRSS + e.gender || image) +
                           (1 + z.objective.score || gorilla.id), data = filter(analysis.data, Gender ==
```

```
## Warning in checkConv(attr(opt, "derivs"), opt$par, ctrl = control$checkConv, :
## Model failed to converge with max|grad| = 0.00495327 (tol = 0.002, component 1)
```

```r
summary(gender.model6)
```

```
## Generalized linear mixed model fit by maximum likelihood (Laplace
##   Approximation) [glmerMod]
##  Family: binomial  ( logit )
## Formula: response ~ z.GRSS * z.objective.score * e.gender + (1 + z.GRSS +
##     e.gender || image) + (1 + z.objective.score || gorilla.id)
##    Data: filter(analysis.data, Gender == "F")
##
##      AIC      BIC   logLik deviance df.resid
##   6816.7   6904.2  -3395.4   6790.7     6159
##
## Scaled residuals:
##     Min      1Q  Median      3Q     Max
## -3.2136 -0.6408 -0.3808  0.8281  4.5855
##
## Random effects:
##  Groups       Name              Variance Std.Dev.
##  gorilla.id   z.objective.score 0.03180  0.1783
##  gorilla.id.1 (Intercept)       1.35254  1.1630
##  image        e.gender          0.03439  0.1854
##  image.1      z.GRSS            0.04910  0.2216
##  image.2      (Intercept)       0.17926  0.4234
## Number of obs: 6172, groups:  gorilla.id, 155; image, 40
##
## Fixed effects:
##                                  Estimate Std. Error z value Pr(>|z|)
## (Intercept)                      -1.07032    0.12206  -8.769  < 2e-16 ***
## z.GRSS                            0.02656    0.10459   0.254  0.79956
## z.objective.score                -0.25026    0.07661  -3.267  0.00109 **
## e.gender                         -0.32184    0.20474  -1.572  0.11596
## z.GRSS:z.objective.score         -0.06637    0.04987  -1.331  0.18322
## z.GRSS:e.gender                   0.02648    0.19689   0.134  0.89302
## z.objective.score:e.gender        0.08880    0.07674   1.157  0.24720
## z.GRSS:z.objective.score:e.gender  0.02203    0.06977   0.316  0.75223
## ---
## Signif. codes:  0 '***' 0.001 '**' 0.01 '*' 0.05 '.' 0.1 ' ' 1
##
## Correlation of Fixed Effects:
##             (Intr) z.GRSS z.bjc. e.gndr z.GRSS:z.. z.GRSS:.g z.b.:.
## z.GRSS      -0.073
## z.bjctv.scr  0.012  0.001
## e.gender     0.112 -0.106  0.002
## z.GRSS:z.b.  0.003  0.020 -0.033 -0.002
## z.GRSS:.gnd -0.093 -0.038 -0.002 -0.092  0.001
```

```
## z.bjctv.s:.  0.001 -0.004  0.072  0.020 -0.086      0.001
## z.GRSS:..:. -0.004  0.001 -0.063  0.001 -0.004      0.028    -0.096
## optimizer (Nelder_Mead) convergence code: 0 (OK)
## Model failed to converge with max|grad| = 0.00495327 (tol = 0.002, component 1)
```

```
gender.model7 <- glmer(response ~ z.lw.global*z.perceived.score*e.gender +
                           (1 + z.lw.global + e.gender || image) +
                           (1 + z.perceived.score || gorilla.id), data = filter(analysis.data, Gender ==
```

```
## Warning in checkConv(attr(opt, "derivs"), opt$par, ctrl = control$checkConv, :
## Model failed to converge with max|grad| = 0.00561662 (tol = 0.002, component 1)
```

```
summary(gender.model7)
```

```
## Generalized linear mixed model fit by maximum likelihood (Laplace
##   Approximation) [glmerMod]
##  Family: binomial  ( logit )
## Formula: response ~ z.lw.global * z.perceived.score * e.gender + (1 +
##     z.lw.global + e.gender || image) + (1 + z.perceived.score ||
##     gorilla.id)
##    Data: filter(analysis.data, Gender == "F")
##
##      AIC      BIC   logLik deviance df.resid
##   6513.4   6600.3  -3243.7   6487.4     5919
##
## Scaled residuals:
##     Min      1Q  Median      3Q     Max
## -2.6163 -0.6383 -0.3652  0.7833  4.7390
##
## Random effects:
##  Groups       Name              Variance Std.Dev.
##  gorilla.id   z.perceived.score 0.13331  0.3651
##  gorilla.id.1 (Intercept)       1.45666  1.2069
##  image        e.gender          0.03430  0.1852
##  image.1      z.lw.global       0.02674  0.1635
##  image.2      (Intercept)       0.19029  0.4362
## Number of obs: 5932, groups:  gorilla.id, 149; image, 40
##
## Fixed effects:
##                                         Estimate Std. Error z value Pr(>|z|)
## (Intercept)                             -1.01620    0.13025  -7.802  6.1e-15 ***
## z.lw.global                              0.01911    0.11556   0.165  0.86863
## z.perceived.score                       -0.26869    0.08427  -3.189  0.00143 **
## e.gender                                -0.29312    0.22178  -1.322  0.18627
## z.lw.global:z.perceived.score           -0.10110    0.05593  -1.808  0.07066 .
## z.lw.global:e.gender                    -0.45191    0.22558  -2.003  0.04514 *
## z.perceived.score:e.gender               0.08870    0.09837   0.902  0.36721
## z.lw.global:z.perceived.score:e.gender   0.10524    0.09882   1.065  0.28690
## ---
## Signif. codes:  0 '***' 0.001 '**' 0.01 '*' 0.05 '.' 0.1 ' ' 1
##
## Correlation of Fixed Effects:
##             (Intr) z.lw.g z.prc. e.gndr z.l.:.. z.l.:. z.p.:.
```

```
## z.lw.global -0.008
## z.prcvd.scr  0.011  0.000
## e.gender     0.100 -0.256  0.003
## z.lw.glb:..  0.002  0.022 -0.008 -0.002
## z.lw.glbl:. -0.219 -0.204  0.000 -0.011  0.016
## z.prcvd.s:.  0.002 -0.003  0.069  0.014 -0.229  -0.002
## z.lw.g:..:. -0.003  0.015 -0.152 -0.001 -0.124   0.022 -0.016
## optimizer (Nelder_Mead) convergence code: 0 (OK)
## Model failed to converge with max|grad| = 0.00561662 (tol = 0.002, component 1)
```

```
gender.model8 <- glmer(response ~ z.lw.global*z.objective.score*e.gender +
                           (1 + z.lw.global + e.gender | image) +
                           (1 + z.objective.score | gorilla.id), data = filter(analysis.data, Gender ==
```

```
## Warning in checkConv(attr(opt, "derivs"), opt$par, ctrl = control$checkConv, :
## Model failed to converge with max|grad| = 0.00453368 (tol = 0.002, component 1)
```

```
summary(gender.model8)
```

```
## Generalized linear mixed model fit by maximum likelihood (Laplace
##   Approximation) [glmerMod]
##  Family: binomial  ( logit )
## Formula: response ~ z.lw.global * z.objective.score * e.gender + (1 +
##     z.lw.global + e.gender | image) + (1 + z.objective.score |      gorilla.id)
##    Data: filter(analysis.data, Gender == "F")
##
##      AIC      BIC   logLik deviance df.resid
##   6544.0   6657.7  -3255.0   6510.0     5915
##
## Scaled residuals:
##     Min      1Q  Median      3Q     Max
## -2.5622 -0.6544 -0.3726  0.8286  5.0808
##
## Random effects:
##  Groups     Name              Variance Std.Dev. Corr
##  gorilla.id (Intercept)       1.44003  1.2000
##             z.objective.score 0.04489  0.2119   0.60
##  image      (Intercept)       0.17411  0.4173
##             z.lw.global       0.02678  0.1637    0.01
##             e.gender          0.04889  0.2211   -0.81  0.58
## Number of obs: 5932, groups:  gorilla.id, 149; image, 40
##
## Fixed effects:
##                                        Estimate Std. Error z value Pr(>|z|)
## (Intercept)                            -1.01164    0.12833  -7.883 3.19e-15 ***
## z.lw.global                             0.01767    0.11518   0.153 0.878075
## z.objective.score                      -0.28493    0.07798  -3.654 0.000258 ***
## e.gender                               -0.27015    0.22164  -1.219 0.222899
## z.lw.global:z.objective.score          -0.00538    0.04837  -0.111 0.911434
## z.lw.global:e.gender                   -0.46144    0.22467  -2.054 0.039986 *
## z.objective.score:e.gender              0.05559    0.08442   0.658 0.510229
## z.lw.global:z.objective.score:e.gender -0.05385    0.08209  -0.656 0.511821
## ---
```

```
## Signif. codes:  0 '***' 0.001 '**' 0.01 '*' 0.05 '.' 0.1 ' ' 1
##
## Correlation of Fixed Effects:
##             (Intr) z.lw.g z.bjc. e.gndr z.l.:.. z.l.:. z.b.:.
## z.lw.global -0.007
## z.bjctv.scr  0.140 -0.001
## e.gender     0.036 -0.233  0.020
## z.lw.glb:.. -0.002  0.253 -0.001 -0.063
## z.lw.glbl:. -0.220 -0.198 -0.036 -0.012 -0.033
## z.bjctv.s:.  0.033 -0.070 -0.221  0.278 -0.057  -0.008
## z.lw.g:..:. -0.057 -0.037 -0.100 -0.007 -0.055   0.305 -0.020
## optimizer (Nelder_Mead) convergence code: 0 (OK)
## Model failed to converge with max|grad| = 0.00453368 (tol = 0.002, component 1)
```

Men and women rating male faces

```
gender.model9 <- glmer(response ~ z.GRSS*z.perceived.score*e.gender +
                          (1 + z.GRSS + e.gender || image) +
                          (1 + z.perceived.score || gorilla.id), data = filter(analysis.data, Gender =

summary(gender.model9)
```

```
## Generalized linear mixed model fit by maximum likelihood (Laplace
##   Approximation) [glmerMod]
##  Family: binomial  ( logit )
## Formula: response ~ z.GRSS * z.perceived.score * e.gender + (1 + z.GRSS +
##     e.gender || image) + (1 + z.perceived.score || gorilla.id)
##    Data: filter(analysis.data, Gender == "M")
##
##      AIC      BIC   logLik deviance df.resid
##   6331.4   6418.8  -3152.7   6305.4     6173
##
## Scaled residuals:
##     Min      1Q  Median      3Q     Max
## -3.2094 -0.5867 -0.3516  0.5995  5.2028
##
## Random effects:
##  Groups       Name              Variance  Std.Dev.
##  gorilla.id   z.perceived.score 4.513e-03 0.0671798
##  gorilla.id.1 (Intercept)       1.703e+00 1.3050969
##  image        e.gender          3.356e-08 0.0001832
##  image.1      z.GRSS            1.692e-02 0.1300641
##  image.2      (Intercept)       3.048e-01 0.5521032
## Number of obs: 6186, groups:  gorilla.id, 155; image, 40
##
## Fixed effects:
##                                   Estimate Std. Error z value Pr(>|z|)
## (Intercept)                     -1.303e+00  1.439e-01  -9.056  < 2e-16 ***
## z.GRSS                           1.443e-01  1.122e-01   1.286  0.19844
## z.perceived.score               -2.581e-01  9.529e-02  -2.709  0.00675 **
## e.gender                        -2.312e-01  2.265e-01  -1.021  0.30720
## z.GRSS:z.perceived.score        -9.627e-02  4.081e-02  -2.359  0.01834 *
```

```
## z.GRSS:e.gender                     -1.360e-01  2.205e-01  -0.617  0.53730
## z.perceived.score:e.gender          -7.578e-06  6.967e-02   0.000  0.99991
## z.GRSS:z.perceived.score:e.gender    1.898e-03  6.966e-02   0.027  0.97827
## ---
## Signif. codes:  0 '***' 0.001 '**' 0.01 '*' 0.05 '.' 0.1 ' ' 1
##
## Correlation of Fixed Effects:
##             (Intr) z.GRSS z.prc. e.gndr z.GRSS:z.. z.GRSS:.g z.p.:.
## z.GRSS      -0.079
## z.prcvd.scr  0.011  0.000
## e.gender     0.104 -0.109  0.002
## z.GRSS:z.p.  0.004  0.031 -0.040 -0.001
## z.GRSS:.gnd -0.086 -0.047 -0.001 -0.100  0.010
## z.prcvd.s:.  0.004 -0.003  0.063  0.026 -0.125      0.000
## z.GRSS:..:. -0.002  0.011 -0.053  0.001 -0.005      0.035    -0.132
```

```
gender.model10 <- glmer(response ~ z.GRSS*z.objective.score*e.gender +
                           (1 + z.GRSS + e.gender || image) +
                           (1 + z.objective.score || gorilla.id), data = filter(analysis.data, Gender ==
```

```
## Warning in checkConv(attr(opt, "derivs"), opt$par, ctrl = control$checkConv, :
## Model failed to converge with max|grad| = 0.0114183 (tol = 0.002, component 1)
```

```
summary(gender.model10)
```

```
## Generalized linear mixed model fit by maximum likelihood (Laplace
##   Approximation) [glmerMod]
##  Family: binomial  ( logit )
## Formula: response ~ z.GRSS * z.objective.score * e.gender + (1 + z.GRSS +
##     e.gender || image) + (1 + z.objective.score || gorilla.id)
##    Data: filter(analysis.data, Gender == "M")
##
##      AIC      BIC   logLik deviance df.resid
##   6332.0   6419.5  -3153.0   6306.0     6173
##
## Scaled residuals:
##     Min      1Q  Median      3Q     Max
## -3.1274 -0.5829 -0.3466  0.5971  5.2748
##
## Random effects:
##  Groups       Name              Variance  Std.Dev.
##  gorilla.id   z.objective.score 5.032e-02 0.224331
##  gorilla.id.1 (Intercept)       1.729e+00 1.315011
##  image        e.gender          1.280e-06 0.001131
##  image.1      z.GRSS            2.667e-02 0.163307
##  image.2      (Intercept)       3.528e-01 0.593929
## Number of obs: 6186, groups:  gorilla.id, 155; image, 40
##
## Fixed effects:
##                                      Estimate Std. Error z value Pr(>|z|)
## (Intercept)                         -1.315579   0.148577  -8.855   <2e-16 ***
## z.GRSS                               0.151415   0.113984   1.328    0.184
## z.objective.score                   -0.162648   0.103253  -1.575    0.115
```

```
## e.gender                           -0.225375   0.227963  -0.989    0.323
## z.GRSS:z.objective.score            0.004946   0.047232   0.105    0.917
## z.GRSS:e.gender                    -0.134162   0.221835  -0.605    0.545
## z.objective.score:e.gender          0.119353   0.079587   1.500    0.134
## z.GRSS:z.objective.score:e.gender  0.040646   0.078258   0.519    0.603
## ---
## Signif. codes:  0 '***' 0.001 '**' 0.01 '*' 0.05 '.' 0.1 ' ' 1
##
## Correlation of Fixed Effects:
##             (Intr) z.GRSS z.bjc. e.gndr z.GRSS:z.. z.GRSS:.g z.b.:.
## z.GRSS      -0.077
## z.bjctv.scr  0.006 -0.001
## e.gender     0.101 -0.108 -0.001
## z.GRSS:z.b. -0.002  0.010 -0.046 -0.002
## z.GRSS:.gnd -0.084 -0.048 -0.001 -0.101 -0.004
## z.bjctv.s:. -0.003 -0.002  0.061  0.014 -0.118     -0.004
## z.GRSS:..:. -0.004 -0.006 -0.055 -0.004 -0.016      0.010    -0.140
## optimizer (Nelder_Mead) convergence code: 0 (OK)
## Model failed to converge with max|grad| = 0.0114183 (tol = 0.002, component 1)
```

```
gender.model11 <- glmer(response ~ z.gm.global*z.perceived.score*e.gender +
                           (1 + z.gm.global + e.gender || image) +
                           (1 + z.perceived.score || gorilla.id), data = filter(analysis.data, Gender ==
```

```
## Warning in checkConv(attr(opt, "derivs"), opt$par, ctrl = control$checkConv, :
## Model failed to converge with max|grad| = 0.0136244 (tol = 0.002, component 1)
```

```
summary(gender.model11)
```

```
## Generalized linear mixed model fit by maximum likelihood (Laplace
##   Approximation) [glmerMod]
##  Family: binomial  ( logit )
## Formula: response ~ z.gm.global * z.perceived.score * e.gender + (1 +
##     z.gm.global + e.gender || image) + (1 + z.perceived.score ||
##     gorilla.id)
##    Data: filter(analysis.data, Gender == "M")
##
##      AIC      BIC   logLik deviance df.resid
##   6187.8   6275.0  -3080.9   6161.8     6053
##
## Scaled residuals:
##     Min      1Q  Median      3Q     Max
## -2.6435 -0.5838 -0.3540  0.5998  5.0501
##
## Random effects:
##  Groups       Name              Variance  Std.Dev.
##  gorilla.id   z.perceived.score 1.271e-02 0.112726
##  gorilla.id.1 (Intercept)       1.470e+00 1.212291
##  image        e.gender          7.752e-06 0.002784
##  image.1      z.gm.global       2.875e-02 0.169558
##  image.2      (Intercept)       3.038e-01 0.551179
## Number of obs: 6066, groups:  gorilla.id, 152; image, 40
##
```

```
## Fixed effects:
##                                        Estimate Std. Error z value Pr(>|z|)
## (Intercept)                            -1.21208    0.14155  -8.563  < 2e-16 ***
## z.gm.global                             0.34425    0.12081   2.850 0.004378 **
## z.perceived.score                      -0.29047    0.09605  -3.024 0.002493 **
## e.gender                               -0.36628    0.22136  -1.655 0.097990 .
## z.gm.global:z.perceived.score          -0.02859    0.04896  -0.584 0.559291
## z.gm.global:e.gender                   -0.90067    0.23668  -3.805 0.000142 ***
## z.perceived.score:e.gender             -0.03203    0.07425  -0.431 0.666246
## z.gm.global:z.perceived.score:e.gender  0.09790    0.08126   1.205 0.228289
## ---
## Signif. codes:  0 '***' 0.001 '**' 0.01 '*' 0.05 '.' 0.1 ' ' 1
##
## Correlation of Fixed Effects:
##             (Intr) z.gm.g z.prc. e.gndr z.g.:.. z.g.:. z.p.:.
## z.gm.global  0.009
## z.prcvd.scr  0.013 -0.007
## e.gender     0.100 -0.273  0.007
## z.gm.glb:.. -0.011  0.003 -0.017  0.001
## z.gm.glbl:. -0.209 -0.315  0.002  0.018  0.022
## z.prcvd.s:.  0.013  0.000  0.066  0.030 -0.231  -0.015
## z.gm.g:..:. -0.001  0.024 -0.107 -0.017 -0.174   0.002 -0.041
## optimizer (Nelder_Mead) convergence code: 0 (OK)
## Model failed to converge with max|grad| = 0.0136244 (tol = 0.002, component 1)
```

```
gender.model12 <- glmer(response ~ z.gm.global*z.objective.score*e.gender +
                           (1 + z.gm.global + e.gender || image) +
                           (1 + z.objective.score || gorilla.id), data = filter(analysis.data, Gender ==
```

```
## Warning in checkConv(attr(opt, "derivs"), opt$par, ctrl = control$checkConv, :
## Model failed to converge with max|grad| = 0.00333204 (tol = 0.002, component 1)
```

```
summary(gender.model12)
```

```
## Generalized linear mixed model fit by maximum likelihood (Laplace
##   Approximation) [glmerMod]
##  Family: binomial  ( logit )
## Formula: response ~ z.gm.global * z.objective.score * e.gender + (1 +
##     z.gm.global + e.gender || image) + (1 + z.objective.score ||
##     gorilla.id)
##    Data: filter(analysis.data, Gender == "M")
##
##      AIC      BIC   logLik deviance df.resid
##   6185.6   6272.8  -3079.8   6159.6     6053
##
## Scaled residuals:
##     Min      1Q  Median      3Q     Max
## -2.6502 -0.5794 -0.3475  0.6055  5.1217
##
## Random effects:
##  Groups       Name              Variance  Std.Dev.
##  gorilla.id   z.objective.score 5.162e-02 0.2271992
##  gorilla.id.1 (Intercept)       1.485e+00 1.2184893
```

```
##  image        e.gender          9.614e-08 0.0003101
##  image.1      z.gm.global       3.042e-02 0.1743999
##  image.2      (Intercept)       3.540e-01 0.5950009
## Number of obs: 6066, groups:  gorilla.id, 152; image, 40
##
## Fixed effects:
##                                          Estimate Std. Error z value Pr(>|z|)
## (Intercept)                             -1.220040   0.146286  -8.340  < 2e-16
## z.gm.global                              0.345388   0.121452   2.844  0.00446
## z.objective.score                       -0.163935   0.104006  -1.576  0.11498
## e.gender                                -0.353996   0.222254  -1.593  0.11122
## z.gm.global:z.objective.score           -0.007153   0.053022  -0.135  0.89268
## z.gm.global:e.gender                    -0.914184   0.237701  -3.846  0.00012
## z.objective.score:e.gender               0.138505   0.082644   1.676  0.09375
## z.gm.global:z.objective.score:e.gender   0.013337   0.089992   0.148  0.88218
##
## (Intercept)                             ***
## z.gm.global                             **
## z.objective.score
## e.gender
## z.gm.global:z.objective.score
## z.gm.global:e.gender                    ***
## z.objective.score:e.gender              .
## z.gm.global:z.objective.score:e.gender
## ---
## Signif. codes:  0 '***' 0.001 '**' 0.01 '*' 0.05 '.' 0.1 ' ' 1
##
## Correlation of Fixed Effects:
##             (Intr) z.gm.g z.bjc. e.gndr z.g.:.. z.g.:. z.b.:.
## z.gm.global  0.008
## z.bjctv.scr  0.005 -0.003
## e.gender     0.097 -0.272  0.000
## z.gm.glb:.. -0.004  0.001 -0.015  0.004
## z.gm.glbl:. -0.203 -0.315  0.002  0.018  0.007
## z.bjctv.s:. -0.003  0.004  0.060  0.012 -0.231  -0.008
## z.gm.g:..:.  0.003  0.007 -0.107 -0.007 -0.194   0.001 -0.039
## optimizer (Nelder_Mead) convergence code: 0 (OK)
## Model failed to converge with max|grad| = 0.00333204 (tol = 0.002, component 1)
```

Sexual orientation

```
so.model1 <- glmer(response ~ z.GRSS*z.perceived.score*e.so +
                           (1 + z.GRSS + e.so || image) +
                           (1 + z.perceived.score || gorilla.id), data = analysis.data, family = "binomi

summary(so.model1)
```

```
## Generalized linear mixed model fit by maximum likelihood (Laplace
##   Approximation) [glmerMod]
##  Family: binomial  ( logit )
## Formula: response ~ z.GRSS * z.perceived.score * e.so + (1 + z.GRSS +
##     e.so || image) + (1 + z.perceived.score || gorilla.id)
```

```
##    Data: analysis.data
##
##      AIC      BIC   logLik deviance df.resid
##  14150.4  14248.1  -7062.2  14124.4    13465
##
## Scaled residuals:
##     Min      1Q  Median      3Q     Max
## -3.3518 -0.6250 -0.3665  0.7604  6.1127
##
## Random effects:
##  Groups       Name              Variance  Std.Dev.
##  gorilla.id   z.perceived.score 0.0321265 0.17924
##  gorilla.id.1 (Intercept)       1.7978086 1.34082
##  image        e.so              0.2136231 0.46219
##  image.1      z.GRSS            0.0001834 0.01354
##  image.2      (Intercept)       0.2368402 0.48666
## Number of obs: 13478, groups:  gorilla.id, 170; image, 80
##
## Fixed effects:
##                              Estimate Std. Error z value Pr(>|z|)
## (Intercept)                  -1.22212    0.13883  -8.803  < 2e-16 ***
## z.GRSS                        0.03167    0.14197   0.223 0.823455
## z.perceived.score            -0.24460    0.06376  -3.836 0.000125 ***
## e.so                         -0.38466    0.26017  -1.478 0.139281
## z.GRSS:z.perceived.score     -0.08579    0.03543  -2.421 0.015465 *
## z.GRSS:e.so                   0.34699    0.28396   1.222 0.221706
## z.perceived.score:e.so       -0.20520    0.08260  -2.484 0.012986 *
## z.GRSS:z.perceived.score:e.so  0.01531    0.07081   0.216 0.828814
## ---
## Signif. codes:  0 '***' 0.001 '**' 0.01 '*' 0.05 '.' 0.1 ' ' 1
##
## Correlation of Fixed Effects:
##             (Intr) z.GRSS z.prc. e.so   z.GRSS:z.. z.GRSS:.s z.p.:.
## z.GRSS       0.244
## z.prcvd.scr  0.011  0.002
## e.so        -0.131 -0.540  0.002
## z.GRSS:z.p.  0.004  0.020  0.116 -0.013
## z.GRSS:e.so -0.507 -0.506 -0.007  0.260 -0.003
## z.prcvd.s:.  0.003 -0.010 -0.040  0.016 -0.436      0.003
## z.GRSS:..:. -0.012 -0.002 -0.283  0.004 -0.445      0.021     0.178
```

```
soplot.data <- analysis.data %>%
  group_by(image,Gender,z.perceived.score,e.so) %>%
  summarise(prop.response = mean(response)) %>%
  filter(!is.na(e.so))
```

```
## `summarise()` has grouped output by 'image', 'Gender', 'z.perceived.score'. You
## can override using the `.groups` argument.
```

```
ggplot(soplot.data,aes(x = z.perceived.score,y = prop.response,group = e.so,colour = as.factor(e.so))) +
  geom_point() +
  geom_smooth(method = "lm") +
  theme_classic() +
```

```r
  xlab("Perceived facial gender typicality") +
  ylab("Mean participant response") +
  labs(colour = "Participant sexual orientation") +
  scale_color_hue(labels = c("Non-heteroseuxal", "Heterosexual"))
```

```
## `geom_smooth()` using formula = 'y ~ x'

## Warning: Removed 2 rows containing non-finite outside the scale range
## (`stat_smooth()`).

## Warning: Removed 2 rows containing missing values or values outside the scale range
## (`geom_point()`).
```

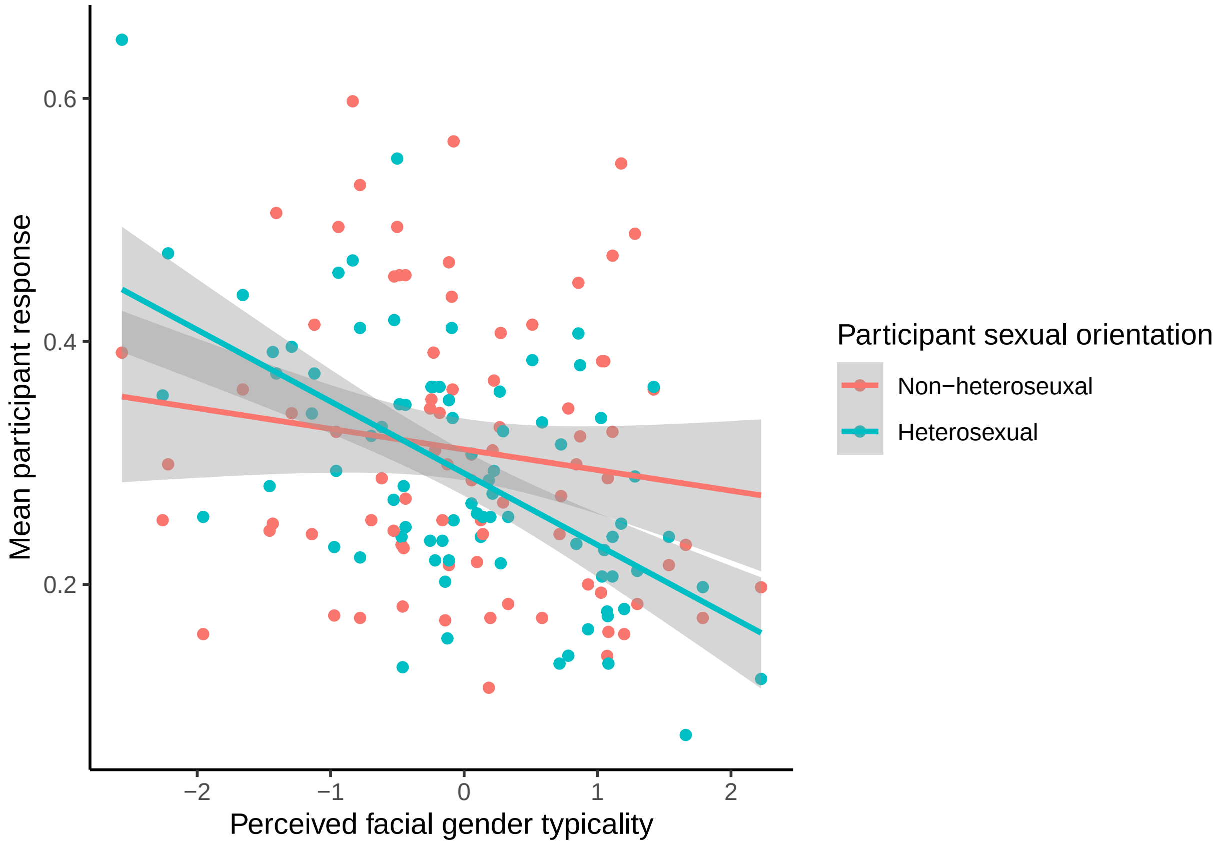


```r
so.model2 <- glmer(response ~ z.GRSS*z.objective.score*e.so +
                          (1 + z.GRSS + e.so || image) +
                          (1 + z.objective.score || gorilla.id), data = analysis.data, family = "binomi

summary(so.model2)
```

```
## Generalized linear mixed model fit by maximum likelihood (Laplace
##   Approximation) [glmerMod]
##  Family: binomial  ( logit )
## Formula: response ~ z.GRSS * z.objective.score * e.so + (1 + z.GRSS +
```

```
##     e.so || image) + (1 + z.objective.score || gorilla.id)
##    Data: analysis.data
##
##      AIC      BIC   logLik deviance df.resid
##  14185.3  14283.0  -7079.7  14159.3    13465
##
## Scaled residuals:
##     Min      1Q  Median      3Q     Max
## -3.2696 -0.6261 -0.3681  0.7661  5.8388
##
## Random effects:
##  Groups       Name              Variance Std.Dev.
##  gorilla.id   z.objective.score 0.017571 0.13256
##  gorilla.id.1 (Intercept)       1.788699 1.33742
##  image        e.so              0.259048 0.50897
##  image.1      z.GRSS            0.008567 0.09256
##  image.2      (Intercept)       0.252040 0.50204
## Number of obs: 13478, groups:  gorilla.id, 170; image, 80
##
## Fixed effects:
##                               Estimate Std. Error z value Pr(>|z|)
## (Intercept)                   -1.21990    0.13920  -8.764  < 2e-16 ***
## z.GRSS                         0.03647    0.14194   0.257  0.79722
## z.objective.score             -0.21317    0.06415  -3.323  0.00089 ***
## e.so                          -0.38214    0.26061  -1.466  0.14255
## z.GRSS:z.objective.score      -0.01402    0.03440  -0.408  0.68362
## z.GRSS:e.so                    0.34899    0.28315   1.233  0.21776
## z.objective.score:e.so        -0.09374    0.08276  -1.133  0.25731
## z.GRSS:z.objective.score:e.so  0.05902    0.06547   0.902  0.36732
## ---
## Signif. codes:  0 '***' 0.001 '**' 0.01 '*' 0.05 '.' 0.1 ' ' 1
##
## Correlation of Fixed Effects:
##             (Intr) z.GRSS z.bjc. e.so   z.GRSS:z.. z.GRSS:.s z.b.:.
## z.GRSS       0.242
## z.bjctv.scr  0.008  0.001
## e.so        -0.129 -0.536  0.002
## z.GRSS:z.b.  0.001  0.014  0.100 -0.010
## z.GRSS:e.so -0.504 -0.505 -0.005  0.258 -0.004
## z.bjctv.s:.  0.003 -0.007 -0.035  0.012 -0.390      0.001
## z.GRSS:..:. -0.009 -0.004 -0.263  0.002 -0.432      0.014     0.164
```

```
so.model3 <- glmer(response ~ z.sps*z.perceived.score*e.so +
                          (1 + z.sps + e.so || image) +
                          (1 + z.perceived.score || gorilla.id), data = analysis.data, family = "binomi
```

```
## Warning in checkConv(attr(opt, "derivs"), opt$par, ctrl = control$checkConv, :
## Model failed to converge with max|grad| = 0.00463304 (tol = 0.002, component 1)
```

```
summary(so.model3)
```

```
## Generalized linear mixed model fit by maximum likelihood (Laplace
##   Approximation) [glmerMod]
```

```
##  Family: binomial  ( logit )
## Formula: response ~ z.sps * z.perceived.score * e.so + (1 + z.sps + e.so ||
##     image) + (1 + z.perceived.score || gorilla.id)
##    Data: analysis.data
##
##      AIC      BIC   logLik deviance df.resid
##  13333.5  13430.4  -6653.7  13307.5    12813
##
## Scaled residuals:
##     Min      1Q  Median      3Q     Max
## -3.3395 -0.6189 -0.3571  0.7396  5.9656
##
## Random effects:
##  Groups       Name              Variance Std.Dev.
##  gorilla.id   z.perceived.score 0.03511  0.1874
##  gorilla.id.1 (Intercept)       1.90931  1.3818
##  image        e.so              0.18616  0.4315
##  image.1      z.sps             0.01035  0.1018
##  image.2      (Intercept)       0.26343  0.5133
## Number of obs: 12826, groups:  gorilla.id, 161; image, 80
##
## Fixed effects:
##                              Estimate Std. Error z value Pr(>|z|)
## (Intercept)                  -1.32618    0.17167  -7.725 1.12e-14 ***
## z.sps                        -0.22122    0.23244  -0.952  0.34124
## z.perceived.score            -0.30056    0.07008  -4.289 1.80e-05 ***
## e.so                         -0.19186    0.32642  -0.588  0.55670
## z.sps:z.perceived.score      -0.03885    0.05677  -0.684  0.49379
## z.sps:e.so                    0.60527    0.46412   1.304  0.19219
## z.perceived.score:e.so       -0.26732    0.09213  -2.902  0.00371 **
## z.sps:z.perceived.score:e.so  0.19801    0.11118   1.781  0.07493 .
## ---
## Signif. codes:  0 '***' 0.001 '**' 0.01 '*' 0.05 '.' 0.1 ' ' 1
##
## Correlation of Fixed Effects:
##             (Intr) z.sps  z.prc. e.so   z.s:.. z.sp:. z.p.:.
## z.sps        0.560
## z.prcvd.scr  0.011  0.002
## e.so        -0.397 -0.708  0.002
## z.sps:z.pr.  0.003  0.011  0.296 -0.011
## z.sps:e.so  -0.676 -0.817 -0.006  0.587 -0.005
## z.prcvd.s:.  0.004 -0.009 -0.170  0.016 -0.586  0.003
## z.sps:z..:. -0.010 -0.005 -0.394  0.003 -0.743  0.011  0.460
## optimizer (Nelder_Mead) convergence code: 0 (OK)
## Model failed to converge with max|grad| = 0.00463304 (tol = 0.002, component 1)
```

```
so.model4 <- glmer(response ~ z.sps*z.objective.score*e.so +
                           (1 + z.sps + e.so || image) +
                           (1 + z.objective.score || gorilla.id), data = analysis.data, family = "binomi

summary(so.model4)
```

```
## Generalized linear mixed model fit by maximum likelihood (Laplace
##   Approximation) [glmerMod]
```

```
##  Family: binomial  ( logit )
## Formula: response ~ z.sps * z.objective.score * e.so + (1 + z.sps + e.so ||
##     image) + (1 + z.objective.score || gorilla.id)
##    Data: analysis.data
##
##      AIC      BIC   logLik deviance df.resid
##  13364.1  13461.1  -6669.1  13338.1    12813
##
## Scaled residuals:
##     Min      1Q  Median      3Q     Max
## -3.2385 -0.6196 -0.3600  0.7489  5.9816
##
## Random effects:
##  Groups       Name              Variance Std.Dev.
##  gorilla.id   z.objective.score 0.01726  0.1314
##  gorilla.id.1 (Intercept)       1.89114  1.3752
##  image        e.so              0.26955  0.5192
##  image.1      z.sps             0.00461  0.0679
##  image.2      (Intercept)       0.27937  0.5286
## Number of obs: 12826, groups:  gorilla.id, 161; image, 80
##
## Fixed effects:
##                              Estimate Std. Error z value Pr(>|z|)
## (Intercept)                  -1.31949    0.17154  -7.692 1.45e-14 ***
## z.sps                        -0.22255    0.23130  -0.962 0.335958
## z.objective.score            -0.23106    0.06966  -3.317 0.000909 ***
## e.so                         -0.17839    0.32656  -0.546 0.584886
## z.sps:z.objective.score       0.01401    0.05121   0.274 0.784351
## z.sps:e.so                    0.60133    0.46216   1.301 0.193210
## z.objective.score:e.so       -0.13868    0.09231  -1.502 0.133030
## z.sps:z.objective.score:e.so  0.11634    0.10126   1.149 0.250600
## ---
## Signif. codes:  0 '***' 0.001 '**' 0.01 '*' 0.05 '.' 0.1 ' ' 1
##
## Correlation of Fixed Effects:
##             (Intr) z.sps  z.bjc. e.so   z.s:.. z.sp:. z.b.:.
## z.sps        0.558
## z.bjctv.scr  0.009  0.003
## e.so        -0.394 -0.705  0.000
## z.sps:z.bj.  0.004  0.010  0.272 -0.010
## z.sps:e.so  -0.673 -0.818 -0.005  0.585 -0.007
## z.bjctv.s:.  0.001 -0.007 -0.143  0.012 -0.538  0.003
## z.sps:z..:. -0.009 -0.007 -0.361  0.004 -0.750  0.010  0.416
```

Mediation

First, get random slope of perceived gender typicality for participants. Reverse code this such that higher scores represent greater use of gender atypical facial cues when making sexual orientation judgements.

```
model <- glmer(response ~ z.perceived.score +
                 (1 + z.perceived.score | gorilla.id) +
                 (1 | image),data = analysis.data,family = "binomial")
```

```
random.effects <- ranef(model)$gorilla.id %>%
  rownames_to_column("gorilla.id") %>%
  mutate( gorilla.id = as.numeric(gorilla.id),
    fga.effect = -c(scale((z.perceived.score + fixef(model)[2])))) %>%
  select(gorilla.id,fga.effect)

participant.data2 <- participant.data %>%
  left_join(random.effects,by = "gorilla.id")

cor(participant.data2 %>% select(fga.effect,z.GRSS,z.sps),use = "pairwise")
```

```
##            fga.effect    z.GRSS     z.sps
## fga.effect  1.0000000 0.3456925 0.1733779
## z.GRSS      0.3456925 1.0000000 0.6328436
## z.sps       0.1733779 0.6328436 1.0000000
```

Second, run a mediation analysis to test whether the association between GRSS and use of gender typicality cues is mediated by SPS. Results indicated no mediation (indirect effect = -.05, 95% CI = -.19, .08).

```
med.model <- mediate(fga.effect ~ z.GRSS + (z.sps),data = participant.data2)
```

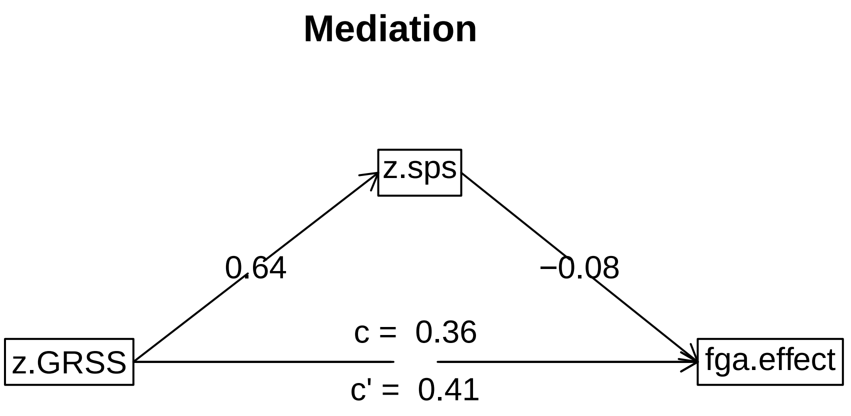


```
med.model
```

```
##
```

```
## Mediation/Moderation Analysis
## Call: mediate(y = fga.effect ~ z.GRSS + (z.sps), data = participant.data2)
##
## The DV (Y) was  fga.effect . The IV (X) was  z.GRSS . The mediating variable(s) =  z.sps .
##
## Total effect(c) of  z.GRSS  on  fga.effect  =  0.36   S.E. =  0.06  t  =  5.62  df=  217   with p =
## Direct effect (c') of  z.GRSS  on  fga.effect  removing  z.sps  =  0.41   S.E. =  0.08  t  =  4.95
## Indirect effect (ab) of  z.GRSS  on  fga.effect  through  z.sps   =  -0.05
## Mean bootstrapped indirect effect =  -0.05  with standard error =  0.07  Lower CI =  -0.2    Upper C
## R = 0.36 R2 = 0.13   F = 16.27 on 2 and 216 DF   p-value:  1.44e-09
##
##  To see the longer output, specify short = FALSE in the print statement or ask for the summary
```

Finally, run a mediation analysis to test whether the association between SPS and use of gender typicality cues is mediated by GRSS. Results indicated a significant full mediation (indirect effect = .26, 95% CI = .13, .42). See Figure below.

```
med.model <- mediate(fga.effect ~ z.sps + (z.GRSS),data = participant.data2,plot = FALSE)
med.model
```

```
##
## Mediation/Moderation Analysis
## Call: mediate(y = fga.effect ~ z.sps + (z.GRSS), data = participant.data2,
##     plot = FALSE)
##
## The DV (Y) was  fga.effect . The IV (X) was  z.sps . The mediating variable(s) =  z.GRSS .
##
## Total effect(c) of  z.sps  on  fga.effect  =  0.18   S.E. =  0.07  t  =  2.7  df=  217   with p =  0
## Direct effect (c') of  z.sps  on  fga.effect  removing  z.GRSS  =  -0.08   S.E. =  0.08  t  =  -0.99
## Indirect effect (ab) of  z.sps  on  fga.effect  through  z.GRSS   =  0.26
## Mean bootstrapped indirect effect =  0.26  with standard error =  0.07  Lower CI =  0.13    Upper CI
## R = 0.36 R2 = 0.13   F = 16.27 on 2 and 216 DF   p-value:  1.44e-09
##
##  To see the longer output, specify short = FALSE in the print statement or ask for the summary
```

```
mediate.diagram(med.model)
```

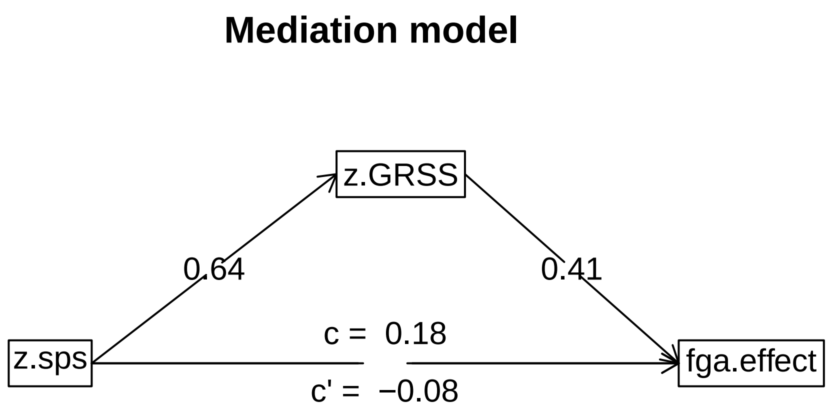


```r
library(diagram)
```

```
## Loading required package: shape
```

```r
data <- c(0,"'.66*'",0,
          0,0,0,
          "'.40*'","'.19* (-.07)'",0)
M <- matrix(nrow = 3,ncol = 3,byrow = TRUE,data = data)
plot <- plotmat(M,pos=c(1,2),
                name = c("Beliefs in Gender
Role Stereotypes","Sexual Prejudice","Use of gender
atypical facial cues"),
                box.type = "rect",box.size = .12,box.prop = .5,curve = 0,txt.font = 1)
```

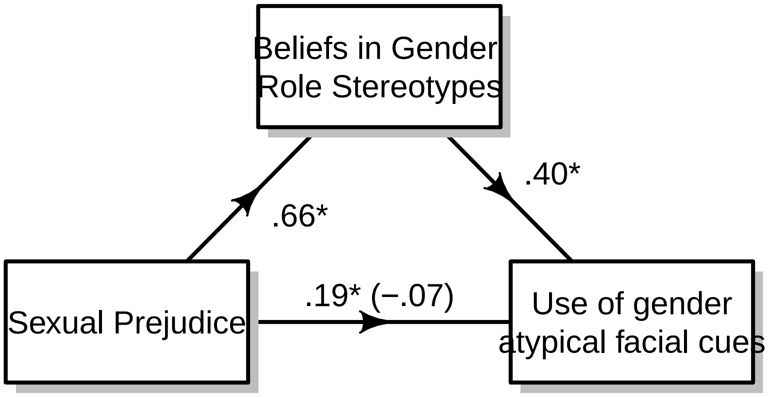
Beliefs in Gender Role Stereotypes
.66*
.40*
Sexual Prejudice
.19* (−.07)
Use of gender atypical facial cues
